# Metabolomics responses and tolerance of *Pseudomonas aeruginosa* under acoustic vibration stress

**DOI:** 10.1371/journal.pone.0297030

**Published:** 2024-01-29

**Authors:** Nawaporn Vinayavekhin, Thanyaporn Wattanophas, Mark Francis Murphy, Alisa S. Vangnai, Glyn Hobbs

**Affiliations:** 1 Center of Excellence in Natural Products Chemistry, Department of Chemistry, Faculty of Science, Chulalongkorn University, Bangkok, Thailand; 2 Center of Excellence in Biocatalyst and Sustainable Biotechnology, Faculty of Science, Chulalongkorn University, Bangkok, Thailand; 3 School of Pharmacy and Biomolecular Sciences, Liverpool John Moores University, Liverpool, United Kingdom; 4 Department of Biochemistry, Faculty of Science, Chulalongkorn University, Bangkok, Thailand; Yenepoya University, INDIA

## Abstract

Sound has been shown to impact microbial behaviors. However, our understanding of the chemical and molecular mechanisms underlying these microbial responses to acoustic vibration is limited. In this study, we used untargeted metabolomics analysis to investigate the effects of 100-Hz acoustic vibration on the intra- and extracellular hydrophobic metabolites of *P. aeruginosa* PAO1. Our findings revealed increased levels of fatty acids and their derivatives, quinolones, and *N*-acylethanolamines upon sound exposure, while rhamnolipids (RLs) showed decreased levels. Further quantitative real-time polymerase chain reaction experiments showed slight downregulation of the *rhlA* gene (1.3-fold) and upregulation of *fabY* (1.5-fold), *fadE* (1.7-fold), and *pqsA* (1.4-fold) genes, which are associated with RL, fatty acid, and quinolone biosynthesis. However, no alterations in the genes related to the *rpoS* regulators or quorum-sensing networks were observed. Supplementing sodium oleate to *P. aeruginosa* cultures to simulate the effects of sound resulted in increased tolerance of *P. aeruginosa* in the presence of sound at 48 h, suggesting a potential novel response-tolerance correlation. In contrast, adding RL, which went against the response direction, did not affect its growth. Overall, these findings provide potential implications for the control and manipulation of virulence and bacterial characteristics for medical and industrial applications.

## Introduction

Microorganisms are known to adapt their transcript, protein, or metabolite production in response to various stimuli and stresses in their environment for survival [1, 2]. The stimuli that have been extensively studied are primarily chemicals, including cell-to-cell communication signals used to manipulate organisms [3], as well as toxic chemicals or solvents used for the bioremediation of contaminants or as starting materials for biosynthesis [1]. In addition to these chemical stimuli, the mechanisms of microbial responses to other environmental conditions, such as changes in light, temperature, salinity, pH, and nutrient availability, have also been elucidated, as they have the ability to modulate microbial growth, virulence, and metabolite production [4–8].

Another stimulus that affects microbial physiology is acoustic vibration or sound waves. Studies have shown that acoustic vibration can alter various aspects of microbial behavior, including growth, antibiotic susceptibility, membrane permeability, intracellular calcium and potassium ion concentrations, and synthesis of RNA and proteins in *Chromobacterium violaceum*, *Escherichia coli*, *Serratia marcescens*, *Staphylococcus aureus*, *Streptococcus pyogenes*, *Saccharomyces cerevisiae*, and *Candida albicans* [9–11]. Metabolites linked to the quorum-sensing (QS) process, such as violacein and prodigiosin, were also found to differ in their amounts in sound-exposed samples of *Chromobacterium violaceum* and *Serratia marcescens*, respectively, as estimated by ultraviolet absorption [9]. Additionally, significant differences in volatile metabolite profiles were observed in *Saccharomyces cerevisiae* undergoing sound treatment [12]. Further studies have suggested that the direction and magnitude of these changes in microbial characteristics may depend on factors, such as the type of microorganism, frequency of the sound (either a single frequency or multiple frequencies in music), and intensity of the sound [9, 10, 13]. However, despite these findings, our understanding of microbial responses to acoustic vibration is still in its infancy, with a limited investigation into the chemical and molecular basis of these responses.

*Pseudomonas aeruginosa* is a ubiquitous Gram-negative opportunistic bacterial pathogen that is prevalent in most hospitals [14]. It can cause serious illness in patients on ventilators, as well as those with cystic fibrosis, burns, cancer, and immunodeficiency [15]. Its intricate QS communication networks are composed of at least three interconnected systems: Las, Rhl, and PQS [16]. In the Las and Rhl systems, the enzymes LasI and RhlI catalyze the synthesis of the signaling molecules *N*-(3-oxododecanoyl)-L-homoserine lactone (3-oxo-C_12_-HSL) and *N*-butyryl-L-homoserine lactone (C_4_-HSL), respectively [17–20]. Meanwhile, in the PQS system, the synthesis of 2-heptyl-3-hydroxy-4-quinolone, also known as the pseudomonas quinolone signal (PQS or C_n_-PQS (7:0)), is mediated by the enzyme products of the *pqsABCD* and *pqsH* gene clusters [21, 22]. These autoinducer molecules bind to their transcriptional regulators, LasR, RhlR, and PqsR, to modulate the target promoters of the genes involved in various processes, such as cell-to-cell communication, virulence, rhamnolipid production, biofilm formation, and swarming motility [23]. The Las system is often perceived as being at the top of the hierarchy, regulating the Rhl system, which in turn controls the PQS system [16]. However, certain environmental factors have been demonstrated to act through global regulators that govern this QS network and override this hierarchical order [23].

Previous research has shown that *P. aeruginosa* responded to acoustic vibration by stimulating growth and enhancing biofilm formation [13]. When cultured in culture dishes on top of vibration sources at frequencies of 100, 800, and 1600 Hz for 48 h, *P. aeruginosa* exhibited 0.3-, 2.8-, and 2.6-fold increases in biofilm formation, respectively, compared to the silence control. Interestingly, the biofilm formed in a concentric ring pattern, potentially in accordance with the nodes and antinodes of the standing wave, and was found to be affected by the amplitude of vibration. However, as is the case with other microorganisms, a more comprehensive understanding of the observed phenotype is still needed in order to manipulate or exploit this species for medical and industrial applications.

Untargeted metabolomics is a comparative method used for the global profiling of metabolites [24]. As this method enables the simultaneous detection of broad classes of metabolites, it can provide unbiased information about metabolites and metabolic pathways involved in the response to sound stimulus and help in deciphering a more comprehensive view of the mechanisms underlying the response.

Since previous research has demonstrated that 100 Hz vibration induces the smallest increase in biofilm formation in *P. aeruginosa* compared to other tested frequencies [13], we were interested in further exploring its effects at both the chemical and molecular levels. In this study, we employed untargeted metabolomics platforms to investigate changes in intracellular and extracellular hydrophobic metabolites in *P. aeruginosa* PAO1 (a laboratory reference strain) following a 48-h exposure to 100 Hz acoustic vibration, compared to a silence control. Subsequently, transcript levels of genes potentially associated with the alteration of metabolites were quantified using quantitative real-time polymerase chain reaction (qRT-PCR). Lastly, in order to better understand the adaptation, the effects of these metabolites on the growth fitness of *P. aeruginosa* under sound exposure were also evaluated.

## Materials and methods

### Vibration system and chemicals

A 100-Hz acoustic vibration was generated from a programmed Arduino board (serving as a sinusoidal waveform generator) connected to a 45-mm 0.2-W super-thin Mylar speaker, as previously employed [13]. Beef extract, yeast extract, and peptone were from HiMedia (Maharashtra, India). NaCl was from Ajax Finechem-Thermo Scientific (Rockford, IL, USA). CHCl_3_ was from RCI Labscan (Bangkok, Thailand). MeOH and ammonium hydroxide (25%) were from Merck (Rahway, NJ, USA). Isopropanol was obtained from Honeywell (Charlotte, NC, USA). Formic acid was from Sigma-Aldrich (Milwaukee, WI, USA). Rhamnolipids (R90; 90%) were obtained from AGAE Technologies-Sigma-Aldrich (Milwaukee, WI, USA), and sodium oleate from Tokyo Chemical Industry (Tokyo, Japan). All chemicals were reagent grade or liquid chromatography grade or better as appropriate and used as supplied.

### Bacterial strains and growth conditions

*Pseudomonas aeruginosa* PAO1 was kindly provided by Professor Junichi Kato, Hiroshima University. Bacteria were grown aerobically at 150 or 200 rpm, 30°C for 16–18 h in nutrient broth (NB; 1 g L^–1^ beef extract, 2 g L^–1^ yeast extract, 5 g L^–1^ peptone, and 5 g L^–1^ NaCl, pH 7.4) as reported previously [13]. The overnight cultures were diluted in fresh NB medium to an optical density at 600 nm (OD_600_) of 0.45–0.50. For metabolomics and qRT-PCR analyses, 20 mL of diluted culture was then added to each 60-mL culture bottle. For metabolite addition assay, the diluted culture was supplemented with indicated concentrations of metabolites or solvent vehicle (water) as control, before 1 mL was added to each 4-mL culture glass vial. Subsequently, the cultures were allowed to grow statically at 30°C with (on top of the speaker in an incubator) or without (in a separate incubator) 100 Hz acoustic vibration treatment for a predetermined time as indicated.

### Metabolites extraction

Metabolites extraction protocols were modified from those described previously [25]. After incubation with or without acoustic vibration for 48 h, bacterial cultures were vortexed briefly and vigorously to mix, and 700 *μ*L of cultures were collected for OD_600_ measurement. The remaining cultures were then centrifuged at 4,472*g*, 4°C for 15 min to separate bacterial cells and supernatant. The cell pellets were washed once with 20 mL of NB medium, resuspended in 3 mL of NB medium, and extracted with a mixture solution of 6 mL CHCl_3_ and 3 mL MeOH. The supernatant was extracted with a solution of 10 mL CHCl_3_ and 5 mL MeOH. Subsequently, the mixtures were centrifuged at 1,000*g*, 4°C for 5 min. The organic layer was collected, dried under a stream of nitrogen gas, and stored at ˗20°C. The samples were reconstituted in 200 *μ*L CHCl_3_ before liquid chromatography (LC)–mass spectrometry (MS) analysis.

### LC–MS and LC–MS/MS analyses

40 *μ*L of each sample was analyzed in both the positive and negative ion modes on an Ultimate DGP‐3600SD LC coupled to a Bruker MicrOTOF Q‐II MS instrument, as previously described [26]. However, some parameters were modified slightly to obtain optimal spectra. Specifically, the collision RF was set at 250 Vpp. The active exclusion was set to release ions after 0.30 min. Collision energies for ions with charge = 1 were set at 20 eV for *m/z* 500.00, 30 eV for *m/z* 1000.00, and 40 eV for *m/z* 2000.00. For other *m/z* values, the collision energies were automatically interpolated from those of the closest *m/z* values.

### LC–MS untargeted data analysis

The total ion chromatograms of the 100 Hz-exposed and unexposed control groups were obtained in two independent sets of triplicates, totaling 12 chromatograms for intracellular pellet samples and 12 chromatograms for extracellular supernatant samples in each ion mode. The comparative data analyses were then performed separately for the intracellular and extracellular samples, as described previously [26]. However, the criteria for identifying metabolite ions with altering levels upon exposure to 100-Hz vibration were changed to (i) a minimum fold change of ≥2, instead of ≥4, as there were no observable differences in OD_600_ of the two groups, and (ii) the minimum integrated mass ion intensity (MSII) in the elevated sample groups of 10,000, instead of 30,000, to allow for the depiction of more metabolites. The metabolite ions (defined by *m/z* and retention time) were included in the final list of changed metabolite ions only if they passed through the imposed filters for both sets of experiments.

### qRT-PCR analysis

1.5 mL of each *P. aeruginosa* culture was centrifuged at 16,000*g* for 1 min to collect cell pellets. Total RNA was isolated immediately from the cell pellets using an easy-spin^TM^ [DNA free] total RNA extraction kit (Intron Biotechnology, Seongnam, South Korea). In the last step, the total RNA was eluted from the binding column using 50 *μ*L of RNase-free water. Subsequently, 1 *μ*L of the total RNA was converted to cDNA using a Maxime RT premix kit with random primers (Intron Biotechnology). The obtained cDNA solution was then diluted 20-fold and used as a template in the qRT-PCR analysis using RealMOD^TM^ Green W^2^ 2x qPCR mix (Intron Biotechnology). The primer sequences (Bionics, Seoul, South Korea) for the amplification of the target and internal control (*proC* and *rpoD*) genes [27] are listed in S10 Table of S1 File. The final concentration of each primer was 0.5 *μ*M. The annealing temperature was 60°C, and the elongation time was 20 s. Unless specified earlier, all kits were applied according to the manufacturer’s instructions. Finally, the 2^–ΔΔCt^ method was used to calculate the relative mRNA expression levels of the target genes in the 100-Hz-exposed sample *vs*. the unexposed control. Student’s *t*-test was then applied to determine the statistical significance of the ΔC_t_ values between the sample groups, and only those with *p* < 0.05 were considered significant.

### Metabolite addition assay

For the construction of growth curves, the whole culture in each vial with or without 2 mM or 10 mM of sodium oleate, or 1.2 g L^–1^ or 6 g L^–1^ of rhamnolipids was collected at 6, 9, 24, or 48 h after incubation in the presence or absence of 100-Hz vibration. The sample was mixed by vortex to obtain a homogeneous culture, and the cell density was determined by OD_600_. Experiments were performed in triplicates or quadruplicates.

## Results

### Untargeted metabolomics of *P. aeruginosa* PAO1 under 100-Hz vibration

As previous work reported a slight enhancement in biofilm formation in *P. aeruginosa* PAO1 when grown in NB medium and exposed to 100 Hz acoustic vibration for 48 h [13], this condition was selected for detecting changes in metabolite composition and levels using untargeted metabolomics analysis. However, to ensure a consistent sound treatment condition for all three cultured replicates, PAO1 was cultured in glass bottles instead of Petri dishes used previously, allowing them to be placed simultaneously and symmetrically on top of a speaker connected to a vibration generator. The 100 Hz-exposed and unexposed cultures were incubated in separate incubators to maintain a silent environment for the control. Two independent sets of untargeted metabolomics analyses were also conducted to account for random factors and ensure the reproducibility of the detected changes in metabolite levels. After 48 h of incubation with or without sound, some cells were aggregated at the bottom, but no biofilm could be observed in any cultures. Subsequently, hydrophobic metabolites were extracted separately for the intra- (cell pellet) and extracellular (supernatant) components using a 2:1 (*v*/*v*) mixture of chloroform to methanol. The concentrated extracts were then analyzed using the LC–MS-based untargeted metabolomics platforms [25, 26].

**Fig 1:**
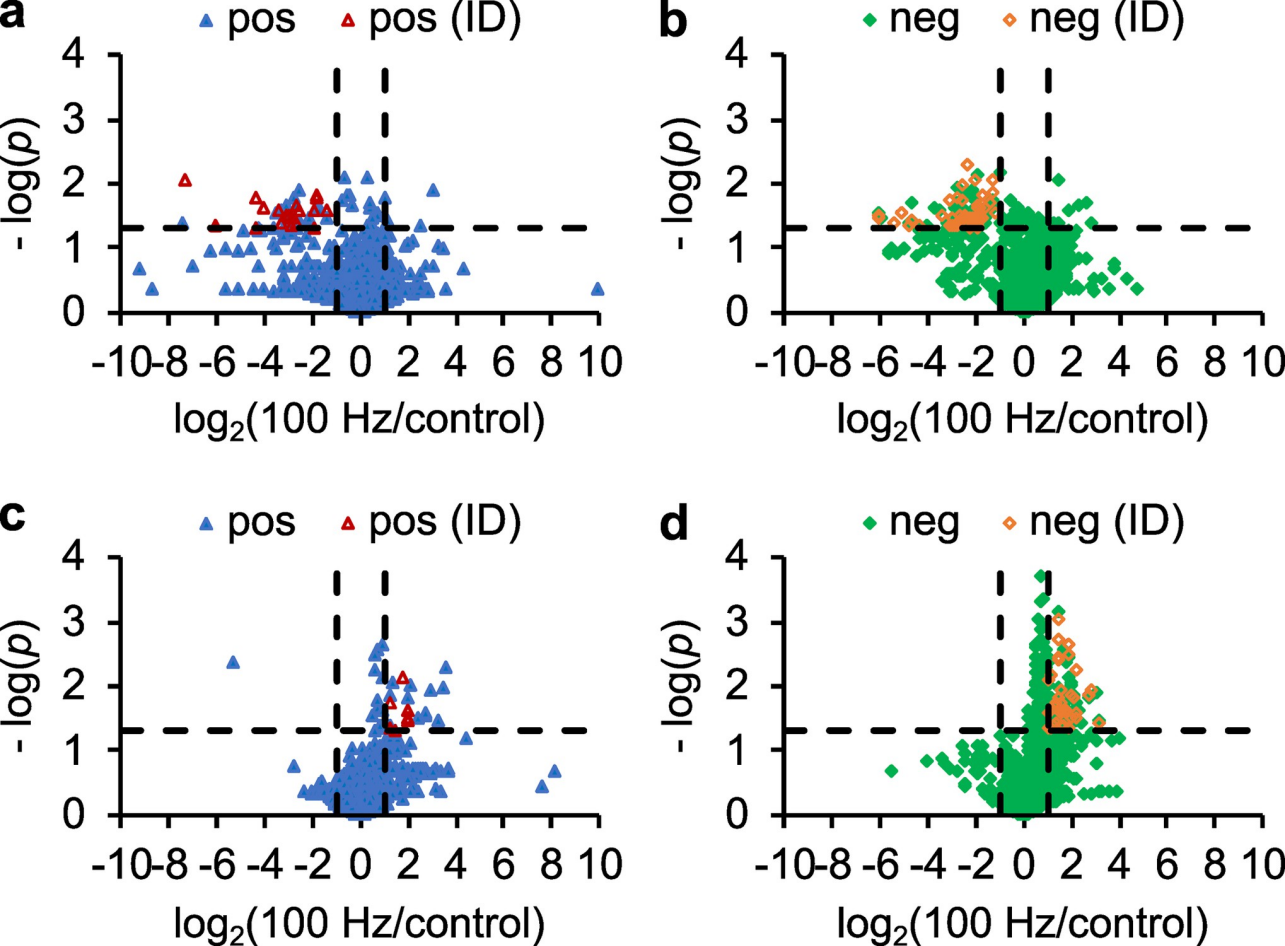
Volcano plots of differential metabolites in *Pseudomonas aeruginosa* at 48 h after 100-Hz acoustic vibration exposure. In the untargeted metabolomics analysis, the levels of each (a, b) extracellular and (c, d) intracellular metabolite ion in both the (a, c) positive (pos; *blue triangles*) and (b, d) negative (neg; *green diamonds*) ion modes were compared between sample groups with (100 Hz) or without (control) 100-Hz sound exposure. Each data point represents a metabolite ion with an average mass ion intensity (MSII) of at least 10,000 counts in the more abundant sample groups. Its *p*-value (*p*) from a Student’s *t*-test is plotted against the fold change of the 100-Hz-treated group over the control. Ions above the horizontal dash line have a *p*-value of less than 0.05. Those located outside the right and left vertical dash lines exhibit relative levels at least 2-fold higher in the 100-Hz group compared to the control and *vice versa*, respectively. These ions are considered changed metabolite ions. The changed metabolite ions, pos(ID) and neg(ID), represent the structurally characterized positive-mode (pos) and negative-mode (neg) ions in this study, depicted with *red open triangles* and *orange open diamonds*, respectively. Characterization was achieved through a combination of accurate masses, previously reported retention times, and tandem mass spectra.

To identify metabolites associated with 100 Hz vibration responses, relative levels of metabolite ions detected in the 100 Hz-treated and untreated LC–MS chromatograms were obtained using an XCMS program [28] and normalized by the OD_600_ value of each sample to eliminate slight variations in growth. The normalized levels of each metabolite ion in the treated samples were then compared with those in the untreated samples by calculating the fold changes. As no statistical difference was observed in the growth of the 100 Hz-treated and untreated *P. aeruginosa* cultures, metabolite ions were considered potentially associated with the vibration responses if their levels were increased or decreased by at least twofold with statistical significance (determined by Student’s *t*-test with *p* < 0.05). Using these criteria, 3 and 5 extracellular, and 12 and 37 intracellular metabolite ions were found to be increased, whereas 24 and 29 extracellular, and 1 and 0 intracellular metabolite ions were found to be decreased in the 100 Hz-treated samples in positive and negative ion modes, respectively, compared to the control without vibration (Fig 1 and S1–S7 Tables in S1 File).

**Fig 2:**
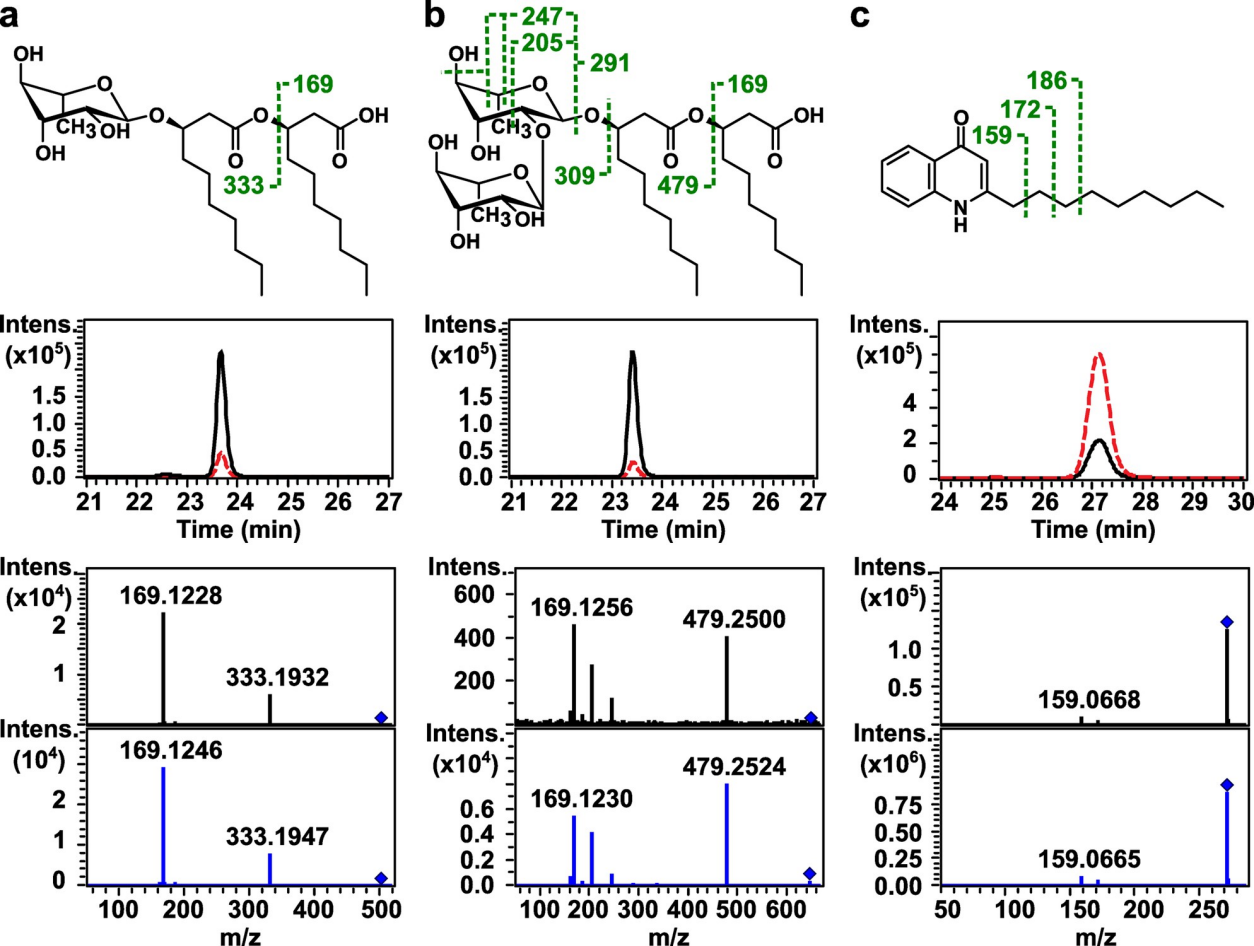
Chemical structures with proposed MS/MS fragments (top), extracted ion chromatograms (middle), and MS/MS spectra (bottom) of representative differential metabolites. The [M–H]^−^ions of the two most abundant detected rhamnolipids, (a) monorhamnolipid (10:0/10:0) and (b) dirhamnolipid (10:0/10:0), were decreased in their levels in the extracellular samples upon exposure to 100-Hz sound (middle; *black solid lines*), compared to the controls (middle; *red dashed lines*), whereas the [M + H]^+^ ion of (c) 2-alkyl-4-hydroxyquinoline (9:0) was elevated in its level in the intracellular samples. The structures of all representative metabolites were confirmed by comparing both the retention times and MS/MS spectra of samples (bottom; top spectra with *black lines*) with those of commercial standards (bottom; bottom spectra with *blue lines*). The proposed fragments observed in the MS/MS spectra are indicated with green dashed lines, along with the corresponding *m*/*z* values in the chemical structures.

Next, the chemical structures of the altered metabolite ions were manually characterized using accurate masses, previously reported retention time [25, 26], and tandem mass spectra (S1 Fig in S1 File). Out of the 111 metabolite ions with changed levels, probable structures could be assigned to 83 ions or 75%, which is relatively high for untargeted metabolomics analyses (S1–S7 Tables in S1 File). The results revealed elevated levels of fatty acids and their oxo derivatives in the extracellular components of the 100 Hz-treated samples compared to the silence controls, whereas monorhamnolipids (MRLs) and dirhamnolipids (DRLs) levels were found to decrease. Intracellularly, following 100 Hz sound treatment, the levels of fatty acids and their oxo and hydroxy derivatives were also increased, but their upregulation was accompanied by quinolones (2-alkyl-4-hydroxyquinoline *N*-oxide (AQNO), 2-alkyl-3-hydroxy-4-quinolone (C_n_-PQS), and 2-alkyl-4-hydroxyquinoline (AHQ)) and *N*-acylethanolamine (NAE) (Table 1 and Fig 2 and S1–S7 Tables in S1 File). Using accurate masses and retention time, we also searched our datasets for some other known metabolites, including *N*-(3-oxododecanoyl)-L-homoserine lactone (3-oxo-C_12_-HSL), phosphatidylethanolamine (PE), phosphatidylglycerol (PG), and diacylglycerol (DAG), and found that the levels of these metabolites changed either less than twofold or with statistical insignificance following acoustic vibration treatment, consistent with the results from untargeted analyses (Table 1 and S8 Table in S1 File). Lastly, metabolite standards of MRL, DRL, AHQ, C_n_-PQS, and NAE were acquired, and their retention time and tandem mass spectra were compared to those detected in the bacterial samples, which together further supported the prior structural assignment of these compounds (Fig 2 and S1 Fig in S1 File).

**Table 1:** Relative levels of represented extracellular and intracellular metabolites identified in the untargeted metabolomics analysis of *Pseudomonas aeruginosa* PAO1 grown statically with and without 100-Hz acoustic vibration treatment for 48 h.

Metabolite class and acyl chain			Ion	*m/z*	RT (min)	Fold^a^^,^ ^b^
Elevated metabolites in 100 Hz-treated extracellular samples						
	Fatty acid (FA)					
		16:1	[M–H]^–^	253.2198	19.4	13.5^†^
		18:1	[M–H]^–^	281.2509	20.2	9.2^†^
		oxo 16:1	[M–H]^–^	267.1972	17.4	3.6^*^
Decreased metabolites in 100 Hz-treated extracellular samples						
	Monorhamnolipid (MRL)					
		10:0/10:0	[M–H]^–^	503.3304	23.9	*8*.*0*^†^
		10:0/12:1	[M–H]^–^	529.3448	25.3	*7*.*4*^‡^
		10:0/12:0	[M–H]^–^	531.3601	26.2	*11*.*5*^‡^
	Dirhamnolipid (DRL)					
		10:0/10:0	[M–H]^–^	649.3862	23.7	*5*.4^*^
		10:0/12:1	[M–H]^–^	675.4021	25.0	*4*.4^*^
		10:0/12:0	[M–H]^–^	677.4191	25.9	*5*.2^*^
		12:0/12:0	[M–H]^–^	705.4486	28.2	*9*.*3*^†^
Elevated metabolites in 100 Hz-treated intracellular samples						
	Fatty acid (FA)^c^					
		16:0	[M–H]^–^	255.2336	19.7	3.4^‡^
		17:1	[M–H]^–^	267.2336	19.6	9.6^*^
		18:1	[M–H]^–^	281.2507	20.1	6.7^‡^
		19:1	[M–H]^–^	295.2653	20.3	7.3^‡^
		oxo 18:1	[M–H]^–^	295.2272	19.0	2.8^*^
		hydroxy 18:1	[M–H]^–^	297.2451	18.0	8.8^*^
		hydroxy 18:0	[M–H]^–^	299.2582	18.7	2.7^‡^
	Quinolines					
		AQNO (7:0)	[M–H]^–^	258.1484	13.7	2.3^*^
		AQNO (11:1)	[M–H]^–^	312.1961	18.1	2.8^*^
		C_n_-PQS (9:0)	[M–H]^–^	286.1785	29.9	2.1^*^
		AHQ (9:1)	[M + H]^+^	270.1867	27.1	4.0^*^
		AHQ (9:0)	[M + H]^+^	272.2028	27.2	3.4^*^
	*N*-acylethanolamine (NAE)					
		17:1	[M–H]^–^	310.2700	36.3	3.0^‡^
		18:2	[M–H]^–^	322.2713	36.0	3.4^‡^
		18:1	[M–H]^–^	324.2870	37.6	3.2^*^
		19:1	[M–H]^–^	338.3017	38.1	3.0^*^
Other extracellular metabolites (fold < 2 or *p* > 0.05)						
	3-oxo-C_12_-HSL		[M + H]^+^	298.2033	24.5	1.1
Other intracellular metabolites (fold < 2 or *p* > 0.05)						
	Phosphatidylethanolamine (PE)					
		16:0/18:1	[M–H]^–^	716.5180	44.7	1.1
		18:1/18:1	[M–H]^–^	742.5320	45.2	0.9
	Phosphatidylglycerol (PG)					
		16:0/18:1	[M–H]^–^	747.5151	39.2	1.0
		18:1/18:1	[M–H]^–^	773.5289	40.7	0.8
	Diacylglycerol (DAG)					
		16:0/18:1	[M + NH_4_]^+^	612.5544	44.4	1.7^*^
		18:1/18:1	[M + NH_4_]^+^	638.5699	44.7	1.6

Abbreviations: AHQ, 2-alkyl-4-hydroxyquinoline; AQNO, 2-alkyl-4-hydroxyquinoline *N*-oxide; C_n_-PQS, 2-alkyl-3-hydroxy-4-quinolone; *m/z*, mass-to-charge ratio; 3-oxo-C_12_-HSL, *N*-(3-oxododecanoyl)-L-homoserine lactone; RT, retention time

^a^ Fold value represents the ratio of the average mass ion intensity of 100 Hz-treated samples and that of the control and vice versa (in italics)

^b^ Student’s *t* test:

^*^, *p* < 0.05

^†^, *p* < 0.01

^‡^, *p* < 0.005; *N* = 3

^c^ Only representative, most abundant ions are presented here (see S1–S7 Tables in S1 File for a complete list)

### Expression levels of genes in biosynthetic pathways of changed metabolites and related in *P. aeruginosa*

**Fig 3:**
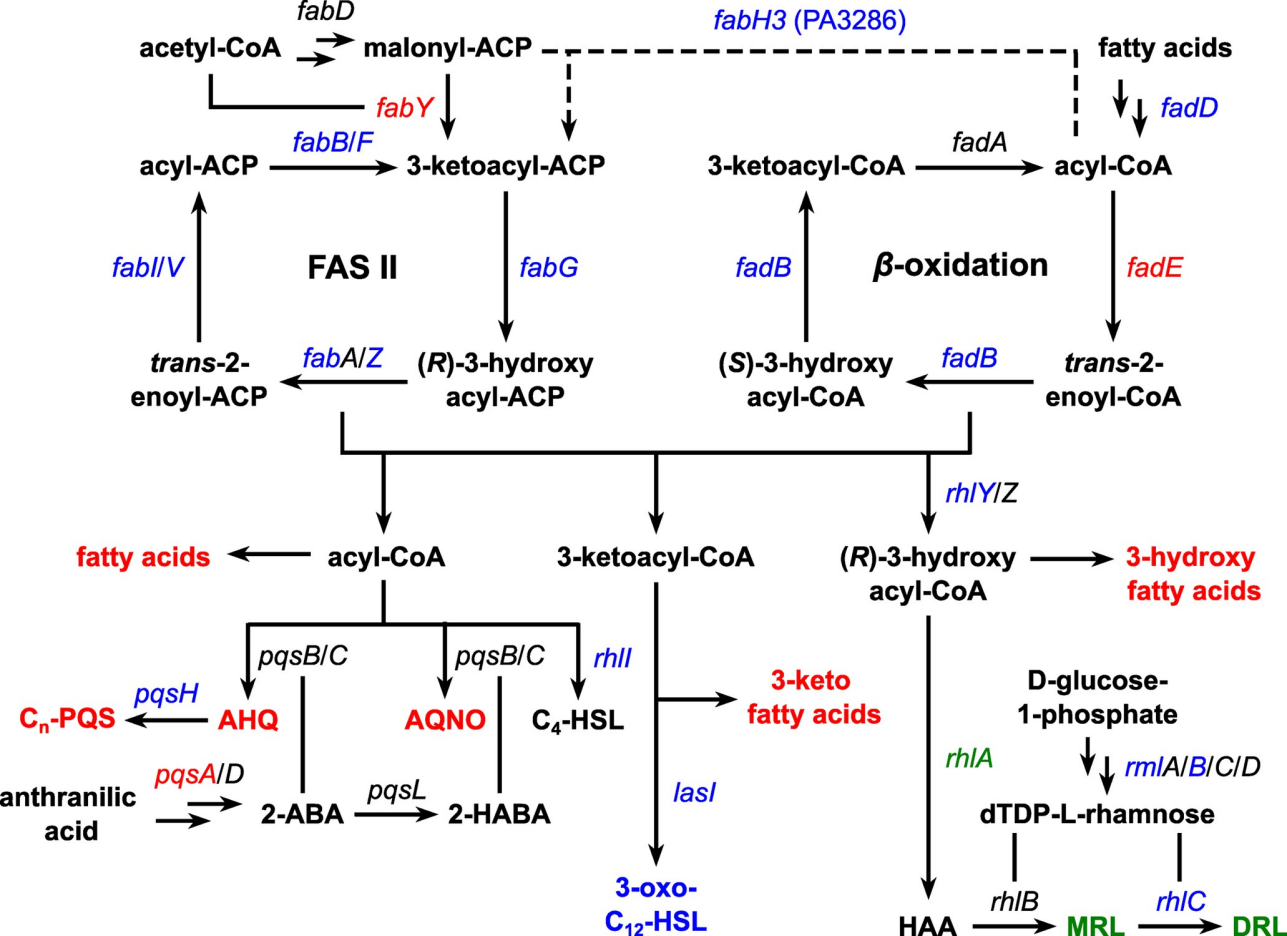
Biosynthetic pathways of differential and related metabolites in *Pseudomonas aeruginosa* and their interconnection. The key intermediates and enzyme-encoding genes are shown for the *de novo* fatty acid synthase (FAS II), β-oxidation, quinolones, *N*-acyl homoserine lactones, and rhamnolipids pathways. Metabolites or genes with elevated, decreased, or unchanged levels after 100-Hz sound exposure in the metabolomics analysis or quantitative real-time polymerase chain reaction are indicated by red, green, and blue letters, respectively. Abbreviations: 2-ABA, 2’-aminobenzoylacetate; 2-HABA, 2’-hydroxylaminobenzoylacetate; 3-oxo-C_12_-HSL, *N*-(3-oxododecanoyl)-L-homoserine lactone; ACP, acyl carrier protein; AHQ, 2-alkyl-4-hydroxyquinoline; AQNO, 2-alkyl-4-hydroxyquinoline *N*-oxide; C_4_-HSL, *N*-butyl-L-homoserine lactone; CoA, coenzyme A; C_n_-PQS, 2-alkyl-3-hydroxy-4-quinolone; DRL, dirhamnolipid; HAA, 3-(3-hydroxyalkanoyloxy)alkanoic acid; MRL, monorhamnolipid.

To decipher the mechanisms of acoustic vibration responses and to find the correlation between sound-stimulated metabolites and genes in their biosynthetic pathways, the expression levels of related genes were determined by qRT-PCR analysis on the total mRNA isolated from *P. aeruginosa* PAO1 grown under the same conditions as those for the untargeted metabolomics experiments. Specifically, the relative mRNA expression levels were quantitated for genes in the *de novo* fatty acid synthase (*fabB*, *fabF*, *fabG*, *fabH3*, *fabI*, *fabV*, *fabY*, *fabZ*), the fatty acid β-oxidation (*fadB1*, *fadB4*, *fadB5*, *fadD1*, *fadD2*, *fadD4*, *fadE*), the rhamnolipid biosynthetic (*rhlA*, *rhlC*, *rhlY*, *rmlB*), and the quinolone biosynthetic (*pqsA*, *pqsH*) pathways (Fig 3) [29–36]. Although there are other genes involved in these pathways as well, some of the genes were selected based on the previously-described existing activities (*e*.*g*., *fadB1*/*4*/*5*) [30, 31, 37] or on the assumption that the studied genes could represent other genes in the same gene clusters, which are regulated by the same operons (*e*.*g*., *pqsA* as a representative of the *pqsABCDE* operon) [38–43]. In addition to these genes, expression levels of genes associated with the QS communication networks (*lasI*, *lasR*, *rhlI*, *rhlR*, *pqsR*) and a regulator of environmental stress responses, biofilm formation, and virulence (*rpoS*) [44, 45] were also examined.

**Fig 4:**
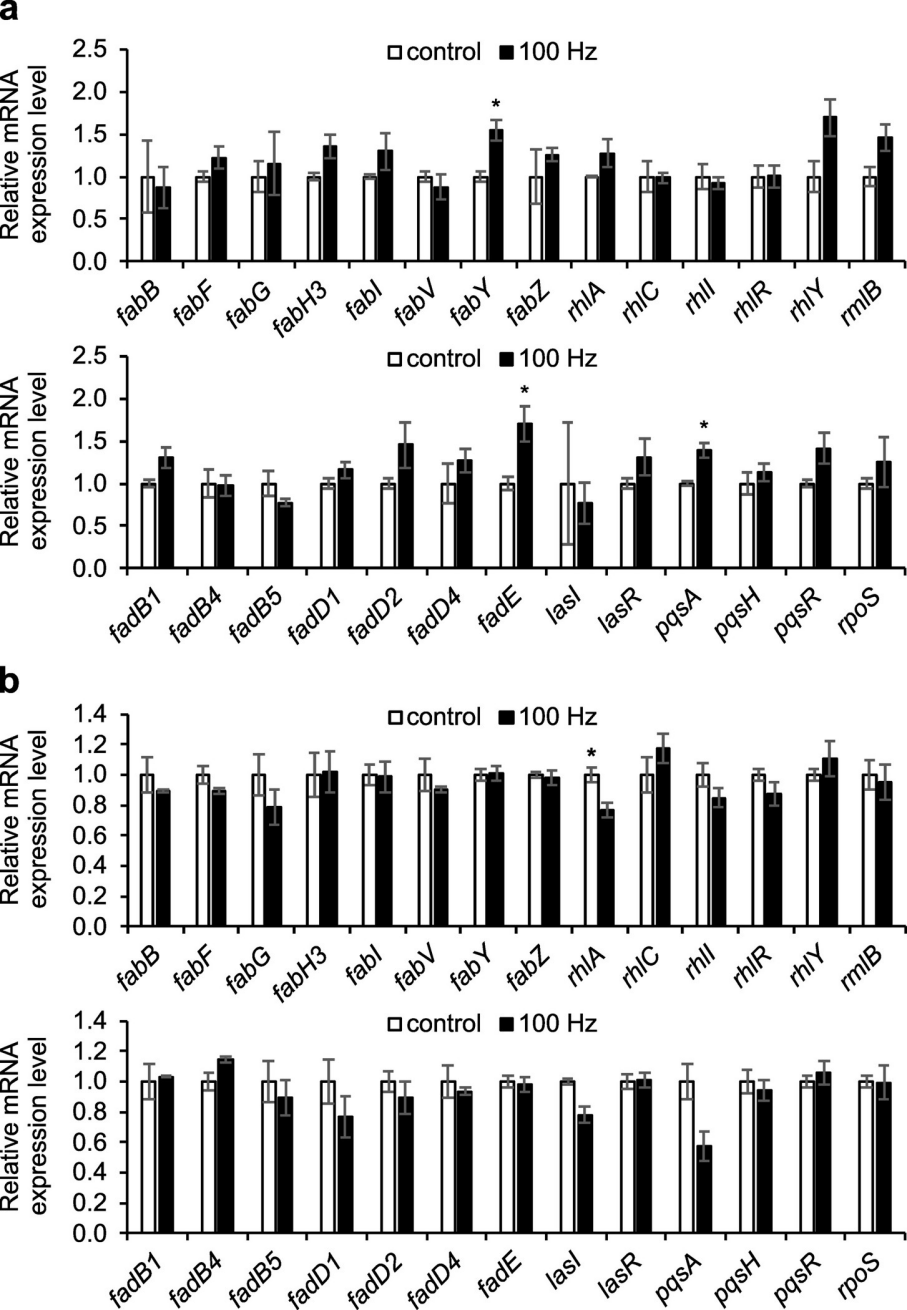
Relative transcript expression levels of genes involved in the biosynthesis of differential metabolites and related pathways in *Pseudomonas aeruginosa*. mRNA expression levels were quantitated in *Pseudomonas aeruginosa* at (a) 12 h and (b) 48 h after exposure to 100-Hz sound (100 Hz; black bars) and are indicated in the graph by normalizing the level of each gene with that of the control group without sound exposure (control; white bars). Quantitative real-time polymerase chain reaction was performed using the 2^–ΔΔCt^ method and standardized against the *rpoD* and *proC* transcript expression levels as internal controls. Data from three independent experiments are shown as the average ± standard errors of the mean. Student’s *t*-test: ^*^, *p* < 0.05.

**Fig 5:**
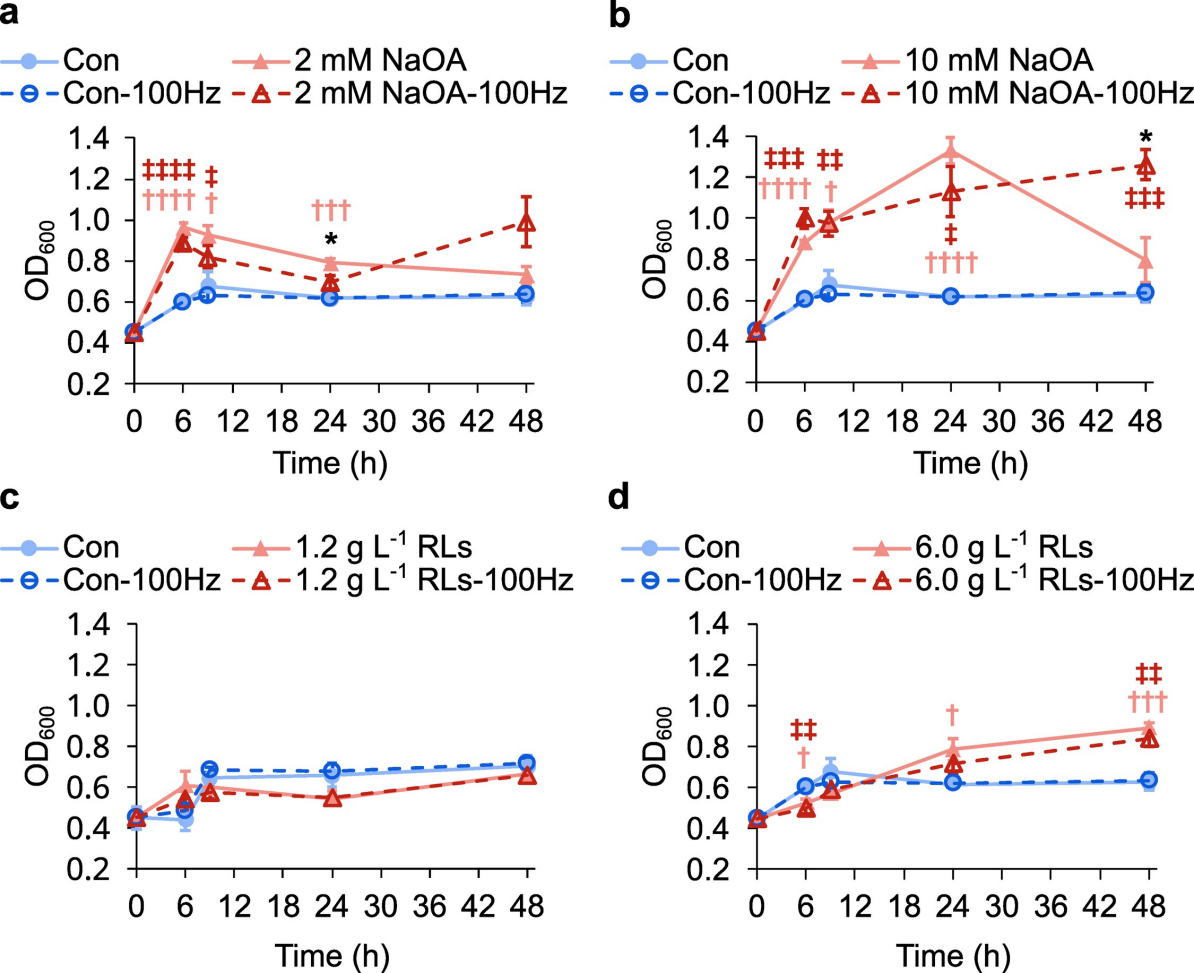
Acoustic vibration tolerance of *Pseudomonas aeruginosa* in the presence of response metabolites. Growth curves of *Pseudomonas aeruginosa* in the absence (control; Con; blue tone circle) or presence (red tone triangle) of (a) 2 mM sodium oleate (NaOA; 2 mM NaOA), (b) 10 mM NaOA, (c) 1.2 g L^–1^ rhamnolipids (RLs; 1.2 g L^–1^ RLs), or (d) 6.0 g L^–1^ RLs with (-100Hz; open marker with dashed line) or without (closed marker with solid line) stimulation by 100 Hz sound. One mL of cultures was collected at 6, 12, 24, or 48 h of incubation, mixed vigorously to obtain homogeneous culture, and subjected to optical density measurement at 600 nm (OD_600_). Data from three or four independent cultures are shown as the average ± standard errors of the mean. Student’s *t*-test for compound-treated samples with *vs*. without sound: ^*^, *p* < 0.05. Student’s *t*-test for compound-treated *vs*. Con samples without sound: ^†^, *p* < 0.05; ^††^, *p* < 0.01; ^†††^, *p* < 0.005; ^††††^, *p* < 0.001. Student’s *t*-test for compound-treated *vs*. Con samples with sound: ^‡^, *p* < 0.05; ^‡‡^, *p* < 0.01; ^‡‡‡^, *p* < 0.005; ^‡‡‡‡^, *p* < 0.001.

To our surprise, exposure to 100 Hz acoustic vibration for 48 h downregulated only one out of the 27 target genes, *rhlA*, with statistical significance and a small magnitude of change (1.3-fold), whereas the rest of the genes did not exhibit significant differences between the two groups (Fig 4B). Hypothesized that the observed changes in metabolite levels might be the consequence of earlier changes in gene transcript expression levels, we carried out similar qRT-PCR analyses at 12 h after sound exposure, when the *P. aeruginosa* cultures had just entered stationary phase (see Fig 5). Interestingly, the results revealed statistically significant upregulation of three genes: *fabY* (1.5-fold), *fadE* (1.7-fold), and *pqsA* (1.4-fold). However, the changes in gene expression remained relatively small in magnitude, and the relative levels of other genes, including *rhlA*, appeared unaltered (Fig 4A). Together, the findings on gene expression levels supported the findings of metabolite levels in the metabolomics data.

### Effects of oleic acid and rhamnolipids on the growth of *P. aeruginosa* under 100 Hz vibration

As the two main classes of metabolites with differential levels following 100 Hz sound exposure in extracellular samples were fatty acids and rhamnolipids (RLs), we evaluated the correlation between response and tolerance to acoustic vibration by testing the effects of these classes of metabolites on *P. aeruginosa* growth upon sound exposure by using sodium oleate (NaOA) as a representative of fatty acids, and a mixture of MRLs and DRLs for RLs. *P. aeruginosa* PAO1 was cultured under the same conditions as those for the metabolomics experiments but with or without supplementation of NaOA or RLs. However, because some cells were noticed to aggregate at the bottom of the cultured bottles at 24 h onwards, a behavior that might have consequences on bacterial growth, only 1 mL of PAO1 culture was grown in each culture vial, collected all at once at an indicated time point, and mixed vigorously to obtain homogeneous cultures before OD_600_ measurement. For the concentrations of treatment, it was reported that RLs blocked biofilm formation completely at a concentration of 250 *μ*M [46]. Rationalized that higher concentrations might yield more prominent changes, we treated the cells with higher concentrations of NaOA and RLs at 2 mM and 10 mM. Please note that for RLs, these concentrations are equivalent to 1.2 g L^–1^ and 6.0 g L^–1^ when the average molecular weight of the mixture is assumed to be 600 g mol^–1^.

Growth curves of *P. aeruginosa* cultures indicated that, in general, supplementation of NaOA into the culture resulted in significant increases in the cell densities of *P. aeruginosa* (Fig 5A and 5B). The increases were more prominent with a higher concentration of NaOA, with the cell densities continuing to increase to 24 h. At 24 h, NaOA-treated cultures grown in silence showed higher cell densities compared to those under 100-Hz vibration. However, the difference was only statistically significant with 2 mM NaOA cultures, and the magnitude of the difference was relatively small. At 48 h, the cell densities of silent NaOA cultures were found to decrease at both concentrations, whereas those of 100 Hz-exposed NaOA cultures appeared to increase slightly from previous time points, rendering their cell densities significantly different from their silent counterparts.

For RLs, no significant differences in growth were detected with 1.2 g L^–1^ RLs supplementation compared to untreated controls, with or without 100 Hz vibration treatment (Fig 5C). Nevertheless, with 6.0 g L^–1^ RLs supplementation, RLs slowed down the growth of *P. aeruginosa* with statistical significance at the beginning of incubation at 6 h, but the cell densities of the RLs cultures became higher than those of no-RLs controls at 24 h onward (Fig 5D). Unlike the cases of NaOA cultures, however, 100-Hz vibration did not alter the cell densities of RLs cultures compared to the no-vibration controls. Together, the data suggest that the elevation in levels of fatty acids, especially oleic acid, when exposed to 100-Hz acoustic vibration might play a role in maintaining the cell densities of *P. aeruginosa* at heightened levels for a longer period and support the correlation between the response and tolerance of *P. aeruginosa* to acoustic vibration.

## Discussion

Sound is an integral part of every environment, and its impact on living organisms has been the subject of extensive research. Studies have shown that sound, including music, can have positive effects on various living organisms, such as reducing stress, promoting embryonic development, and improving immune responses in humans, chicks, mice, and rats [47]. In plants, sound vibrations have been found to enhance crop yield, improve photosynthesis, increase tolerance to drought stress, influence hormone levels, and affect root growth and bending, as well as gene and protein expression related to defense and photosynthesis [48]. However, the effects of sound on bacteria, especially at the molecular level, have been limited, despite their significance in medical health and industrial applications. Therefore, in this study, we investigated the effects of 100 Hz acoustic vibration on *Pseudomonas aeruginosa* using untargeted metabolomics approaches to gain insights into the molecular-level impact of sound on bacteria.

In our study, *P. aeruginosa* was exposed to 100-Hz acoustic vibration in NB medium for 48 h, a condition previously reported to enhance biofilm formation by 0.3-fold compared to the silence control [13]. Interestingly, we did not observe any biofilm at all in our cases. The main differences between the two studies were the culture temperatures (30°C *vs*. 37°C) and the culture containers (culture glass bottle and vial *vs*. Petri dish). Previous research using the PAO1 strain of *P. aeruginosa* showed that biofilm formation was highest at 20°C, lowest at 25°C, and gradually increased again at higher temperatures, from 25°C to 30°C to 37°C [49]. The 30°C culture temperature employed in this study, which mimics the environmental condition, could thus reduce the amount of biofilm formation compared to the 37°C culture temperature in the previous study. Yet, in our view, the effects of temperature may be smaller compared to the effects of culture containers, as we could notice a small layer of biofilm at the air-liquid interface in all cultures when using a different type of culture tube in a separate but similar independent experiment.

As mentioned earlier, acoustic vibration has the potential to alter the growth of various microorganisms. However, a previous report on *P. aeruginosa* exposed to 800-Hz vibration showed increased bacterial cell numbers only in biofilm, but not in planktonic cells [13]. In our study, the growth of *P. aeruginosa* under 100-Hz sound treatment did not show significant differences compared to the silent control. As we observed only aggregates in our cultures, not biofilm, it is possible that *P. aeruginosa* cultures in our study may exhibit growth behaviors similar to planktonic cells, with unchanged cell numbers as previously described.

The untargeted metabolomics analysis of *P. aeruginosa* under 100-Hz sound treatment showed elevated levels of fatty acids, quinolones, and NAE, but depleted levels of RLs, compared to the silent control. RLs are a biosurfactant, which plays a crucial role in promoting biofilm formation in *P. aeruginosa*. In the early stages of biofilm development, a small amount of RLs increases the hydrophobicity of cells, facilitating their attachment to the surface. As the biofilm matures, a high concentration of RLs inhibits cell attachment and microcolony formation, maintaining the complex architecture of the biofilm and enabling the seeding dispersal of motile cells [50, 51]. Thus, reducing the levels of RLs under 100-Hz vibration exposure as detected here should enhance biofilm formation, which aligns with the effects found in the previous report [13]. Fatty acids (*i*.*e*., linoleic acid) and quinolones (*i*.*e*., synthetic fluoroquinolones) were demonstrated to promote biofilm dispersion as well [52, 53]. However, because their levels were increased upon sound exposure in this case, they would exert contradictory effects to those of decreasing RLs. It is therefore plausible that the observed increases in the levels of fatty acids and quinolones were simply artifacts resulting from the lower production of RLs, which left more of their precursors, *i*.*e*., free fatty acids, around and available for conversion into quinolones.

In fact, the qRT-PCR results (Fig 4) provide evidence for the correlation between the levels of RLs, fatty acids, and quinolones. The synthesis of RL involves the conversion of (*R*)-3-hydroxyacyl-CoA to 3-(3-hydroxyalkanoyloxy)alkanoic acid (HAA), the process catalyzed by RhlA. HAA then undergoes catalytic transformation by RhlB and RhlC to produce MRL and DRL, respectively (Fig 3) [29, 42, 54, 55]. Following exposure to 100-Hz vibration for 48 h, the level of *rhlA* gene was slightly but significantly reduced, compared to the unexposed control. This downregulation could potentially result in a decrease in the levels of RL products and an accumulation of (*R*)-3-hydroxyacyl-CoA and its precursors, thereby leading to the increase in the levels of fatty acids and quinolones. Notably, no changes in the level of *rhlA* gene were observed at the earlier time point of 12 h, indicating that the alteration in RL levels may occur only during the late stationary phase.

The effects of 100-Hz vibration exposure on gene expression were not limited to changes in the *rhlA* gene at 48 h, but also included significant upregulation of *fabY*, *fadE*, and *pqsA* genes at 12 h. FabY and FadE play critical roles in the *de novo* fatty acid synthesis (FAS II) and fatty acid *β*-oxidation pathways, respectively. FabY initiates the FAS II pathway by catalyzing the reaction between malonyl-ACP and acetyl-CoA to produce 3-ketoacyl-ACP [56], while FadE converts acyl-CoA to enoyl-CoA molecule, thereby starting off the *β*-oxidation pathway to break down long-chain fatty acids to those with shorter fatty acyl chains required for PQS and other quinolones syntheses [31]. In fact, previous studies have demonstrated that the PQS level was increased only in the presence of FadE when supplementing *P. aeruginosa* cultures with oleic acid (FA(18:1)), indicating that these two enzymes may contribute to the observed accumulation of fatty acids and quinolones upon vibration exposure. Additionally, PqsA catalyzes the conversion of anthranilic acid, a precursor in the biosynthesis of quinolones [21, 57]. The upregulation of *pqsA* gene expression may thus represent another factor directly contributing to the increased production of quinolones upon vibration exposure.

We also examined the impact of acoustic vibration on the regulator that is involved in environmental stress responses, biofilm formation, and virulence, as well as the QS networks that control RLs and quinolones. Specifically, we analyzed the levels of *rpoS* gene and the genes in these networks, such as lasI/R genes for the Las system, rhlI/R genes for the Rhl system, and pqsR gene for the PQS system [16, 44, 45]. Our results showed no significant changes in these transcript levels or in the product of LasI, 3-oxo-C_12_-HSL, indicating no association between the acoustic vibration, the regulator, and the Las and Rhl systems. However, we observed an increase in intracellular levels of quinolones other than PQS, suggesting the potential involvement of the PQS system in the response to the vibration. Nonetheless, additional mechanistic studies are necessary to elucidate the regulatory mechanisms behind these changes and their implications for the organism.

Upon exposure to 100-Hz vibration, another class of metabolites that shows an increase in intracellular levels is NAE. NAEs are naturally occurring molecules derived from fatty acids that act as signaling molecules in various physiological processes in animals and plants. They are involved in inflammation, metabolism, and stress response [58, 59]. The biosynthesis of NAEs can be initiated from phosphatidylethanolamine (PE), a phospholipid that is abundant in cell membranes. This involves the acylation of PE to *N*-acylphosphatidylethanolamine, which is then cleaved to form NAE. Alternatively, studies have described the enzymatic formation of NAE from fatty acids and ethanolamine [60], which is derived from PE through phosphodiesterases. Our study did not detect any change in the levels of PE under 100-Hz vibration treatment, implying that the availability of ethanolamine might be similar in both sound-treated and untreated conditions. Nevertheless, the elevated levels of fatty acids during sound exposure could potentially result in an increase in NAE levels, as supported by previous findings that have suggested a correlation between plasma NAE and fatty acid levels in women [61]. It should be noted, however, that there is currently no information available on the roles or biosynthesis of NAE in *P. aeruginosa*. Therefore, further investigation is needed to determine how changes in NAE levels affect *P. aeruginosa*.

In this study, two classes of metabolites, namely fatty acids and RLs, were observed to exhibit increased and decreased extracellular levels, respectively, upon exposure to 100-Hz acoustic vibration. From an evolutionary perspective, organisms may produce responses to protect against stress and enhance cell tolerance [1]. Accordingly, adding fatty acids to the culture media, which would mimic the observed change, may improve cell survival under sound exposure, while adding RL mixtures, which oppose the observed change, may sensitize cells. This hypothesis was supported by the growth of *P. aeruginosa* in sodium oleate under acoustic vibration treatment, which remained the only culture with heightened cell densities at 48 h. In general, the cell densities of *P. aeruginosa* in sodium oleate were also increased compared to the no-compound control, potentially serving as nutrients by entering through the *β*-oxidation pathway [30]. Interestingly, the lack of growth changes upon RL supplementation suggests that the response may be a general one, unrelated to *P. aeruginosa*’s survival and tolerance to sound, or the changes in RL levels may affect other physiological conditions not assessed in this study.

## Conclusions

In summary, we utilized untargeted metabolomics analysis to investigate the effects of 100-Hz acoustic vibration on the hydrophobic metabolites of *P. aeruginosa* PAO1, both intra- and extracellularly. Our analysis revealed alterations in the levels of 111 metabolite ions, of which 75% were assigned probable structures using accurate masses, retention time, tandem mass spectra, and some synthetic standards. Fatty acids and their derivatives, quinolones, and NAEs showed increased levels upon sound exposure, while RLs were decreased. qRT-PCR experiments indicated the slight downregulation of RL biosynthetic genes and upregulation of fatty acid and quinolone biosynthetic genes, but no significant changes in the regulator or QS network genes were observed. Furthermore, sodium oleate, a representative fatty acid, was found to enhance the tolerance of *P. aeruginosa* in the presence of acoustic stimulation at 48 h. For the first time, these findings shed light on the ways in which mechanical vibrations affect bacterial metabolism, physiology, and gene expression, with potential implications for controlling bacterial infections. Future work will aim to further elucidate the involved mechanisms, especially at the regulatory and mechanotransduction levels, apply the studies to clinical or other strains of *P. aeruginosa* under environments conducive to biofilm formation, and extend these methods to study other bacteria for medical, agricultural, and industrial applications.

## Supporting information

S1 File S1–S10 Tables and S1 Fig. S1–S7 Tables. Significantly increased or decreased positive-mode or negative-mode ions in 100-Hz-treated *P. aeruginosa* extracellular or intracellular samples compared to the no-sound control in the metabolomics analysis. S8 Table. Levels of other known metabolites in 100-Hz-treated *P. aeruginosa* extracellular and intracellular samples compared to the no-sound control in the metabolomics analysis. S9 Table. List of metabolite standards with MS/MS spectra in the positive and negative ion modes. S10 Table. Oligonucleotides used as forward and reverse primers for amplification of target genes in the quantitative real-time polymerase chain reaction. S1 Fig. List of MS/MS Spectra.
(PDF)
