# Supplementary material for: Metabolomics responses and tolerance of Pseudomonas aeruginosa under acoustic vibration stress
Source: PLoS One. 2024 Jan 29;19(1):e0297030. doi: 10.1371/journal.pone.0297030 (PMC10824448; doi:10.1371/journal.pone.0297030)
Supplement: S1 File — S1–S7 Tables. Significantly increased or decreased positive-mode or negative-mode ions in 100-Hz-treated P. aeruginosa extracellular or intracellular samples compared to the no-sound control in the metabolomics analysis. S8 Table. Levels of other known metabolites in 100-Hz-treated P. aeruginosa extracellular and intracellular samples compared to the no-sound control in the metabolomics analysis. S9 Table. List of metabolite standards with MS/MS spectra in the positive and negative ion modes. S10 Table. Oligonucleotides used as forward and reverse primers for amplification of target genes in the quantitative real-time polymerase chain reaction. S1 Fig. List of MS/MS Spectra. (PDF) [file pone.0297030.s001.pdf]

# Supporting Information

# Metabolomics Responses and Tolerance of *Pseudomonas aeruginosa* under Acoustic Vibration Stress

**Nawaporn Vinayavekhin[1,2,*], Thanyaporn Wattanophas[1], Mark Francis Murphy[3], Alisa S. Vangnai[2,4], Glyn Hobbs[3]**

[*]Corresponding author. E-mail: nawaporn.v@chula.ac.th (NV)

[1] Center of Excellence in Natural Products Chemistry, Department of Chemistry, Faculty of Science, Chulalongkorn University, Bangkok 10330, Thailand

[2] Center of Excellence in Biocatalyst and Sustainable Biotechnology, Faculty of Science, Chulalongkorn University, Bangkok 10330, Thailand

[3] School of Pharmacy and Biomolecular Sciences, Liverpool John Moores University, Liverpool L33AF, United Kingdom

[4] Department of Biochemistry, Faculty of Science, Chulalongkorn University, Bangkok 10330, Thailand

**List of Figures**

**S1 Fig. List of MS/MS Spectra (**as referred to in S1–S6 and S9 Tables). .......................................................S20

**List of Tables**

**S1 Table**. Significantly increased positive-mode ions in 100-Hz-treated *P. aeruginosa* extracellular samples compared to the no-sound control in the metabolomics analysis. Data are shown as the mass-to-charge ratio (m/z), retention time (RT), (a) potential identification and MS/MS spectrum, (b) integrated mass ion intensity (MSII), and (c) adjusted mass ion intensity (aMSII). The (b) MSII and (c) aMSII values are for the three *P. aeruginosa* cultures with (100Hz-1–3) or without (Con-1–3) 100-Hz sound treatments and their respective averages (100Hz-avg and Con-avg, respectively) for a set of metabolomics analysis. ....... S5

**S2 Table**. Significantly increased negative-mode ions in 100-Hz-treated *P. aeruginosa* extracellular samples compared to the no-sound control in the metabolomics analysis. Data are shown as the mass-to-charge ratio (m/z), retention time (RT), (a) potential identification and MS/MS spectrum, (b) integrated mass ion intensity (MSII), and (c) adjusted mass ion intensity (aMSII). The (b) MSII and (c) aMSII values are for the three *P. aeruginosa* cultures with (100Hz-1–3) or without (Con-1–3) 100-Hz sound treatments and their respective averages (100Hz-avg and Con-avg, respectively) for a set of metabolomics analysis. ....... S6

**S3 Table.** Significantly increased positive-mode ions in 100-Hz-treated *P. aeruginosa* intracellular samples compared to the no-sound control in the metabolomics analysis. Data are shown as the mass-to-charge ratio (m/z), retention time (RT), (a) potential identification and MS/MS spectrum, (b) integrated mass ion intensity (MSII), and (c) adjusted mass ion intensity (aMSII). The (b) MSII and (c) aMSII values are for the three *P. aeruginosa* cultures with (100Hz-1–3) or without (Con-1–3) 100-Hz sound treatments and their respective averages (100Hz-avg and Con-avg, respectively) for a set of metabolomics analysis. ....... S7

**S4 Table.** Significantly increased negative-mode ions in 100-Hz-treated *P. aeruginosa* intracellular samples compared to the no-sound control in the metabolomics analysis. Data are shown as the mass-to-charge ratio (m/z), retention time (RT), (a) potential identification and MS/MS spectrum, (b) integrated mass ion intensity (MSII), and (c) adjusted mass ion intensity (aMSII). The (b) MSII and (c) aMSII values are for the three *P. aeruginosa* cultures with (100Hz-1–3) or without (Con-1–3) 100-Hz sound treatments and their respective averages (100Hz-avg and Con-avg, respectively) for a set of metabolomics analysis. ....... S9

**S5 Table.** Significantly decreased positive-mode ions in 100-Hz-treated *P. aeruginosa* extracellular samples compared to the no-sound control in the metabolomics analysis. Data are shown as the mass-to-charge ratio (m/z), retention time (RT), (a) potential identification and MS/MS spectrum, (b) integrated mass ion intensity (MSII), and (c) adjusted mass ion intensity (aMSII). The (b) MSII and (c) aMSII values are for the three *P. aeruginosa* cultures with (100Hz-1–3) or without (Con-1–3) 100-Hz sound treatments and their respective averages (100Hz-avg and Con-avg, respectively) for a set of metabolomics analysis. ..... S12

**S6 Table.** Significantly decreased negative-mode ions in 100-Hz-treated *P. aeruginosa* extracellular samples compared to the no-sound control in the metabolomics analysis. Data are shown as the mass-to-charge ratio (m/z), retention time (RT), (a) potential identification and MS/MS spectrum, (b) integrated mass ion intensity (MSII), and (c) adjusted mass ion intensity (aMSII). The (b) MSII and (c) aMSII values are for the three *P. aeruginosa* cultures with (100Hz-1–3) or without (Con-1–3) 100-Hz sound treatments and their respective averages (100Hz-avg and Con-avg, respectively) for a set of metabolomics analysis. ..... S14

**S7 Table.** Significantly decreased positive-mode ions in 100-Hz-treated *P. aeruginosa* intracellular samples compared to the no-sound control in the metabolomics analysis. Data are shown as the mass-to-charge ratio (m/z), retention time (RT), (a) potential identification and MS/MS spectrum, (b) integrated mass ion intensity (MSII), and (c) adjusted mass ion intensity (aMSII). The (b) MSII and (c) aMSII values are for the three *P. aeruginosa* cultures with (100Hz-1–3) or without (Con-1–3) 100-Hz sound treatments and their respective averages (100Hz-avg and Con-avg, respectively) for a set of metabolomics analysis. ..... S17

**S8 Table.** Levels of other known metabolites in 100-Hz-treated *P. aeruginosa* extracellular and intracellular samples compared to the no-sound control in the metabolomics analysis. Data are shown as metabolite class and acyl chain, (a) integrated mass ion intensity (MSII), and (b) adjusted mass ion intensity (aMSII) of ions as indicated in the table in the manuscript. The (a) MSII and (b) aMSII values are for the three *P. aeruginosa* cultures with (100Hz-1–3) or without (Con-1–3) 100-Hz sound treatments and their respective averages (100Hz-avg and Con-avg, respectively) for a set of metabolomics analysis. .................... S18

**S9 Table.** List of metabolite standards with MS/MS spectra in the positive and negative ion modes .............. S19

**S10 Table.** Oligonucleotides used as forward and reverse primers for amplification of target genes in the quantitative real-time polymerase chain reaction with the controls being *rpoD* and *proC* genes...................... S69

**List of Abbreviations**

| | |
|---|---|
| 100Hz | *P. aeruginosa* samples grown with 100-Hz sound treatments |
| 3-oxo-$C_{12}$-HSL | *N*-(3-oxododecanoyl)-L-homoserine lactone |
| AHQ | 2-alkyl-4-hydroxyquinoline |
| aMSII | adjusted mass ion intensity |
| AQNO | 2-alkyl-4-hydroxyquinoline *N*-oxide |
| avg | average |
| Con | control (*P. aeruginosa* samples grown without sound treatments) |
| $C_n$-PQS | 2-alkyl-3-hydroxy-4-quinolone |
| DAG | diacylglycerol |
| DRL | dirhamnolipid |
| FA | fatty acid |
| HAA | 3-(3-hydroxyalkanoyloxy)alkanoic acid |
| HHQ | AHQ (7:0) |
| HNQ | AHQ (9:0) |
| HQNO | AQNO (7:0) |
| Intens. | intensity (MSII) |
| MRL | monorhamnolipid |
| MS | mass spectrum, mass spectrometry, or mass spectrometer |
| MSII | integrated mass ion intensity |
| m/z | mass-to-charge ratio |
| NAE | *N*-acylethanolamine |
| PE | phosphatidylethanolamine |
| PG | phosphatidylglycerol |
| RT | retention time |

**S1 Table**. Significantly increased positive-mode ions in 100-Hz-treated *P. aeruginosa* extracellular samples compared to the no-sound control in the metabolomics analysis. Data are shown as the mass-to-charge ratio (m/z), retention time (RT), (a) potential identification and MS/MS spectrum, (b) integrated mass ion intensity (MSII), and (c) adjusted mass ion intensity (aMSII). The (b) MSII and (c) aMSII values are for the three *P. aeruginosa* cultures with (100Hz-1–3) or without (Con-1–3) 100-Hz sound treatments and their respective averages (100Hz-avg and Con-avg, respectively) for a set of metabolomics analysis.

(a) Significantly increased positive-mode ions in 100-Hz-treated *P. aeruginosa* extracellular samples (potential identification and MS/MS spectrum)

| # | m/z | RT (min) | Ion | Potential identification | MS/MS spectrum |
|---|---|---|---|---|---|
| 1 | 299.2002 | 34.6 | ? | ? | - |
| 2 | 301.2157 | 35.8 | ? | ? | - |
| 3 | 631.4618 | 36.7 | ? | ? | - |

(b) Significantly increased positive-mode ions in 100-Hz-treated *P. aeruginosa* extracellular samples (MSII)

| # | m/z | RT (min) | Integrated mass ion intensity (MSII) | | | | | | | |
|---|---|---|---|---|---|---|---|---|---|---|
| | | | 100Hz-1 | 100Hz-2 | 100Hz-3 | 100Hz-avg | Con-1 | Con-2 | Con-3 | Con-avg |
| 1 | 299.2002 | 34.6 | 8.7E+05 | 1.0E+06 | 1.0E+06 | 9.7E+05 | 0.0E+00 | 0.0E+00 | 0.0E+00 | 0.0E+00 |
| 2 | 301.2157 | 35.8 | 5.2E+05 | 3.4E+05 | 3.7E+05 | 4.1E+05 | 1.8E+05 | 2.1E+05 | 1.7E+05 | 1.8E+05 |
| 3 | 631.4618 | 36.7 | 2.0E+05 | 1.7E+05 | 2.2E+05 | 2.0E+05 | 0.0E+00 | 1.2E+03 | 2.3E+03 | 1.2E+03 |

(c) Significantly increased positive-mode ions in 100-Hz-treated *P. aeruginosa* extracellular samples (aMSII)

| # | m/z | RT (min) | Adjusted integrated mass ion intensity (aMSII) | | | | | | | |
|---|---|---|---|---|---|---|---|---|---|---|
| | | | 100Hz-1 | 100Hz-2 | 100Hz-3 | 100Hz-avg | Con-1 | Con-2 | Con-3 | Con-avg |
| 1 | 299.2002 | 34.6 | 8.6E+05 | 1.0E+06 | 1.1E+06 | 9.8E+05 | 0.0E+00 | 0.0E+00 | 0.0E+00 | 0.0E+00 |
| 2 | 301.2157 | 35.8 | 5.1E+05 | 3.5E+05 | 3.7E+05 | 4.1E+05 | 1.8E+05 | 2.0E+05 | 1.6E+05 | 1.8E+05 |
| 3 | 631.4618 | 36.7 | 2.0E+05 | 1.8E+05 | 2.3E+05 | 2.0E+05 | 0.0E+00 | 1.1E+03 | 2.2E+03 | 1.1E+03 |

**S2 Table**. Significantly increased negative-mode ions in 100-Hz-treated *P. aeruginosa* extracellular samples compared to the no-sound control in the metabolomics analysis. Data are shown as the mass-to-charge ratio (m/z), retention time (RT), (a) potential identification and MS/MS spectrum, (b) integrated mass ion intensity (MSII), and (c) adjusted mass ion intensity (aMSII). The (b) MSII and (c) aMSII values are for the three *P. aeruginosa* cultures with (100Hz-1–3) or without (Con-1–3) 100-Hz sound treatments and their respective averages (100Hz-avg and Con-avg, respectively) for a set of metabolomics analysis.

(a) Significantly increased negative-mode ions in 100-Hz-treated *P. aeruginosa* extracellular samples (potential identification and MS/MS spectrum)

| # | m/z | RT (min) | Ion | Potential identification | MS/MS spectrum |
|---|---|---|---|---|---|
| 1 | 253.2198 | 19.4 | $[M-H]^-$ | FA (16:1) | - |
| 2 | 267.1972 | 17.4 | $[M-H]^-$ | oxo FA (16:1) | - |
| 3 | 281.2509 | 20.2 | $[M-H]^-$ | FA (18:1) | - |
| 4 | 335.2564 | 20.4 | ? | ? | - |
| 5 | 375.2719 | 18.1 | ? | ? | - |

(b) Significantly increased negative-mode ions in 100-Hz-treated *P. aeruginosa* extracellular samples (MSII)

| # | m/z | RT (min) | Integrated mass ion intensity (MSII) | | | | | | | |
|---|---|---|---|---|---|---|---|---|---|---|
| | | | 100Hz-1 | 100Hz-2 | 100Hz-3 | 100Hz-avg | Con-1 | Con-2 | Con-3 | Con-avg |
| 1 | 253.2198 | 19.4 | 1.2E+07 | 9.0E+06 | 8.5E+06 | 9.8E+06 | 4.6E+05 | 9.5E+05 | 8.4E+05 | 7.5E+05 |
| 2 | 267.1972 | 17.4 | 1.0E+05 | 6.3E+04 | 8.5E+04 | 8.4E+04 | 1.5E+04 | 3.7E+04 | 2.0E+04 | 2.4E+04 |
| 3 | 281.2509 | 20.2 | 1.7E+07 | 1.1E+07 | 1.5E+07 | 1.4E+07 | 2.9E+06 | 2.2E+04 | 1.9E+06 | 1.6E+06 |
| 4 | 335.2564 | 20.4 | 1.5E+04 | 7.5E+03 | 1.0E+04 | 1.1E+04 | 3.9E+03 | 3.5E+02 | 4.5E+03 | 2.9E+03 |
| 5 | 375.2719 | 18.1 | 1.6E+04 | 1.3E+04 | 2.2E+04 | 1.7E+04 | 4.8E+03 | 1.0E+04 | 8.5E+03 | 7.9E+03 |

(c) Significantly increased negative-mode ions in 100-Hz-treated *P. aeruginosa* extracellular samples (aMSII)

| # | m/z | RT (min) | Adjusted integrated mass ion intensity (MSII) | | | | | | | |
|---|---|---|---|---|---|---|---|---|---|---|
| | | | 100Hz-1 | 100Hz-2 | 100Hz-3 | 100Hz-avg | Con-1 | Con-2 | Con-3 | Con-avg |
| 1 | 253.2198 | 19.4 | 1.2E+07 | 9.3E+06 | 8.7E+06 | 9.9E+06 | 4.6E+05 | 9.3E+05 | 8.2E+05 | 7.4E+05 |
| 2 | 267.1972 | 17.4 | 1.0E+05 | 6.5E+04 | 8.6E+04 | 8.4E+04 | 1.5E+04 | 3.7E+04 | 1.9E+04 | 2.4E+04 |
| 3 | 281.2509 | 20.2 | 1.7E+07 | 1.1E+07 | 1.5E+07 | 1.4E+07 | 2.9E+06 | 2.2E+04 | 1.8E+06 | 1.6E+06 |
| 4 | 335.2564 | 20.4 | 1.5E+04 | 7.8E+03 | 1.0E+04 | 1.1E+04 | 3.9E+03 | 3.4E+02 | 4.4E+03 | 2.9E+03 |
| 5 | 375.2719 | 18.1 | 1.6E+04 | 1.4E+04 | 2.2E+04 | 1.7E+04 | 4.8E+03 | 1.0E+04 | 8.2E+03 | 7.7E+03 |

**S3 Table.** Significantly increased positive-mode ions in 100-Hz-treated *P. aeruginosa* intracellular samples compared to the no-sound control in the metabolomics analysis. Data are shown as the mass-to-charge ratio (m/z), retention time (RT), (a) potential identification and MS/MS spectrum, (b) integrated mass ion intensity (MSII), and (c) adjusted mass ion intensity (aMSII). The (b) MSII and (c) aMSII values are for the three *P. aeruginosa* cultures with (100Hz-1–3) or without (Con-1–3) 100-Hz sound treatments and their respective averages (100Hz-avg and Con-avg, respectively) for a set of metabolomics analysis.

(a) Significantly increased positive-mode ions in 100-Hz-treated *P. aeruginosa* intracellular samples (potential identification and MS/MS spectrum)

| # | m/z | RT (min) | Ion | Potential identification | MS/MS spectrum |
|---|---|---|---|---|---|
| 1 | 166.0873 | 36.4 | $[M + H]^+$ | Reduced hydroxy quinolone fragment (?) | - |
| 2 | 166.0869 | 35.7 | $[M + H]^+$ | Reduced hydroxy quinolone fragment (?) | - |
| 3 | 239.2338 | 32.9 | ? | ? | - |
| 4 | 263.2368 | 30.0 | ? | ? | - |
| 5 | 270.1867 | 27.1 | $[M + H]^+$ | AHQ (9:1) | S40 |
| 6 | 272.2028 | 27.2 | $[M + H]^+$ | AHQ (9:0) | S41 |
| 7 | 276.2302 | 32.2 | $[M + H]^+$ | Reduced AHQ (9:0) (4-hydroxy-2-nonyl-5,6,7,8-tetrahydroquinoline) | - |
| 8 | 279.2192 | 30.0 | ? | ? | - |
| 9 | 291.2243 | 35.7 | ? | ? | - |
| 10 | 301.2114 | 35.9 | ? | ? | - |
| 11 | 313.2138 | 35.7 | ? | ? | - |
| 12 | 325.2120 | 35.8 | ? | ? | - |

(b) Significantly increased positive-mode ions in 100-Hz-treated *P. aeruginosa* intracellular samples (MSII)

| # | m/z | RT (min) | Integrated mass ion intensity (MSII) | | | | | | | |
|---|---|---|---|---|---|---|---|---|---|---|
| | | | 100Hz-1 | 100Hz-2 | 100Hz-3 | 100Hz-avg | Con-1 | Con-2 | Con-3 | Con-avg |
| 1 | 166.0873 | 36.4 | 1.4E+05 | 9.6E+04 | 1.1E+05 | 1.1E+05 | 3.7E+04 | 4.5E+04 | 0.0E+00 | 2.7E+04 |
| 2 | 166.0869 | 35.7 | 1.1E+05 | 1.1E+05 | 1.1E+05 | 1.1E+05 | 4.3E+04 | 4.0E+04 | 1.1E+03 | 2.8E+04 |
| 3 | 239.2338 | 32.9 | 2.9E+04 | 2.0E+04 | 2.9E+04 | 2.6E+04 | 7.8E+03 | 6.8E+03 | 7.7E+03 | 7.4E+03 |
| 4 | 263.2368 | 30.0 | 6.5E+05 | 3.6E+05 | 5.9E+05 | 5.4E+05 | 9.6E+04 | 8.3E+04 | 6.0E+04 | 8.0E+04 |
| 5 | 270.1867 | 27.1 | 8.8E+06 | 4.6E+06 | 8.2E+06 | 7.2E+06 | 1.6E+06 | 1.7E+06 | 2.3E+06 | 1.9E+06 |
| 6 | 272.2028 | 27.2 | 1.8E+07 | 9.8E+06 | 1.6E+07 | 1.5E+07 | 3.8E+06 | 4.2E+06 | 5.3E+06 | 4.4E+06 |
| 7 | 276.2302 | 32.2 | 1.1E+05 | 9.5E+04 | 1.3E+05 | 1.1E+05 | 0.0E+00 | 3.1E+01 | 0.0E+00 | 1.0E+01 |
| 8 | 279.2192 | 30.0 | 5.0E+05 | 2.9E+05 | 2.7E+05 | 3.5E+05 | 9.6E+04 | 5.3E+04 | 1.8E+02 | 5.0E+04 |
| 9 | 291.2243 | 35.7 | 8.7E+04 | 6.7E+04 | 9.2E+04 | 8.2E+04 | 1.9E+04 | 1.9E+03 | 0.0E+00 | 7.0E+03 |
| 10 | 301.2114 | 35.9 | 1.2E+06 | 1.4E+06 | 1.8E+06 | 1.5E+06 | 5.7E+05 | 3.2E+05 | 2.2E+05 | 3.7E+05 |
| 11 | 313.2138 | 35.7 | 3.1E+05 | 2.4E+05 | 3.4E+05 | 3.0E+05 | 0.0E+00 | 0.0E+00 | 0.0E+00 | 0.0E+00 |
| 12 | 325.2120 | 35.8 | 1.0E+05 | 7.9E+04 | 1.1E+05 | 9.7E+04 | 1.3E+04 | 1.4E+04 | 1.1E+04 | 1.3E+04 |

(c) Significantly increased positive-mode ions in 100-Hz-treated *P. aeruginosa* intracellular samples (aMSII)

| # | m/z | RT (min) | Adjusted integrated mass ion intensity (aMSII) | | | | | | | |
|---|---|---|---|---|---|---|---|---|---|---|
| | | | 100Hz-1 | 100Hz-2 | 100Hz-3 | 100Hz-avg | Con-1 | Con-2 | Con-3 | Con-avg |
| 1 | 166.0873 | 36.4 | 1.3E+05 | 9.9E+04 | 1.2E+05 | 1.2E+05 | 3.7E+04 | 4.4E+04 | 0.0E+00 | 2.7E+04 |
| 2 | 166.0869 | 35.7 | 1.1E+05 | 1.1E+05 | 1.1E+05 | 1.1E+05 | 4.3E+04 | 3.9E+04 | 1.0E+03 | 2.8E+04 |
| 3 | 239.2338 | 32.9 | 2.9E+04 | 2.1E+04 | 2.9E+04 | 2.6E+04 | 7.9E+03 | 6.6E+03 | 7.5E+03 | 7.4E+03 |
| 4 | 263.2368 | 30.0 | 6.4E+05 | 3.8E+05 | 6.0E+05 | 5.4E+05 | 9.7E+04 | 8.1E+04 | 5.8E+04 | 7.9E+04 |
| 5 | 270.1867 | 27.1 | 8.7E+06 | 4.7E+06 | 8.4E+06 | 7.3E+06 | 1.6E+06 | 1.7E+06 | 2.2E+06 | 1.8E+06 |
| 6 | 272.2028 | 27.2 | 1.7E+07 | 1.0E+07 | 1.7E+07 | 1.5E+07 | 3.8E+06 | 4.1E+06 | 5.2E+06 | 4.4E+06 |
| 7 | 276.2302 | 32.2 | 1.1E+05 | 9.8E+04 | 1.3E+05 | 1.1E+05 | 0.0E+00 | 3.1E+01 | 0.0E+00 | 1.0E+01 |
| 8 | 279.2192 | 30.0 | 4.9E+05 | 3.0E+05 | 2.7E+05 | 3.5E+05 | 9.7E+04 | 5.2E+04 | 1.7E+02 | 5.0E+04 |
| 9 | 291.2243 | 35.7 | 8.6E+04 | 6.9E+04 | 9.4E+04 | 8.3E+04 | 1.9E+04 | 1.8E+03 | 0.0E+00 | 7.0E+03 |
| 10 | 301.2114 | 35.9 | 1.2E+06 | 1.5E+06 | 1.9E+06 | 1.5E+06 | 5.7E+05 | 3.1E+05 | 2.1E+05 | 3.7E+05 |
| 11 | 313.2138 | 35.7 | 3.0E+05 | 2.4E+05 | 3.5E+05 | 3.0E+05 | 0.0E+00 | 0.0E+00 | 0.0E+00 | 0.0E+00 |
| 12 | 325.2120 | 35.8 | 9.8E+04 | 8.2E+04 | 1.2E+05 | 9.9E+04 | 1.4E+04 | 1.4E+04 | 1.0E+04 | 1.3E+04 |

**S4 Table.** Significantly increased negative-mode ions in 100-Hz-treated *P. aeruginosa* intracellular samples compared to the no-sound control in the metabolomics analysis. Data are shown as the mass-to-charge ratio (m/z), retention time (RT), (a) potential identification and MS/MS spectrum, (b) integrated mass ion intensity (MSII), and (c) adjusted mass ion intensity (aMSII). The (b) MSII and (c) aMSII values are for the three *P. aeruginosa* cultures with (100Hz-1–3) or without (Con-1–3) 100-Hz sound treatments and their respective averages (100Hz-avg and Con-avg, respectively) for a set of metabolomics analysis.

(a) Significantly increased negative-mode ions in 100-Hz-treated *P. aeruginosa* intracellular samples (potential identification and MS/MS spectrum)

| # | m/z | RT (min) | Ion | Potential identification | MS/MS spectrum |
|---|---|---|---|---|---|
| 1 | 227.2013 | 19.0 | $[M-H]^-$ | FA (14:0) | - |
| 2 | 241.1795 | 15.2 | $[M-H]^-$ | Hydroxy FA (14:1) | - |
| 3 | 251.2017 | 19.4 | $[M-H]^-$ | FA (16:2) | - |
| 4 | 255.2336 | 19.7 | $[M-H]^-$ | FA (16:0) | - |
| 5 | 258.1484 | 13.7 | $[M-H]^-$ | AQNO (7:0) | S42 |
| 6 | 267.2336 | 19.6 | $[M-H]^-$ | FA (17:1) | - |
| 7 | 268.1687 | 27.5 | $[M-H]^-$ | AHQ (9:1) | S43 |
| 8 | 269.2107 | 17.0 | $[M-H]^-$ | Hydroxy FA (16:1) | S44 |
| 9 | 269.2119 | 19.0 | $[M-H]^-$ | Oxo FA (16:0) | - |
| 10 | 271.2271 | 19.4 | ? | ? | - |
| 11 | 279.2318 | 19.8 | $[M-H]^-$ | FA (18:2) | - |
| 12 | 281.2507 | 20.1 | $[M-H]^-$ | FA (18:1) | - |
| 13 | 286.1785 | 29.9 | $[M-H]^-$ | $C_n$-PQS (9:0) | S45 |
| 14 | 295.2272 | 19.0 | $[M-H]^-$ | Oxo FA (18:1) | - |
| 15 | 295.2653 | 20.3 | $[M-H]^-$ | FA (19:1) | - |
| 16 | 297.1534 | 19.2 | ? | ? | - |
| 17 | 297.2451 | 18.0 | $[M-H]^-$ | Hydroxy FA (18:1) | S46 |
| 18 | 299.2582 | 18.7 | $[M-H]^-$ | Hydroxy FA (18:0) | - |
| 19 | 303.2280 | 20.0 | $[M-H]^-$ | FA (20:4) | - |
| 20 | 304.1442 | 28.0 | ? | ? | - |
| 21 | 305.2430 | 20.2 | $[M-H]^-$ | FA (20:3) | - |
| 22 | 310.2700 | 36.3 | $[M-H]^-$ | *N*-acylethanolamine (17:1) | - |
| 23 | 312.1961 | 18.1 | $[M-H]^-$ | AQNO (11:1) | S47 |
| 24 | 319.2232 | 19.4 | $[M-H]^-$ | Oxo FA (20:3) | - |
| 25 | 319.2243 | 18.0 | $[M-H]^-$ | Hydroxy FA (20:4) | S48 |
| 26 | 322.2713 | 36.0 | $[M-H]^-$ | *N*-acylethanolamine (18:2) | - |
| 27 | 324.2870 | 37.6 | $[M-H]^-$ | *N*-acylethanolamine (18:1) | S49 |
| 28 | 335.2166 | 16.9 | ? | ? | - |
| 29 | 335.2513 | 20.3 | ? | ? | - |
| 30 | 338.3017 | 38.1 | $[M-H]^-$ | *N*-acylethanolamine (19:1) | S50 |
| 31 | 351.3325 | 41.6 | ? | ? | - |
| 32 | 354.2983 | 27.7 | $[M-H]^-$ | *N*-acyl valine (16:0) | S51 |
| 33 | 359.2162 | 18.0 | ? | ? | - |
| 34 | 380.3133 | 28.3 | $[M-H]^-$ | *N*-acyl valine (18:1) | S52 |
| 35 | 380.3147 | 20.6 | $[M-H]^-$ | *N*-acyl leucine (17:1) (?) | S53 |
| 36 | 412.2864 | 28.1 | ? | ? | - |
| 37 | 593.3007 | 18.8 | ? | ? | - |

(b) Significantly increased negative-mode ions in 100-Hz-treated *P. aeruginosa* intracellular samples (MSII)

| # | m/z | RT (min) | Integrated mass ion intensity (MSII) | | | | | | | |
|---|---|---|---|---|---|---|---|---|---|---|
| | | | 100Hz-1 | 100Hz-2 | 100Hz-3 | 100Hz-avg | Con-1 | Con-2 | Con-3 | Con-avg |
| 1 | 227.2013 | 19.0 | 1.6E+05 | 1.5E+05 | 1.9E+05 | 1.7E+05 | 9.0E+04 | 8.3E+04 | 6.2E+04 | 7.8E+04 |
| 2 | 241.1795 | 15.2 | 1.6E+04 | 1.4E+04 | 1.3E+04 | 1.4E+04 | 2.3E+03 | 3.3E+03 | 3.1E+03 | 2.9E+03 |
| 3 | 251.2017 | 19.4 | 1.3E+04 | 8.5E+03 | 1.4E+04 | 1.2E+04 | 2.5E+03 | 3.5E+03 | 5.0E+03 | 3.6E+03 |
| 4 | 255.2336 | 19.7 | 1.1E+07 | 1.2E+07 | 1.4E+07 | 1.2E+07 | 4.8E+06 | 4.0E+06 | 2.3E+06 | 3.7E+06 |
| 5 | 258.1484 | 13.7 | 1.6E+04 | 1.1E+04 | 1.7E+04 | 1.4E+04 | 4.8E+03 | 5.7E+03 | 8.7E+03 | 6.4E+03 |
| 6 | 267.2336 | 19.6 | 2.9E+06 | 2.1E+06 | 3.0E+06 | 2.7E+06 | 2.4E+05 | 3.5E+05 | 2.6E+05 | 2.8E+05 |
| 7 | 268.1687 | 27.5 | 2.3E+06 | 1.3E+06 | 1.9E+06 | 1.8E+06 | 5.2E+05 | 4.7E+05 | 6.1E+05 | 5.3E+05 |
| 8 | 269.2107 | 17.0 | 8.1E+04 | 6.5E+04 | 9.2E+04 | 8.0E+04 | 2.7E+04 | 2.8E+04 | 2.5E+04 | 2.7E+04 |
| 9 | 269.2119 | 19.0 | 5.3E+04 | 5.2E+04 | 4.7E+04 | 5.1E+04 | 0.0E+00 | 0.0E+00 | 0.0E+00 | 0.0E+00 |
| 10 | 271.2271 | 19.4 | 2.4E+05 | 2.3E+05 | 2.5E+05 | 2.4E+05 | 1.1E+05 | 9.8E+04 | 9.2E+04 | 9.9E+04 |
| 11 | 279.2318 | 19.8 | 4.8E+05 | 3.6E+05 | 6.3E+05 | 4.9E+05 | 5.8E+04 | 1.1E+05 | 7.7E+04 | 8.1E+04 |
| 12 | 281.2507 | 20.1 | 1.9E+07 | 1.5E+07 | 2.1E+07 | 1.8E+07 | 8.0E+05 | 4.8E+06 | 2.9E+06 | 2.8E+06 |
| 13 | 286.1785 | 29.9 | 3.0E+05 | 2.4E+05 | 3.7E+05 | 3.1E+05 | 1.4E+05 | 1.6E+05 | 1.4E+05 | 1.5E+05 |
| 14 | 295.2272 | 19.0 | 3.0E+05 | 1.9E+05 | 2.1E+05 | 2.3E+05 | 8.4E+04 | 9.5E+04 | 7.0E+04 | 8.3E+04 |
| 15 | 295.2653 | 20.3 | 6.5E+06 | 5.3E+06 | 7.5E+06 | 6.4E+06 | 7.6E+04 | 1.5E+06 | 1.2E+06 | 9.1E+05 |
| 16 | 297.1534 | 19.2 | 2.1E+06 | 1.6E+06 | 2.4E+06 | 2.0E+06 | 5.7E+05 | 6.5E+05 | 5.6E+05 | 6.0E+05 |
| 17 | 297.2451 | 18.0 | 4.0E+06 | 2.2E+06 | 4.1E+06 | 3.4E+06 | 4.2E+05 | 4.6E+05 | 3.0E+05 | 4.0E+05 |
| 18 | 299.2582 | 18.7 | 2.1E+05 | 1.9E+05 | 2.2E+05 | 2.0E+05 | 7.9E+04 | 7.8E+04 | 7.2E+04 | 7.6E+04 |
| 19 | 303.2280 | 20.0 | 3.0E+04 | 2.7E+04 | 3.2E+04 | 3.0E+04 | 2.6E+03 | 8.0E+03 | 6.5E+03 | 5.7E+03 |
| 20 | 304.1442 | 28.0 | 2.2E+04 | 1.3E+04 | 2.2E+04 | 1.9E+04 | 4.8E+03 | 2.8E+03 | 4.3E+03 | 4.0E+03 |
| 21 | 305.2430 | 20.2 | 1.3E+04 | 1.3E+04 | 1.7E+04 | 1.4E+04 | 9.8E+02 | 4.3E+03 | 4.3E+03 | 3.2E+03 |
| 22 | 310.2700 | 36.3 | 3.6E+04 | 2.6E+04 | 3.5E+04 | 3.2E+04 | 7.9E+03 | 1.1E+04 | 1.4E+04 | 1.1E+04 |
| 23 | 312.1961 | 18.1 | 1.7E+05 | 1.4E+05 | 2.2E+05 | 1.8E+05 | 4.3E+04 | 5.5E+04 | 9.4E+04 | 6.4E+04 |
| 24 | 319.2232 | 19.4 | 2.2E+04 | 1.3E+04 | 2.0E+04 | 1.8E+04 | 4.9E+03 | 4.9E+03 | 4.3E+03 | 4.7E+03 |
| 25 | 319.2243 | 18.0 | 7.8E+04 | 5.1E+04 | 8.1E+04 | 7.0E+04 | 1.6E+04 | 1.7E+04 | 1.3E+04 | 1.5E+04 |
| 26 | 322.2713 | 36.0 | 4.4E+04 | 4.3E+04 | 3.6E+04 | 4.1E+04 | 1.2E+04 | 1.3E+04 | 1.1E+04 | 1.2E+04 |
| 27 | 324.2870 | 37.6 | 5.4E+05 | 4.0E+05 | 6.3E+05 | 5.2E+05 | 1.4E+05 | 1.9E+05 | 1.8E+05 | 1.7E+05 |
| 28 | 335.2166 | 16.9 | 2.5E+04 | 1.8E+04 | 1.7E+04 | 2.0E+04 | 8.9E+03 | 9.4E+03 | 7.4E+03 | 8.6E+03 |
| 29 | 335.2513 | 20.3 | 1.2E+04 | 1.1E+04 | 1.4E+04 | 1.2E+04 | 1.3E+03 | 3.5E+03 | 3.2E+03 | 2.7E+03 |
| 30 | 338.3017 | 38.1 | 6.0E+04 | 4.5E+04 | 7.1E+04 | 5.9E+04 | 1.3E+04 | 2.2E+04 | 2.5E+04 | 2.0E+04 |
| 31 | 351.3325 | 41.6 | 1.6E+05 | 1.3E+05 | 1.3E+05 | 1.4E+05 | 5.7E+04 | 6.3E+04 | 7.3E+04 | 6.4E+04 |
| 32 | 354.2983 | 27.7 | 5.8E+04 | 4.4E+04 | 6.5E+04 | 5.6E+04 | 3.2E+04 | 2.3E+04 | 2.7E+04 | 2.8E+04 |
| 33 | 359.2162 | 18.0 | 2.2E+04 | 1.6E+04 | 2.4E+04 | 2.1E+04 | 4.7E+03 | 5.4E+03 | 3.8E+03 | 4.6E+03 |
| 34 | 380.3133 | 28.3 | 1.4E+05 | 1.1E+05 | 1.3E+05 | 1.2E+05 | 6.0E+04 | 5.0E+04 | 5.9E+04 | 5.6E+04 |
| 35 | 380.3147 | 20.6 | 1.4E+05 | 1.1E+05 | 1.9E+05 | 1.5E+05 | 9.9E+03 | 7.5E+04 | 6.4E+04 | 5.0E+04 |
| 36 | 412.2864 | 28.1 | 4.5E+04 | 3.9E+04 | 4.7E+04 | 4.4E+04 | 2.1E+04 | 1.7E+04 | 1.9E+04 | 1.9E+04 |
| 37 | 593.3007 | 18.8 | 2.0E+04 | 2.1E+04 | 2.2E+04 | 2.1E+04 | 1.2E+02 | 4.0E+01 | 0.0E+00 | 5.2E+01 |

(c) Significantly increased negative-mode ions in 100-Hz-treated *P. aeruginosa* intracellular samples (aMSII)

| # | m/z | RT (min) | Adjusted integrated mass ion intensity (aMSII) | | | | | | | |
|---|---|---|---|---|---|---|---|---|---|---|
| | | | 100Hz-1 | 100Hz-2 | 100Hz-3 | 100Hz-avg | Con-1 | Con-2 | Con-3 | Con-avg |
| 1 | 227.2013 | 19.0 | 1.5E+05 | 1.6E+05 | 1.9E+05 | 1.7E+05 | 9.1E+04 | 8.2E+04 | 6.0E+04 | 7.8E+04 |
| 2 | 241.1795 | 15.2 | 1.6E+04 | 1.5E+04 | 1.3E+04 | 1.4E+04 | 2.3E+03 | 3.2E+03 | 3.1E+03 | 2.9E+03 |
| 3 | 251.2017 | 19.4 | 1.2E+04 | 8.8E+03 | 1.5E+04 | 1.2E+04 | 2.5E+03 | 3.4E+03 | 4.8E+03 | 3.6E+03 |
| 4 | 255.2336 | 19.7 | 1.1E+07 | 1.2E+07 | 1.5E+07 | 1.3E+07 | 4.9E+06 | 3.9E+06 | 2.2E+06 | 3.7E+06 |
| 5 | 258.1484 | 13.7 | 1.6E+04 | 1.1E+04 | 1.7E+04 | 1.5E+04 | 4.9E+03 | 5.6E+03 | 8.4E+03 | 6.3E+03 |
| 6 | 267.2336 | 19.6 | 2.8E+06 | 2.2E+06 | 3.1E+06 | 2.7E+06 | 2.4E+05 | 3.5E+05 | 2.5E+05 | 2.8E+05 |
| 7 | 268.1687 | 27.5 | 2.3E+06 | 1.3E+06 | 1.9E+06 | 1.8E+06 | 5.2E+05 | 4.6E+05 | 6.0E+05 | 5.3E+05 |
| 8 | 269.2107 | 17.0 | 8.0E+04 | 6.7E+04 | 9.4E+04 | 8.0E+04 | 2.7E+04 | 2.8E+04 | 2.4E+04 | 2.6E+04 |
| 9 | 269.2119 | 19.0 | 5.2E+04 | 5.4E+04 | 4.8E+04 | 5.1E+04 | 0.0E+00 | 0.0E+00 | 0.0E+00 | 0.0E+00 |
| 10 | 271.2271 | 19.4 | 2.4E+05 | 2.4E+05 | 2.6E+05 | 2.5E+05 | 1.1E+05 | 9.6E+04 | 8.9E+04 | 9.8E+04 |
| 11 | 279.2318 | 19.8 | 4.7E+05 | 3.7E+05 | 6.4E+05 | 4.9E+05 | 5.8E+04 | 1.0E+05 | 7.5E+04 | 7.9E+04 |
| 12 | 281.2507 | 20.1 | 1.9E+07 | 1.5E+07 | 2.2E+07 | 1.9E+07 | 8.1E+05 | 4.7E+06 | 2.8E+06 | 2.8E+06 |
| 13 | 286.1785 | 29.9 | 3.0E+05 | 2.5E+05 | 3.8E+05 | 3.1E+05 | 1.4E+05 | 1.5E+05 | 1.4E+05 | 1.5E+05 |
| 14 | 295.2272 | 19.0 | 2.9E+05 | 2.0E+05 | 2.1E+05 | 2.3E+05 | 8.4E+04 | 9.3E+04 | 6.9E+04 | 8.2E+04 |
| 15 | 295.2653 | 20.3 | 6.4E+06 | 5.5E+06 | 7.7E+06 | 6.5E+06 | 7.7E+04 | 1.4E+06 | 1.2E+06 | 8.9E+05 |
| 16 | 297.1534 | 19.2 | 2.1E+06 | 1.6E+06 | 2.5E+06 | 2.1E+06 | 5.8E+05 | 6.4E+05 | 5.5E+05 | 5.9E+05 |
| 17 | 297.2451 | 18.0 | 3.9E+06 | 2.2E+06 | 4.2E+06 | 3.4E+06 | 4.3E+05 | 4.6E+05 | 2.9E+05 | 3.9E+05 |
| 18 | 299.2582 | 18.7 | 2.0E+05 | 2.0E+05 | 2.2E+05 | 2.1E+05 | 8.0E+04 | 7.7E+04 | 7.0E+04 | 7.6E+04 |
| 19 | 303.2280 | 20.0 | 3.0E+04 | 2.8E+04 | 3.3E+04 | 3.0E+04 | 2.6E+03 | 7.8E+03 | 6.4E+03 | 5.6E+03 |
| 20 | 304.1442 | 28.0 | 2.2E+04 | 1.3E+04 | 2.2E+04 | 1.9E+04 | 4.8E+03 | 2.8E+03 | 4.2E+03 | 3.9E+03 |
| 21 | 305.2430 | 20.2 | 1.3E+04 | 1.3E+04 | 1.7E+04 | 1.4E+04 | 1.0E+03 | 4.2E+03 | 4.2E+03 | 3.1E+03 |
| 22 | 310.2700 | 36.3 | 3.5E+04 | 2.7E+04 | 3.6E+04 | 3.3E+04 | 8.0E+03 | 1.1E+04 | 1.4E+04 | 1.1E+04 |
| 23 | 312.1961 | 18.1 | 1.7E+05 | 1.4E+05 | 2.2E+05 | 1.8E+05 | 4.3E+04 | 5.4E+04 | 9.1E+04 | 6.3E+04 |
| 24 | 319.2232 | 19.4 | 2.1E+04 | 1.4E+04 | 2.1E+04 | 1.9E+04 | 5.0E+03 | 4.8E+03 | 4.2E+03 | 4.7E+03 |
| 25 | 319.2243 | 18.0 | 7.7E+04 | 5.3E+04 | 8.3E+04 | 7.1E+04 | 1.6E+04 | 1.6E+04 | 1.2E+04 | 1.5E+04 |
| 26 | 322.2713 | 36.0 | 4.4E+04 | 4.4E+04 | 3.7E+04 | 4.1E+04 | 1.3E+04 | 1.3E+04 | 1.1E+04 | 1.2E+04 |
| 27 | 324.2870 | 37.6 | 5.3E+05 | 4.1E+05 | 6.4E+05 | 5.3E+05 | 1.4E+05 | 1.8E+05 | 1.8E+05 | 1.7E+05 |
| 28 | 335.2166 | 16.9 | 2.5E+04 | 1.9E+04 | 1.7E+04 | 2.0E+04 | 9.0E+03 | 9.2E+03 | 7.2E+03 | 8.5E+03 |
| 29 | 335.2513 | 20.3 | 1.2E+04 | 1.1E+04 | 1.5E+04 | 1.3E+04 | 1.3E+03 | 3.4E+03 | 3.1E+03 | 2.6E+03 |
| 30 | 338.3017 | 38.1 | 5.9E+04 | 4.6E+04 | 7.3E+04 | 5.9E+04 | 1.3E+04 | 2.2E+04 | 2.4E+04 | 2.0E+04 |
| 31 | 351.3325 | 41.6 | 1.6E+05 | 1.3E+05 | 1.4E+05 | 1.4E+05 | 5.7E+04 | 6.2E+04 | 7.0E+04 | 6.3E+04 |
| 32 | 354.2983 | 27.7 | 5.7E+04 | 4.5E+04 | 6.6E+04 | 5.6E+04 | 3.3E+04 | 2.3E+04 | 2.7E+04 | 2.7E+04 |
| 33 | 359.2162 | 18.0 | 2.2E+04 | 1.7E+04 | 2.5E+04 | 2.1E+04 | 4.7E+03 | 5.3E+03 | 3.7E+03 | 4.6E+03 |
| 34 | 380.3133 | 28.3 | 1.3E+05 | 1.1E+05 | 1.3E+05 | 1.3E+05 | 6.0E+04 | 4.9E+04 | 5.7E+04 | 5.5E+04 |
| 35 | 380.3147 | 20.6 | 1.4E+05 | 1.1E+05 | 2.0E+05 | 1.5E+05 | 1.0E+04 | 7.3E+04 | 6.2E+04 | 4.9E+04 |
| 36 | 412.2864 | 28.1 | 4.5E+04 | 4.0E+04 | 4.8E+04 | 4.4E+04 | 2.1E+04 | 1.6E+04 | 1.9E+04 | 1.9E+04 |
| 37 | 593.3007 | 18.8 | 2.0E+04 | 2.2E+04 | 2.2E+04 | 2.1E+04 | 1.2E+02 | 3.9E+01 | 0.0E+00 | 5.2E+01 |

**S5 Table.** Significantly decreased positive-mode ions in 100-Hz-treated *P. aeruginosa* extracellular samples compared to the no-sound control in the metabolomics analysis. Data are shown as the mass-to-charge ratio (m/z), retention time (RT), (a) potential identification and MS/MS spectrum, (b) integrated mass ion intensity (MSII), and (c) adjusted mass ion intensity (aMSII). The (b) MSII and (c) aMSII values are for the three *P. aeruginosa* cultures with (100Hz-1–3) or without (Con-1–3) 100-Hz sound treatments and their respective averages (100Hz-avg and Con-avg, respectively) for a set of metabolomics analysis.

(a) Significantly decreased positive-mode ions in 100-Hz-treated *P. aeruginosa* extracellular samples (potential identification and MS/MS spectrum)

| # | m/z | RT (min) | Ion | Potential identification | MS/MS spectrum |
|---|---|---|---|---|---|
| 1 | 153.1271 | 32.6 | $[M + H]^+$ | Acylium ion of hydroxy FA (10:0) – $H_2O$ fragment of MRL (10:0/10:0) | - |
| 2 | 308.2941 | 26.0 | ? | ? | - |
| 3 | 332.2222 | 28.8 | ? | Fragment of MRL (8:0/10:0) | - |
| 4 | 341.2666 | 31.3 | $[M + H]^+$ | MRL (10:0/10:0) – rhamnose fragment | - |
| 5 | 353.2296 | 30.2 | $[M + Na]^+$ | HAA fragment of MRL (8:0/10:0) | - |
| 6 | 385.2936 | 32.8 | $[M + H]^+$ | HAA fragment of MRL (10:0/12:1) | - |
| 7 | 387.3100 | 33.3 | $[M + H]^+$ | HAA fragment of MRL (10:0/12:0) | - |
| 8 | 499.2879 | 29.0 | $[M + Na]^+$ | MRL (8:0/10:0) | S54 |
| 9 | 505.3364 | 31.3 | $[M + H]^+$ | MRL (10:0/10:0) | - |
| 10 | 527.3188 | 31.3 | $[M + Na]^+$ | MRL (10:0/10:0) | S55 |
| 11 | 543.2922 | 31.3 | $[M + K]^+$ | MRL (10:0/10:0) | - |
| 12 | 548.3781 | 32.8 | $[M + NH_4]^+$ | MRL (10:0/12:1) | S56 |
| 13 | 549.3002 | 31.2 | $[M + 2Na – H]^+$ | MRL (10:0/10:0) | - |
| 14 | 550.3937 | 33.2 | $[M + NH_4]^+$ | MRL (10:0/12:0) | S57 |
| 15 | 553.3331 | 32.8 | $[M + Na]^+$ | MRL (10:0/12:1) | S58 |
| 16 | 555.3493 | 33.2 | $[M + Na]^+$ | MRL (10:0/12:0) | S59 |
| 17 | 653.4370 | 26.6 | ? | ? | - |
| 18 | 667.4531 | 27.0 | ? | ? | - |
| 19 | 668.4206 | 30.4 | $[M + NH_4]^+$ | DRL (10:0/10:0) | S60 |
| 20 | 696.4523 | 32.4 | $[M + NH_4]^+$ | DRL (10:0/12:0) | S61 |
| 21 | 755.4519 | 30.3 | ? | Adduct ion of MRL (8:0/10:0) (?) | - |
| 22 | 915.5195 | 32.8 | $[M + H]^+$ | HAA (10:0/12:1) fragment + MRL (10:0/12:1) | - |
| 23 | 1,053.6279 | 31.3 | $[2M + 2Na – H]^+$ | MRL (10:0/10:0) | - |
| 24 | 1,105.6331 | 32.7 | $[2M + 2Na – H]^+$ | MRL (10:0/12:1) | - |

(b) Significantly decreased positive-mode ions in 100-Hz-treated *P. aeruginosa* extracellular samples (MSII)

| # | m/z | RT (min) | Integrated mass ion intensity (MSII) | | | | | | | |
|---|---|---|---|---|---|---|---|---|---|---|
| | | | 100Hz-1 | 100Hz-2 | 100Hz-3 | 100Hz-avg | Con-1 | Con-2 | Con-3 | Con-avg |
| 1 | 153.1271 | 32.6 | 1.1E+04 | 8.9E+03 | 7.7E+03 | 9.1E+03 | 1.9E+05 | 2.2E+05 | 1.9E+05 | 2.0E+05 |
| 2 | 308.2941 | 26.0 | 0.0E+00 | 3.9E+04 | 1.7E+04 | 1.9E+04 | 1.1E+05 | 1.2E+05 | 1.2E+05 | 1.2E+05 |
| 3 | 332.2222 | 28.8 | 5.4E+04 | 5.2E+04 | 4.6E+04 | 5.0E+04 | 1.7E+05 | 1.3E+05 | 1.2E+05 | 1.4E+05 |
| 4 | 341.2666 | 31.3 | 0.0E+00 | 0.0E+00 | 0.0E+00 | 0.0E+00 | 1.8E+05 | 1.3E+05 | 1.7E+05 | 1.6E+05 |
| 5 | 353.2296 | 30.2 | 0.0E+00 | 0.0E+00 | 2.9E+04 | 9.5E+03 | 1.4E+06 | 9.7E+05 | 1.1E+06 | 1.2E+06 |
| 6 | 385.2936 | 32.8 | 6.1E+04 | 4.4E+04 | 6.6E+04 | 5.7E+04 | 7.3E+05 | 5.7E+05 | 5.8E+05 | 6.3E+05 |
| 7 | 387.3100 | 33.3 | 4.6E+04 | 3.2E+04 | 4.9E+04 | 4.2E+04 | 6.8E+05 | 5.0E+05 | 5.3E+05 | 5.7E+05 |
| 8 | 499.2879 | 29.0 | 3.9E+02 | 3.3E+03 | 6.3E+02 | 1.4E+03 | 6.1E+04 | 3.3E+04 | 5.1E+04 | 4.8E+04 |
| 9 | 505.3364 | 31.3 | 6.6E+04 | 5.4E+04 | 6.4E+04 | 6.1E+04 | 6.6E+05 | 4.7E+05 | 6.1E+05 | 5.8E+05 |
| 10 | 527.3188 | 31.3 | 2.3E+05 | 2.2E+05 | 2.7E+05 | 2.4E+05 | 3.2E+06 | 2.1E+06 | 2.7E+06 | 2.7E+06 |
| 11 | 543.2922 | 31.3 | 8.4E+03 | 1.1E+04 | 1.1E+04 | 1.0E+04 | 1.2E+05 | 7.5E+04 | 9.3E+04 | 9.6E+04 |
| 12 | 548.3781 | 32.8 | 3.9E+04 | 3.5E+04 | 3.6E+04 | 3.7E+04 | 3.8E+05 | 2.9E+05 | 2.8E+05 | 3.2E+05 |
| 13 | 549.3002 | 31.2 | 0.0E+00 | 1.1E+05 | 0.0E+00 | 3.7E+04 | 3.0E+05 | 2.7E+05 | 2.9E+05 | 2.9E+05 |

| # | m/z | RT (min) | Integrated mass ion intensity (MSII) | | | | | | | |
|---|---|---|---|---|---|---|---|---|---|---|
| | | | 100Hz-1 | 100Hz-2 | 100Hz-3 | 100Hz-avg | Con-1 | Con-2 | Con-3 | Con-avg |
| 14 | 550.3937 | 33.2 | 2.9E+04 | 1.8E+04 | 2.4E+04 | 2.4E+04 | 3.1E+05 | 2.2E+05 | 2.4E+05 | 2.6E+05 |
| 15 | 553.3331 | 32.8 | 0.0E+00 | 1.1E+04 | 0.0E+00 | 3.5E+03 | 4.5E+05 | 3.0E+05 | 3.4E+05 | 3.7E+05 |
| 16 | 555.3493 | 33.2 | 3.0E+04 | 2.3E+04 | 2.5E+04 | 2.6E+04 | 3.0E+05 | 2.3E+05 | 2.4E+05 | 2.6E+05 |
| 17 | 653.4370 | 26.6 | 2.7E+04 | 2.7E+04 | 3.2E+04 | 2.9E+04 | 4.1E+05 | 2.5E+05 | 3.4E+05 | 3.4E+05 |
| 18 | 667.4531 | 27.0 | 7.7E+03 | 0.0E+00 | 1.8E+04 | 8.7E+03 | 1.4E+05 | 8.6E+04 | 1.1E+05 | 1.1E+05 |
| 19 | 668.4206 | 30.4 | 0.0E+00 | 0.0E+00 | 4.8E+04 | 1.6E+04 | 1.7E+05 | 9.1E+04 | 1.7E+05 | 1.4E+05 |
| 20 | 696.4523 | 32.4 | 4.9E+03 | 0.0E+00 | 3.6E+04 | 1.4E+04 | 1.6E+05 | 9.6E+04 | 1.3E+05 | 1.3E+05 |
| 21 | 755.4519 | 30.3 | 0.0E+00 | 0.0E+00 | 0.0E+00 | 0.0E+00 | 6.9E+04 | 8.5E+04 | 1.2E+05 | 9.1E+04 |
| 22 | 915.5195 | 32.8 | 0.0E+00 | 3.3E+02 | 6.4E+02 | 3.2E+02 | 6.3E+04 | 4.7E+04 | 5.4E+04 | 5.5E+04 |
| 23 | 1,053.6279 | 31.3 | 0.0E+00 | 1.6E+03 | 0.0E+00 | 5.4E+02 | 2.8E+05 | 1.9E+05 | 2.5E+05 | 2.4E+05 |
| 24 | 1,105.6331 | 32.7 | 3.3E+04 | 1.2E+04 | 8.9E+03 | 1.8E+04 | 6.3E+04 | 6.8E+04 | 5.5E+04 | 6.2E+04 |

(c) Significantly decreased positive-mode ions in 100-Hz-treated *P. aeruginosa* extracellular samples (aMSII)

| # | m/z | RT (min) | Adjusted integrated mass ion intensity (aMSII) | | | | | | | |
|---|---|---|---|---|---|---|---|---|---|---|
| | | | 100Hz-1 | 100Hz-2 | 100Hz-3 | 100Hz-avg | Con-1 | Con-2 | Con-3 | Con-avg |
| 1 | 153.1271 | 32.6 | 1.0E+04 | 9.2E+03 | 7.9E+03 | 9.2E+03 | 1.9E+05 | 2.1E+05 | 1.9E+05 | 2.0E+05 |
| 2 | 308.2941 | 26.0 | 0.0E+00 | 4.1E+04 | 1.8E+04 | 1.9E+04 | 1.1E+05 | 1.2E+05 | 1.1E+05 | 1.1E+05 |
| 3 | 332.2222 | 28.8 | 5.3E+04 | 5.3E+04 | 4.6E+04 | 5.1E+04 | 1.7E+05 | 1.3E+05 | 1.2E+05 | 1.4E+05 |
| 4 | 341.2666 | 31.3 | 0.0E+00 | 0.0E+00 | 0.0E+00 | 0.0E+00 | 1.8E+05 | 1.3E+05 | 1.7E+05 | 1.6E+05 |
| 5 | 353.2296 | 30.2 | 0.0E+00 | 0.0E+00 | 2.9E+04 | 9.7E+03 | 1.4E+06 | 9.5E+05 | 1.1E+06 | 1.2E+06 |
| 6 | 385.2936 | 32.8 | 6.0E+04 | 4.5E+04 | 6.7E+04 | 5.7E+04 | 7.4E+05 | 5.6E+05 | 5.7E+05 | 6.2E+05 |
| 7 | 387.3100 | 33.3 | 4.5E+04 | 3.3E+04 | 5.0E+04 | 4.3E+04 | 6.9E+05 | 4.9E+05 | 5.2E+05 | 5.7E+05 |
| 8 | 499.2879 | 29.0 | 3.8E+02 | 3.4E+03 | 6.5E+02 | 1.5E+03 | 6.2E+04 | 3.2E+04 | 5.0E+04 | 4.8E+04 |
| 9 | 505.3364 | 31.3 | 6.5E+04 | 5.5E+04 | 6.5E+04 | 6.2E+04 | 6.7E+05 | 4.6E+05 | 5.9E+05 | 5.8E+05 |
| 10 | 527.3188 | 31.3 | 2.2E+05 | 2.3E+05 | 2.7E+05 | 2.4E+05 | 3.2E+06 | 2.1E+06 | 2.7E+06 | 2.7E+06 |
| 11 | 543.2922 | 31.3 | 8.3E+03 | 1.1E+04 | 1.1E+04 | 1.0E+04 | 1.2E+05 | 7.3E+04 | 9.0E+04 | 9.5E+04 |
| 12 | 548.3781 | 32.8 | 3.8E+04 | 3.7E+04 | 3.6E+04 | 3.7E+04 | 3.9E+05 | 2.9E+05 | 2.7E+05 | 3.1E+05 |
| 13 | 549.3002 | 31.2 | 0.0E+00 | 1.1E+05 | 0.0E+00 | 3.8E+04 | 3.1E+05 | 2.7E+05 | 2.8E+05 | 2.8E+05 |
| 14 | 550.3937 | 33.2 | 2.8E+04 | 1.9E+04 | 2.5E+04 | 2.4E+04 | 3.2E+05 | 2.2E+05 | 2.3E+05 | 2.6E+05 |
| 15 | 553.3331 | 32.8 | 0.0E+00 | 1.1E+04 | 0.0E+00 | 3.7E+03 | 4.6E+05 | 3.0E+05 | 3.3E+05 | 3.6E+05 |
| 16 | 555.3493 | 33.2 | 2.9E+04 | 2.3E+04 | 2.6E+04 | 2.6E+04 | 3.0E+05 | 2.3E+05 | 2.3E+05 | 2.5E+05 |
| 17 | 653.4370 | 26.6 | 2.7E+04 | 2.8E+04 | 3.3E+04 | 2.9E+04 | 4.2E+05 | 2.4E+05 | 3.3E+05 | 3.3E+05 |
| 18 | 667.4531 | 27.0 | 7.6E+03 | 0.0E+00 | 1.9E+04 | 8.8E+03 | 1.4E+05 | 8.5E+04 | 1.1E+05 | 1.1E+05 |
| 19 | 668.4206 | 30.4 | 0.0E+00 | 0.0E+00 | 4.9E+04 | 1.6E+04 | 1.7E+05 | 8.9E+04 | 1.7E+05 | 1.4E+05 |
| 20 | 696.4523 | 32.4 | 4.8E+03 | 0.0E+00 | 3.7E+04 | 1.4E+04 | 1.6E+05 | 9.4E+04 | 1.2E+05 | 1.3E+05 |
| 21 | 755.4519 | 30.3 | 0.0E+00 | 0.0E+00 | 0.0E+00 | 0.0E+00 | 6.9E+04 | 8.4E+04 | 1.2E+05 | 9.0E+04 |
| 22 | 915.5195 | 32.8 | 0.0E+00 | 3.4E+02 | 6.5E+02 | 3.3E+02 | 6.3E+04 | 4.6E+04 | 5.2E+04 | 5.4E+04 |
| 23 | 1,053.6279 | 31.3 | 0.0E+00 | 1.7E+03 | 0.0E+00 | 5.6E+02 | 2.8E+05 | 1.9E+05 | 2.5E+05 | 2.4E+05 |
| 24 | 1,105.6331 | 32.7 | 3.2E+04 | 1.2E+04 | 9.1E+03 | 1.8E+04 | 6.4E+04 | 6.7E+04 | 5.3E+04 | 6.1E+04 |

**S6 Table.** Significantly decreased negative-mode ions in 100-Hz-treated *P. aeruginosa* extracellular samples compared to the no-sound control in the metabolomics analysis. Data are shown as the mass-to-charge ratio (m/z), retention time (RT), (a) potential identification and MS/MS spectrum, (b) integrated mass ion intensity (MSII), and (c) adjusted mass ion intensity (aMSII). The (b) MSII and (c) aMSII values are for the three *P. aeruginosa* cultures with (100Hz-1–3) or without (Con-1–3) 100-Hz sound treatments and their respective averages (100Hz-avg and Con-avg, respectively) for a set of metabolomics analysis.

(a) Significantly decreased negative-mode ions in 100-Hz-treated *P. aeruginosa* extracellular samples (potential identification and MS/MS spectrum)

| # | m/z | RT (min) | Ion | Potential identification | MS/MS spectrum |
|---|---|---|---|---|---|
| 1 | 163.0642 | 23.9 | $[M-H]^-$ | Rhamnose fragment of MRL (10:0/10:0) | - |
| 2 | 169.1263 | 23.9 | $[M-H_2O-H]^-$ | Hydroxy FA (10:0) fragment of MRL (10:0/10:0) | - |
| 3 | 187.1365 | 24.1 | $[M-H]^-$ | Hydroxy FA (10:0) fragment of MRL (10:0/10:0) | - |
| 4 | 333.1946 | 23.9 | $[M-H]^-$ | MRL (10:0/10:0) – FA (10:0) fragment | - |
| 5 | 339.2570 | 23.9 | $[M-H]^-$ | MRL (10:0/10:0) – rhamnose fragment | - |
| 6 | 367.2898 | 26.2 | $[M-H]^-$ | MRL (10:0/12:0) – rhamnose fragment | - |
| 7 | 421.2612 | 23.9 | ? | Fragment of MRL (10:0/10:0) | - |
| 8 | 447.2775 | 25.3 | ? | Fragment of MRL (10:0/12:1) | - |
| 9 | 449.2920 | 26.2 | ? | Fragment of MRL (10:0/12:0) | - |
| 10 | 503.3304 | 23.9 | $[M-H]^-$ | MRL (10:0/10:0) | S62 |
| 11 | 525.3067 | 23.9 | $[M+Na-2H]^-$ | MRL (10:0/10:0) | - |
| 12 | 529.3448 | 25.3 | $[M-H]^-$ | MRL (10:0/12:1) | S63 |
| 13 | 531.3601 | 26.2 | $[M-H]^-$ | MRL (10:0/12:0) | S64 |
| 14 | 551.3234 | 25.3 | $[M+Na-2H]^-$ | MRL (10:0/12:1) | - |
| 15 | 553.3374 | 26.2 | $[M+Na-2H]^-$ | MRL (10:0/12:0) | - |
| 16 | 557.3746 | 27.6 | $[M-H]^-$ | MRL (12:0/12:1) or other side chains | - |
| 17 | 649.3862 | 23.7 | $[M-H]^-$ | DRL (10:0/10:0) | S65 |
| 18 | 675.4021 | 25.0 | $[M-H]^-$ | DRL (10:0/12:1) | S66 |
| 19 | 677.4191 | 25.9 | $[M-H]^-$ | DRL (10:0/12:0) | S67 |
| 20 | 697.3862 | 25.0 | $[M+Na-2H]^-$ | DRL (10:0/12:1) | - |
| 21 | 699.4003 | 26.0 | $[M+Na-2H]^-$ | DRL (10:0/12:0) | - |
| 22 | 705.4486 | 28.2 | $[M-H]^-$ | DRL (12:0/12:0) | S68 |
| 23 | 721.3828 | 26.0 | $[M+2Na-3H]^-$ | DRL (10:0/12:0) | - |
| 24 | 761.3735 | 26.0 | ? | Adduct ion of DRL (10:0/12:0) | - |
| 25 | 765.2622 | 26.2 | ? | Adduct ion of MRL (10:0/12:0) | - |
| 26 | 827.3446 | 26.0 | ? | Adduct ion of DRL (10:0/12:0) | - |
| 27 | 1,003.6097 | 25.9 | ? | Adduct ion of DRL (10:0/12:0) | - |
| 28 | 1,051.6205 | 23.9 | $[2M+2Na-3H]^-$ | MRL (10:0/10:0) | - |
| 29 | 1,073.6043 | 23.9 | $[2M+3Na-4H]^-$ | MRL (10:0/10:0) | - |

(b) Significantly decreased negative-mode ions in 100-Hz-treated *P. aeruginosa* extracellular samples (MSII)

| # | m/z | RT (min) | Integrated mass ion intensity (MSII) | | | | | | | |
|---|---|---|---|---|---|---|---|---|---|---|
| | | | 100Hz-1 | 100Hz-2 | 100Hz-3 | 100Hz-avg | Con-1 | Con-2 | Con-3 | Con-avg |
| 1 | 163.0642 | 23.9 | 3.0E+03 | 2.2E+03 | 1.8E+03 | 2.3E+03 | 1.2E+04 | 8.1E+03 | 1.4E+04 | 1.1E+04 |
| 2 | 169.1263 | 23.9 | 1.8E+04 | 1.7E+04 | 1.4E+04 | 1.6E+04 | 1.2E+05 | 1.0E+05 | 1.5E+05 | 1.2E+05 |
| 3 | 187.1365 | 24.1 | 9.1E+03 | 1.0E+04 | 6.3E+03 | 8.5E+03 | 2.0E+05 | 2.7E+05 | 2.6E+05 | 2.4E+05 |
| 4 | 333.1946 | 23.9 | 4.4E+04 | 3.8E+04 | 3.1E+04 | 3.8E+04 | 2.7E+05 | 2.4E+05 | 3.3E+05 | 2.8E+05 |
| 5 | 339.2570 | 23.9 | 4.7E+04 | 4.4E+04 | 3.3E+04 | 4.2E+04 | 3.2E+05 | 2.6E+05 | 3.8E+05 | 3.2E+05 |
| 6 | 367.2898 | 26.2 | 4.1E+03 | 3.5E+03 | 4.1E+03 | 3.9E+03 | 3.5E+04 | 4.3E+04 | 4.1E+04 | 4.0E+04 |
| 7 | 421.2612 | 23.9 | 3.3E+04 | 3.1E+04 | 7.9E+01 | 2.1E+04 | 1.3E+05 | 1.1E+05 | 1.4E+05 | 1.3E+05 |
| 8 | 447.2775 | 25.3 | 4.8E+03 | 3.6E+03 | 3.6E+03 | 4.0E+03 | 2.8E+04 | 2.6E+04 | 3.0E+04 | 2.8E+04 |
| 9 | 449.2920 | 26.2 | 3.5E+03 | 2.4E+03 | 3.7E+03 | 3.2E+03 | 2.9E+04 | 2.5E+04 | 2.9E+04 | 2.8E+04 |
| 10 | 503.3304 | 23.9 | 7.0E+05 | 6.4E+05 | 4.9E+05 | 6.1E+05 | 5.1E+06 | 4.2E+06 | 5.8E+06 | 5.0E+06 |
| 11 | 525.3067 | 23.9 | 4.5E+04 | 3.8E+04 | 3.0E+04 | 3.8E+04 | 1.7E+05 | 1.3E+05 | 1.6E+05 | 1.5E+05 |
| 12 | 529.3448 | 25.3 | 1.0E+05 | 8.6E+04 | 8.3E+04 | 9.1E+04 | 6.6E+05 | 6.2E+05 | 7.7E+05 | 6.9E+05 |
| 13 | 531.3601 | 26.2 | 7.1E+04 | 5.9E+04 | 7.1E+04 | 6.7E+04 | 8.1E+05 | 7.5E+05 | 8.2E+05 | 7.9E+05 |
| 14 | 551.3234 | 25.3 | 5.6E+03 | 1.2E+04 | 7.8E+03 | 8.5E+03 | 4.4E+04 | 4.0E+04 | 4.6E+04 | 4.3E+04 |
| 15 | 553.3374 | 26.2 | 1.4E+04 | 6.7E+03 | 2.0E+04 | 1.4E+04 | 5.4E+04 | 4.4E+04 | 4.9E+04 | 4.9E+04 |
| 16 | 557.3746 | 27.6 | 4.6E+03 | 3.7E+03 | 1.0E+03 | 3.1E+03 | 4.2E+04 | 3.4E+04 | 3.9E+04 | 3.8E+04 |
| 17 | 649.3862 | 23.7 | 7.3E+04 | 5.9E+04 | 1.1E+05 | 7.9E+04 | 4.8E+05 | 3.0E+05 | 5.4E+05 | 4.4E+05 |
| 18 | 675.4021 | 25.0 | 3.2E+04 | 2.7E+04 | 3.9E+04 | 3.3E+04 | 1.7E+05 | 1.0E+05 | 1.7E+05 | 1.5E+05 |
| 19 | 677.4191 | 25.9 | 5.7E+04 | 5.1E+04 | 8.1E+04 | 6.3E+04 | 4.2E+05 | 2.4E+05 | 3.6E+05 | 3.4E+05 |
| 20 | 697.3862 | 25.0 | 4.7E+03 | 5.3E+03 | 2.5E+03 | 4.2E+03 | 2.5E+04 | 2.6E+04 | 2.3E+04 | 2.5E+04 |
| 21 | 699.4003 | 26.0 | 9.5E+03 | 9.4E+03 | 1.2E+04 | 1.0E+04 | 5.0E+04 | 3.1E+04 | 4.0E+04 | 4.1E+04 |
| 22 | 705.4486 | 28.2 | 7.8E+03 | 1.0E+03 | 4.4E+02 | 3.1E+03 | 3.5E+04 | 2.4E+04 | 2.7E+04 | 2.9E+04 |
| 23 | 721.3828 | 26.0 | 4.6E+03 | 3.7E+03 | 0.0E+00 | 2.8E+03 | 1.3E+04 | 7.9E+03 | 1.1E+04 | 1.1E+04 |
| 24 | 761.3735 | 26.0 | 4.9E+03 | 3.7E+03 | 1.0E+02 | 2.9E+03 | 1.7E+04 | 1.0E+04 | 1.3E+04 | 1.3E+04 |
| 25 | 765.2622 | 26.2 | 2.4E+03 | 4.3E+03 | 2.6E+03 | 3.1E+03 | 1.5E+04 | 8.9E+03 | 1.3E+04 | 1.2E+04 |
| 26 | 827.3446 | 26.0 | 4.1E+03 | 3.5E+03 | 3.1E+02 | 2.6E+03 | 1.4E+04 | 7.4E+03 | 1.0E+04 | 1.1E+04 |
| 27 | 1,003.6097 | 25.9 | 3.6E+03 | 3.0E+03 | 0.0E+00 | 2.2E+03 | 2.1E+04 | 1.3E+04 | 1.2E+04 | 1.5E+04 |
| 28 | 1,051.6205 | 23.9 | 8.0E+03 | 7.0E+03 | 4.7E+03 | 6.6E+03 | 6.1E+04 | 3.7E+04 | 6.2E+04 | 5.3E+04 |
| 29 | 1,073.6043 | 23.9 | 6.5E+03 | 5.1E+03 | 3.6E+03 | 5.1E+03 | 3.1E+04 | 1.6E+04 | 2.8E+04 | 2.5E+04 |

(c) Significantly decreased negative-mode ions in 100-Hz-treated *P. aeruginosa* extracellular samples (aMSII)

| # | m/z | RT (min) | Adjusted integrated mass ion intensity (aMSII) | | | | | | | |
|---|---|---|---|---|---|---|---|---|---|---|
| | | | 100Hz-1 | 100Hz-2 | 100Hz-3 | 100Hz-avg | Con-1 | Con-2 | Con-3 | Con-avg |
| 1 | 163.0642 | 23.9 | 2.9E+03 | 2.3E+03 | 1.8E+03 | 2.3E+03 | 1.2E+04 | 8.0E+03 | 1.3E+04 | 1.1E+04 |
| 2 | 169.1263 | 23.9 | 1.8E+04 | 1.7E+04 | 1.4E+04 | 1.6E+04 | 1.2E+05 | 9.8E+04 | 1.4E+05 | 1.2E+05 |
| 3 | 187.1365 | 24.1 | 9.0E+03 | 1.1E+04 | 6.4E+03 | 8.6E+03 | 2.0E+05 | 2.7E+05 | 2.5E+05 | 2.4E+05 |
| 4 | 333.1946 | 23.9 | 4.4E+04 | 3.9E+04 | 3.2E+04 | 3.8E+04 | 2.7E+05 | 2.4E+05 | 3.2E+05 | 2.8E+05 |
| 5 | 339.2570 | 23.9 | 4.6E+04 | 4.6E+04 | 3.4E+04 | 4.2E+04 | 3.3E+05 | 2.6E+05 | 3.7E+05 | 3.2E+05 |
| 6 | 367.2898 | 26.2 | 4.0E+03 | 3.6E+03 | 4.1E+03 | 3.9E+03 | 3.6E+04 | 4.2E+04 | 4.0E+04 | 3.9E+04 |
| 7 | 421.2612 | 23.9 | 3.2E+04 | 3.2E+04 | 8.1E+01 | 2.1E+04 | 1.3E+05 | 1.1E+05 | 1.4E+05 | 1.3E+05 |
| 8 | 447.2775 | 25.3 | 4.8E+03 | 3.7E+03 | 3.7E+03 | 4.0E+03 | 2.8E+04 | 2.5E+04 | 2.9E+04 | 2.7E+04 |
| 9 | 449.2920 | 26.2 | 3.5E+03 | 2.4E+03 | 3.8E+03 | 3.2E+03 | 2.9E+04 | 2.5E+04 | 2.8E+04 | 2.7E+04 |
| 10 | 503.3304 | 23.9 | 6.9E+05 | 6.6E+05 | 5.0E+05 | 6.2E+05 | 5.1E+06 | 4.2E+06 | 5.6E+06 | 5.0E+06 |
| 11 | 525.3067 | 23.9 | 4.4E+04 | 4.0E+04 | 3.0E+04 | 3.8E+04 | 1.7E+05 | 1.3E+05 | 1.6E+05 | 1.5E+05 |
| 12 | 529.3448 | 25.3 | 1.0E+05 | 8.9E+04 | 8.4E+04 | 9.2E+04 | 6.7E+05 | 6.1E+05 | 7.5E+05 | 6.8E+05 |
| 13 | 531.3601 | 26.2 | 7.0E+04 | 6.1E+04 | 7.3E+04 | 6.8E+04 | 8.2E+05 | 7.3E+05 | 8.0E+05 | 7.8E+05 |
| 14 | 551.3234 | 25.3 | 5.5E+03 | 1.2E+04 | 8.0E+03 | 8.6E+03 | 4.4E+04 | 3.9E+04 | 4.5E+04 | 4.3E+04 |
| 15 | 553.3374 | 26.2 | 1.4E+04 | 7.0E+03 | 2.1E+04 | 1.4E+04 | 5.4E+04 | 4.3E+04 | 4.8E+04 | 4.8E+04 |
| 16 | 557.3746 | 27.6 | 4.5E+03 | 3.8E+03 | 1.0E+03 | 3.1E+03 | 4.2E+04 | 3.3E+04 | 3.8E+04 | 3.8E+04 |
| 17 | 649.3862 | 23.7 | 7.1E+04 | 6.1E+04 | 1.1E+05 | 8.0E+04 | 4.9E+05 | 3.0E+05 | 5.2E+05 | 4.4E+05 |
| 18 | 675.4021 | 25.0 | 3.2E+04 | 2.8E+04 | 3.9E+04 | 3.3E+04 | 1.7E+05 | 1.0E+05 | 1.6E+05 | 1.5E+05 |
| 19 | 677.4191 | 25.9 | 5.6E+04 | 5.3E+04 | 8.3E+04 | 6.4E+04 | 4.2E+05 | 2.4E+05 | 3.5E+05 | 3.4E+05 |
| 20 | 697.3862 | 25.0 | 4.6E+03 | 5.5E+03 | 2.5E+03 | 4.2E+03 | 2.5E+04 | 2.6E+04 | 2.2E+04 | 2.4E+04 |
| 21 | 699.4003 | 26.0 | 9.4E+03 | 9.7E+03 | 1.2E+04 | 1.0E+04 | 5.1E+04 | 3.1E+04 | 3.9E+04 | 4.0E+04 |
| 22 | 705.4486 | 28.2 | 7.7E+03 | 1.0E+03 | 4.5E+02 | 3.1E+03 | 3.6E+04 | 2.3E+04 | 2.6E+04 | 2.8E+04 |
| 23 | 721.3828 | 26.0 | 4.6E+03 | 3.8E+03 | 0.0E+00 | 2.8E+03 | 1.4E+04 | 7.7E+03 | 1.1E+04 | 1.1E+04 |
| 24 | 761.3735 | 26.0 | 4.8E+03 | 3.8E+03 | 1.0E+02 | 2.9E+03 | 1.7E+04 | 9.8E+03 | 1.3E+04 | 1.3E+04 |
| 25 | 765.2622 | 26.2 | 2.4E+03 | 4.4E+03 | 2.6E+03 | 3.1E+03 | 1.5E+04 | 8.7E+03 | 1.2E+04 | 1.2E+04 |
| 26 | 827.3446 | 26.0 | 4.0E+03 | 3.6E+03 | 3.2E+02 | 2.6E+03 | 1.4E+04 | 7.2E+03 | 1.0E+04 | 1.1E+04 |
| 27 | 1,003.6097 | 25.9 | 3.5E+03 | 3.1E+03 | 0.0E+00 | 2.2E+03 | 2.1E+04 | 1.3E+04 | 1.2E+04 | 1.5E+04 |
| 28 | 1,051.6205 | 23.9 | 7.8E+03 | 7.2E+03 | 4.8E+03 | 6.6E+03 | 6.2E+04 | 3.7E+04 | 6.0E+04 | 5.3E+04 |
| 29 | 1,073.6043 | 23.9 | 6.4E+03 | 5.3E+03 | 3.6E+03 | 5.1E+03 | 3.1E+04 | 1.6E+04 | 2.7E+04 | 2.5E+04 |

**S7 Table.** Significantly decreased positive-mode ions in 100-Hz-treated *P. aeruginosa* intracellular samples compared to the no-sound control in the metabolomics analysis. Data are shown as the mass-to-charge ratio (m/z), retention time (RT), (a) potential identification and MS/MS spectrum, (b) integrated mass ion intensity (MSII), and (c) adjusted mass ion intensity (aMSII). The (b) MSII and (c) aMSII values are for the three *P. aeruginosa* cultures with (100Hz-1–3) or without (Con-1–3) 100-Hz sound treatments and their respective averages (100Hz-avg and Con-avg, respectively) for a set of metabolomics analysis.

(a) Significantly decreased positive-mode ions in 100-Hz-treated *P. aeruginosa* intracellular samples (potential identification and MS/MS spectrum)

| # | m/z | RT (min) | Ion | Potential identification | MS/MS spectrum |
|---|---|---|---|---|---|
| 1 | 270.1904 | 9.6 | ? | ? | - |

(b) Significantly decreased positive-mode ions in 100-Hz-treated *P. aeruginosa* intracellular samples (MSII)

| # | m/z | RT (min) | Integrated mass ion intensity (MSII) | | | | | | | |
|---|---|---|---|---|---|---|---|---|---|---|
| | | | 100Hz-1 | 100Hz-2 | 100Hz-3 | 100Hz-avg | Con-1 | Con-2 | Con-3 | Con-avg |
| 1 | 270.1904 | 9.6 | 0.0E+00 | 0.0E+00 | 0.0E+00 | 0.0E+00 | 1.2E+05 | 9.9E+04 | 1.2E+05 | 1.1E+05 |

(c) Significantly decreased positive-mode ions in 100-Hz-treated *P. aeruginosa* intracellular samples (aMSII)

| # | m/z | RT (min) | Adjusted integrated mass ion intensity (aMSII) | | | | | | | |
|---|---|---|---|---|---|---|---|---|---|---|
| | | | 100Hz-1 | 100Hz-2 | 100Hz-3 | 100Hz-avg | Con-1 | Con-2 | Con-3 | Con-avg |
| 1 | 270.1904 | 9.6 | 0.0E+00 | 0.0E+00 | 0.0E+00 | 0.0E+00 | 1.2E+05 | 9.8E+04 | 1.2E+05 | 1.1E+05 |

**S8 Table.** Levels of other known metabolites in 100-Hz-treated *P. aeruginosa* extracellular and intracellular samples compared to the no-sound control in the metabolomics analysis. Data are shown as metabolite class and acyl chain, (a) integrated mass ion intensity (MSII), and (b) adjusted mass ion intensity (aMSII) of ions as indicated in the table in the manuscript. The (a) MSII and (b) aMSII values are for the three *P. aeruginosa* cultures with (100Hz-1–3) or without (Con-1–3) 100-Hz sound treatments and their respective averages (100Hz-avg and Con-avg, respectively) for a set of metabolomics analysis.

(a) Other known metabolites in 100-Hz-treated *P. aeruginosa* extracellular and intracellular samples (MSII)

| # | Metabolite class and acyl chain | Integrated mass ion intensity (MSII) | | | | | | | |
|---|---|---|---|---|---|---|---|---|---|
| | | 100Hz-1 | 100Hz-2 | 100Hz-3 | 100Hz-avg | Con-1 | Con-2 | Con-3 | Con-avg |
| | Other extracellular metabolites | | | | | | | | |
| 1 | 3-oxo-$C_{12}$-HSL | 1.4E+07 | 1.2E+07 | 1.3E+07 | 1.3E+07 | 1.3E+07 | 1.2E+07 | 1.2E+07 | 1.2E+07 |
| | Other intracellular metabolites | | | | | | | | |
| | Phosphatidylethanolamine (PE) | | | | | | | | |
| 2 | 16:0/18:1 | 3.1E+07 | 3.1E+07 | 2.9E+07 | 3.0E+07 | 2.7E+07 | 3.0E+07 | 3.2E+07 | 3.0E+07 |
| 3 | 18:1/18:1 | 7.1E+06 | 6.6E+06 | 4.6E+06 | 6.1E+06 | 6.6E+06 | 7.3E+06 | 6.4E+06 | 6.8E+06 |
| | Phosphatidylglycerol (PG) | | | | | | | | |
| 4 | 16:0/18:1 | 1.3E+07 | 1.3E+07 | 8.2E+06 | 1.1E+07 | 3.2E+06 | 1.5E+07 | 1.8E+07 | 1.2E+07 |
| 5 | 18:1/18:1 | 7.9E+06 | 7.7E+06 | 7.3E+06 | 7.7E+06 | 1.2E+07 | 8.1E+06 | 8.8E+06 | 9.7E+06 |
| | Diacylglycerol (DAG) | | | | | | | | |
| 6 | 16:0/18:1 | 7.5E+06 | 9.3E+06 | 1.1E+07 | 9.1E+06 | 4.8E+06 | 5.0E+06 | 6.0E+06 | 5.3E+06 |
| 7 | 18:1/18:1 | 2.5E+06 | 3.0E+06 | 4.2E+06 | 3.3E+06 | 1.8E+06 | 1.7E+06 | 2.5E+06 | 2.0E+06 |

(b) Other known metabolites in 100-Hz-treated *P. aeruginosa* extracellular and intracellular samples (aMSII)

| # | Metabolite class and acyl chain | Adjusted integrated mass ion intensity (aMSII) | | | | | | | |
|---|---|---|---|---|---|---|---|---|---|
| | | 100Hz-1 | 100Hz-2 | 100Hz-3 | 100Hz-avg | Con-1 | Con-2 | Con-3 | Con-avg |
| | Other extracellular metabolites | | | | | | | | |
| 1 | 3-oxo-$C_{12}$-HSL | 1.5E+07 | 1.3E+07 | 1.3E+07 | 1.3E+07 | 1.4E+07 | 1.1E+07 | 1.2E+07 | 1.2E+07 |
| | Other intracellular metabolites | | | | | | | | |
| | Phosphatidylethanolamine (PE) | | | | | | | | |
| 2 | 16:0/18:1 | 3.1E+07 | 3.2E+07 | 3.0E+07 | 3.1E+07 | 2.7E+07 | 3.0E+07 | 3.1E+07 | 2.9E+07 |
| 3 | 18:1/18:1 | 7.0E+06 | 6.9E+06 | 4.7E+06 | 6.2E+06 | 6.6E+06 | 7.2E+06 | 6.2E+06 | 6.7E+06 |
| | Phosphatidylglycerol (PG) | | | | | | | | |
| 4 | 16:0/18:1 | 1.3E+07 | 1.3E+07 | 8.4E+06 | 1.2E+07 | 3.3E+06 | 1.5E+07 | 1.7E+07 | 1.2E+07 |
| 5 | 18:1/18:1 | 7.8E+06 | 8.0E+06 | 7.5E+06 | 7.7E+06 | 1.2E+07 | 8.0E+06 | 8.6E+06 | 9.6E+06 |
| | Diacylglycerol (DAG) | | | | | | | | |
| 6 | 16:0/18:1 | 7.6E+06 | 9.4E+06 | 1.0E+07 | 9.1E+06 | 4.9E+06 | 4.9E+06 | 5.9E+06 | 5.2E+06 |
| 7 | 18:1/18:1 | 2.5E+06 | 3.1E+06 | 4.2E+06 | 3.3E+06 | 1.8E+06 | 1.7E+06 | 2.4E+06 | 2.0E+06 |

**S9 Table.** List of metabolite standards with MS/MS spectra in the positive and negative ion modes

| Metabolites | Ion | m/z | MS/MS spectrum |
|---|---|---|---|
| Metabolite standards in the positive ion mode | | | |
| AHQ (7:0) (HHQ) | $[M + H]^+$ | 244.1701 | S21 |
| AHQ (9:0) (HNQ) | $[M + H]^+$ | 272.2014 | S22 |
| 3-oxo-$C_{12}$-HSL | $[M + H]^+$ | 298.2018 | S23 |
| MRL (10:0/10:0) | $[M + NH_4]^+$ | 522.3642 | S24 |
| MRL (10:0/10:0) | $[M + Na]^+$ | 527.3196 | S25 |
| DRL (10:0/10:0) | $[M + NH_4]^+$ | 668.4221 | S26 |
| Metabolite standards in the negative ion mode | | | |
| AHQ (7:0) (HHQ) | $[M – H]^–$ | 242.1545 | S27 |
| AHQ (9:0) (HNQ) | $[M – H]^–$ | 270.1858 | S28 |
| $C_n$-PQS (9:0) | $[M – H]^–$ | 286.1807 | S29 |
| NAE (18:1) | $[M – H]^–$ | 324.2903 | S30 |
| MRL (8:0/10:0) | $[M – H]^–$ | 475.2907 | S31 |
| MRL (10:0/10:0) | $[M – H]^–$ | 503.3220 | S32 |
| MRL (10:0/12:1) | $[M – H]^–$ | 529.3377 | S33 |
| MRL (10:0/12:0) | $[M – H]^–$ | 531.3533 | S34 |
| DRL (8:0/10:0) | $[M – H]^–$ | 621.3486 | S35 |
| DRL (10:0/10:0) | $[M – H]^–$ | 649.3799 | S36 |
| DRL (10:0/12:1) | $[M – H]^–$ | 675.3956 | S37 |
| DRL (10:0/12:0) | $[M – H]^–$ | 677.4112 | S38 |
| DRL (12:0/12:0) | $[M – H]^–$ | 705.4431 | S39 |

**S1 Fig. List of MS/MS Spectra (**as referred to in S1–S6 and S9 Tables).

**List of categories**


Metabolite standards in the positive ion mode (see S9 Table) S21

Metabolite standards in the negative ion mode (see S9 Table) S27

Positive-mode ions that show elevated levels in 100-Hz-stimulated intracellular samples (see S3 Table) S40

Negative-mode ions that show elevated levels in 100-Hz-stimulated intracellular samples (see S4 Table) S42

Positive-mode ions that show decreased levels in 100-Hz-stimulated extracellular samples (see S5 Table) S54

Negative-mode ions that show decreased levels in 100-Hz-stimulated extracellular samples (see S6 Table) S62

**Metabolite standards in the positive ion mode**

2-alkyl-4-hydroxyquinoline (AHQ (7:0); HHQ) – $[M + H]^+$ 244.1701 at 23.5 min (standard)

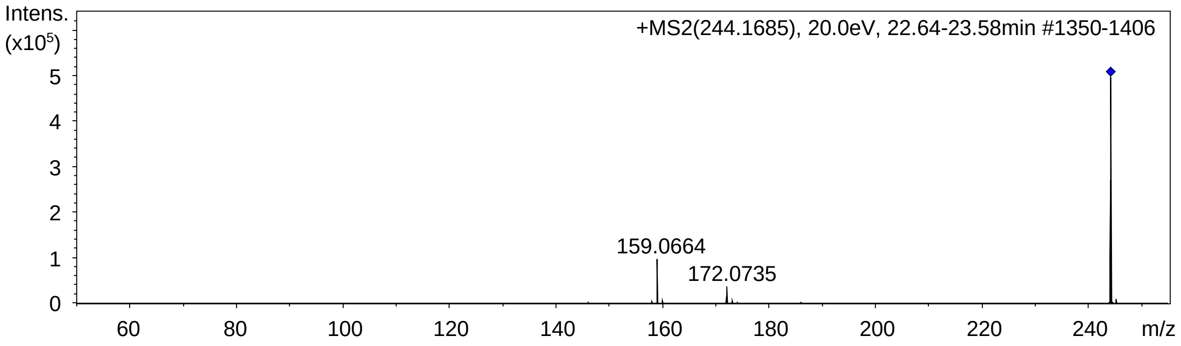


| Measured m/z | Possible MF | Possible structural assignment |
|---|---|---|
| 159.0664 | $C_{10}H_9NO^+$ | HHQ – $C_6H_{13}$ |
| 172.0735 | $C_{11}H_{10}NO^+$ | HHQ – $C_5H_{12}$ |

2-alkyl-4-hydroxyquinoline (AHQ (9:0); HNQ) – $[M + H]^+$ 272.2014 at 27.2 min (standard)

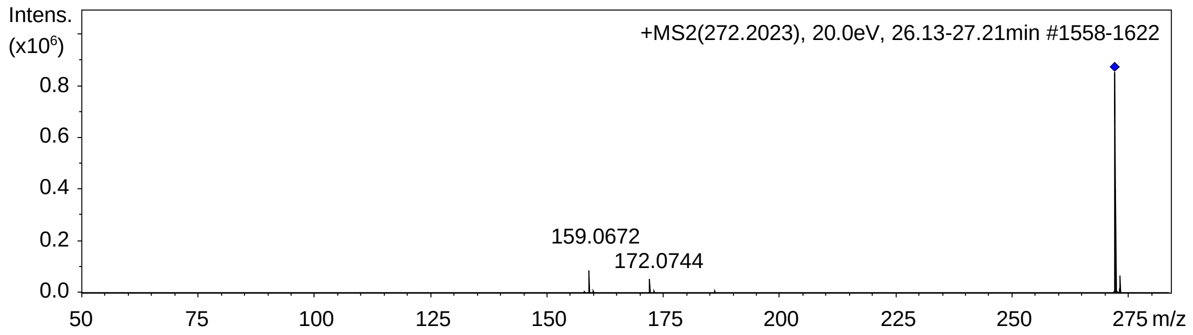


| Measured m/z | Possible MF | Possible structural assignment |
|---|---|---|
| 159.0672 | $C_{10}H_9NO^+$ | HNQ – $C_8H_{17}$ |
| 172.0744 | $C_{11}H_{10}NO^+$ | HNQ – $C_7H_{16}$ |
| 186.0899 | $C_{12}H_{12}NO^+$ | HNQ – $C_6H_{14}$ |

*N*-(3-oxododecanoyl)-L-homoserine lactone (3-oxo-$C_{12}$-HSL) – $[M + H]^+$ 298.2018 at 24.4 min (standard)

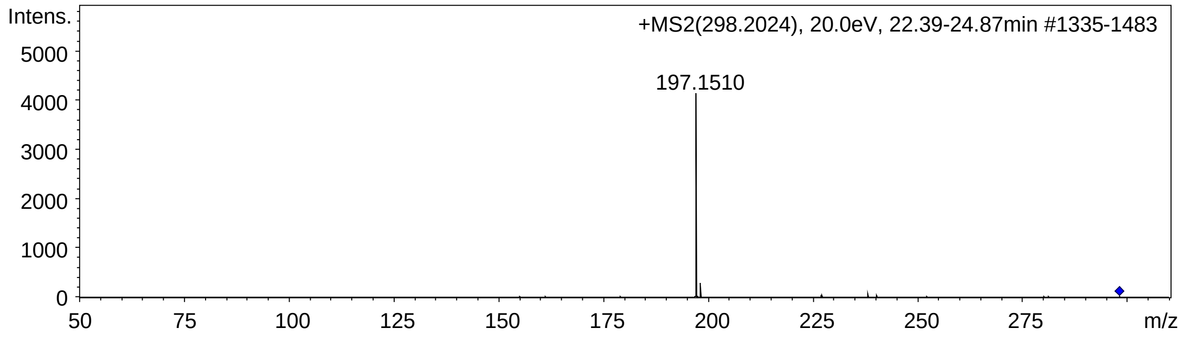


| Measured m/z | Possible MF | Possible structural assignment |
|---|---|---|
| 197.1510 | $C_{12}H_{21}O_2^+$ | acylium ion of 3-oxododecanoyl fragment ((3-oxododecylidyne) oxonium) |

Monorhamnolipid (10:0/10:0) – $[M + NH_4]^+$ 522.3642 at 31.3 min (standard)

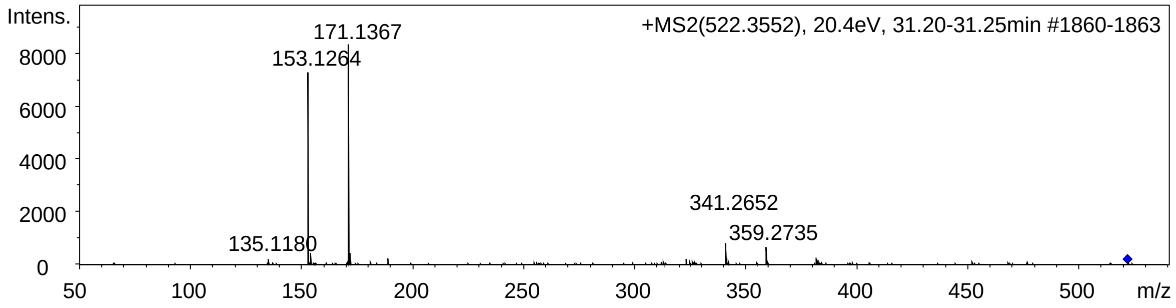


| Measured m/z | Possible MF | Possible structural assignment |
|---|---|---|
| 135.1180 | $C_{10}H_{15}^+$ | acylium ion of hydroxy FA (10:0) – $2H_2O$ |
| 153.1264 | $C_{10}H_{17}O^+$ | acylium ion of hydroxy FA (10:0) – $H_2O$ |
| 171.1367 | $C_{10}H_{19}O_2^+$ | acylium ion of hydroxy FA (10:0) |
| 189.1453 | $C_{10}H_{21}O_3^+$ | hydroxy FA (10:0) |
| 341.2652 | $C_{20}H_{37}O_4^+$ | HAA (10:0/10:0) – $H_2O$ |
| 359.2735 | $C_{20}H_{39}O_5^+$ | HAA (10:0/10:0) |

Monorhamnolipid (10:0/10:0) – $[M + Na]^+$ 527.3196 at 31.3 min (standard)

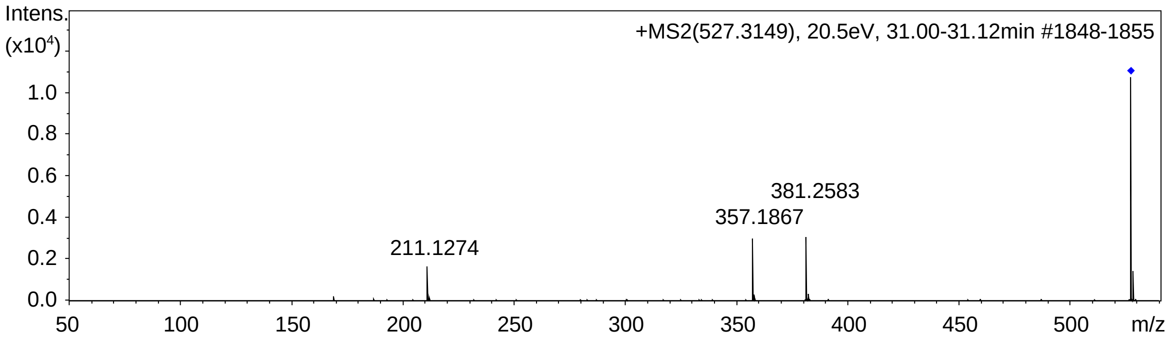


| Measured m/z | Possible MF | Possible structural assignment |
|---|---|---|
| 169.0441 | $C_6H_{10}NaO_4^+$ | rhamnose – $H_2O$ (sodium adduct) |
| 211.1274 | $C_{10}H_{20}NaO_3^+$ | hydroxy FA (10:0) (sodium adduct) |
| 357.1867 | $C_{16}H_{30}NaO_7^+$ | monorhamnolipid (10:0/10:0) – FA(10:0) (sodium adduct) |
| 381.2583 | $C_{20}H_{38}NaO_5^+$ | HAA (10:0/10:0) (sodium adduct) |

Dirhamnolipid (10:0/10:0) – $[M + NH_4]^+$ 668.4221 at 30.4 min (standard)

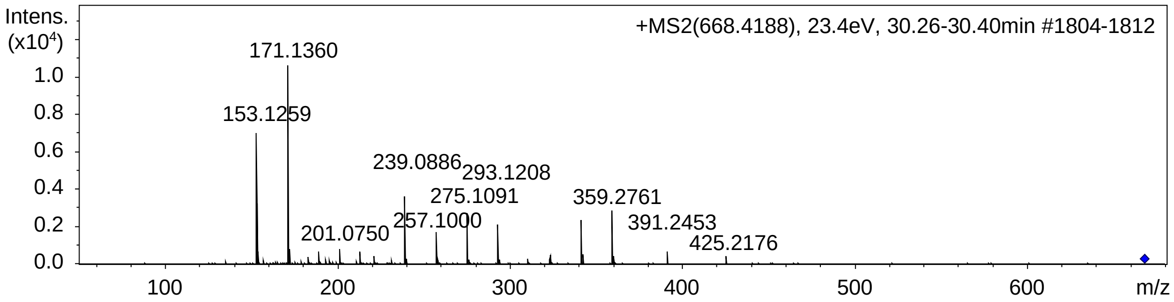


| Measured m/z | Possible MF | Possible structural assignment |
|---|---|---|
| 153.1259 | $C_{10}H_{17}O^+$ | acylium ion of hydroxy FA (10:0) – $H_2O$ |
| 171.1360 | $C_{10}H_{19}O_2^+$ | acylium ion of hydroxy FA (10:0) |
| 189.1477 | $C_{10}H_{21}O_3^+$ | hydroxy FA (10:0) |
| 201.0750 | $C_7H_{14}NaO_5^+$ | rhamnose + $CH_2$ (sodium adduct) |
| 213.0750 | $C_8H_{14}NaO_5^+$ | rhamnose + $C_2H_2$ (sodium adduct) |
| 239.0886 | $C_{10}H_{16}NaO_5^+$ | rhamnose + $C_4H_4$ (sodium adduct) |
| 257.1000 | $C_{10}H_{18}NaO_6^+$ | rhamnose + $C_4H_6O$ (sodium adduct) |
| 275.1090 | $C_{10}H_{20}NaO_7^+$ | rhamnose + $C_4H_8O_2$ (sodium adduct) |
| 293.1210 | $C_{10}H_{22}NaO_8^+$ | rhamnose + $C_4H_{10}O_3$ (sodium adduct) |
| 323.2530 | $C_{20}H_{35}O_3^+$ | HAA (10:0/10:0) – $2H_2O$ |
| 341.2677 | $C_{20}H_{37}O_4^+$ | HAA (10:0/10:0) – $H_2O$ |
| 359.2761 | $C_{20}H_{39}O_5^+$ | HAA (10:0/10:0) |

**Metabolite standards in the negative ion mode**

2-alkyl-4-hydroxyquinoline (AHQ (7:0); HHQ) – $[M - H]^-$ 242.1545 at 23.4 min (standard)

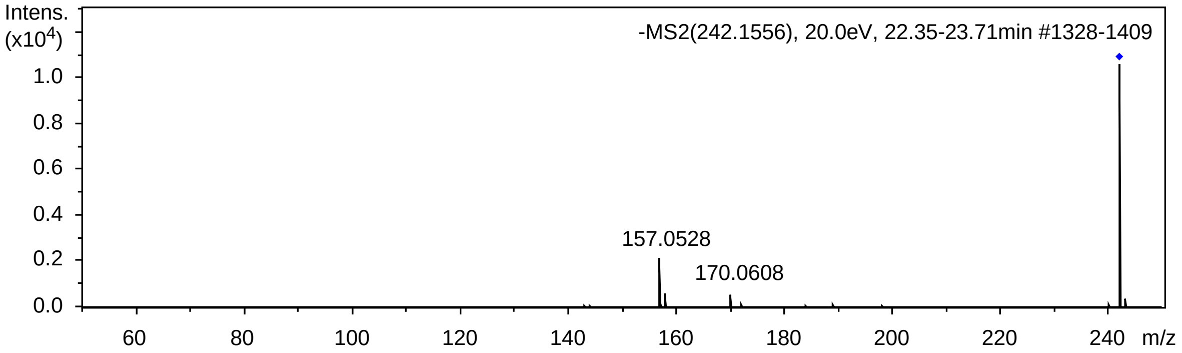


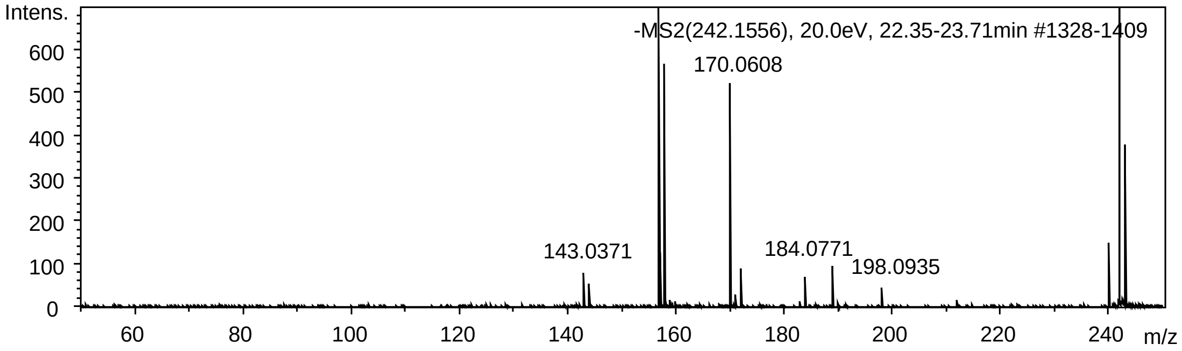


| Measured m/z | Possible MF | Possible structural assignment |
|---|---|---|
| 143.0371 | $C_9H_5NO^-$ | 4-hydroxyquinoline |
| 157.0528 | $C_{10}H_7NO^-$ | HNQ – $C_6H_{13}$ |
| 170.0608 | $C_{11}H_8NO^-$ | HNQ – $C_5H_{12}$ |
| 184.0771 | $C_{12}H_{10}NO^-$ | HNQ – $C_4H_{10}$ |
| 198.0935 | $C_{13}H_{12}NO^-$ | HNQ – $C_3H_8$ |

2-alkyl-4-hydroxyquinoline (AHQ (9:0); HNQ) – $[M – H]^-$ 270.1858 at 27.9 min (standard)

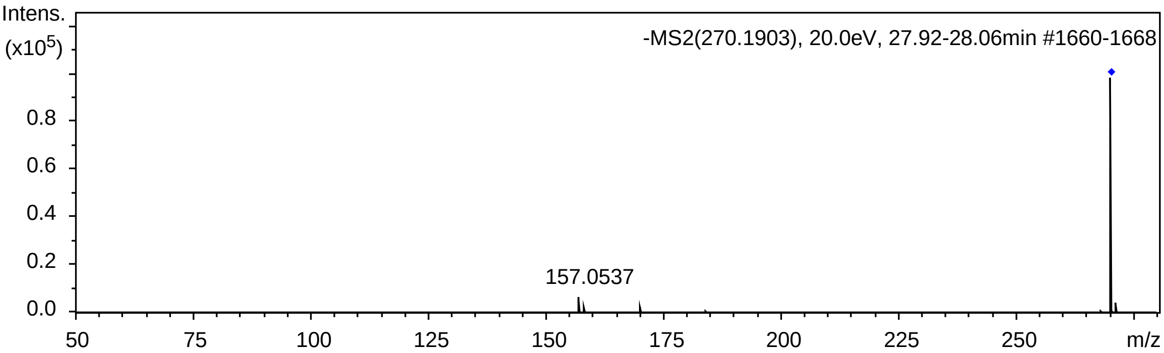


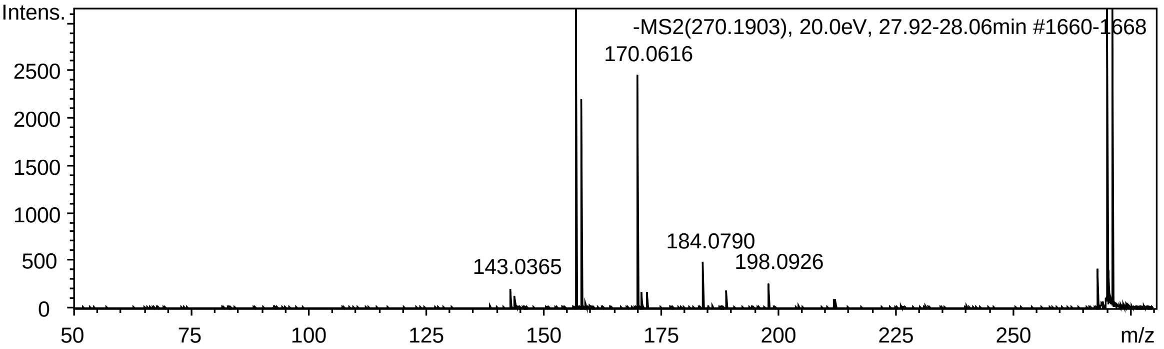


| Measured m/z | Possible MF | Possible structural assignment |
|---|---|---|
| 143.0365 | $C_9H_5NO^-$ | 4-hydroxyquinoline |
| 157.0537 | $C_{10}H_7NO^-$ | HNQ – $C_8H_{17}$ |
| 170.0616 | $C_{11}H_8NO^-$ | HNQ – $C_7H_{16}$ |
| 184.0790 | $C_{12}H_{10}NO^-$ | HNQ – $C_6H_{14}$ |
| 198.0926 | $C_{13}H_{12}NO^-$ | HNQ – $C_5H_{12}$ |

2-nonyl-3-hydroxy-4-quinolone (9:0) ($C_n$-PQS (9:0)) – $[M – H]^-$ 286.1807 at 29.4 min (standard)

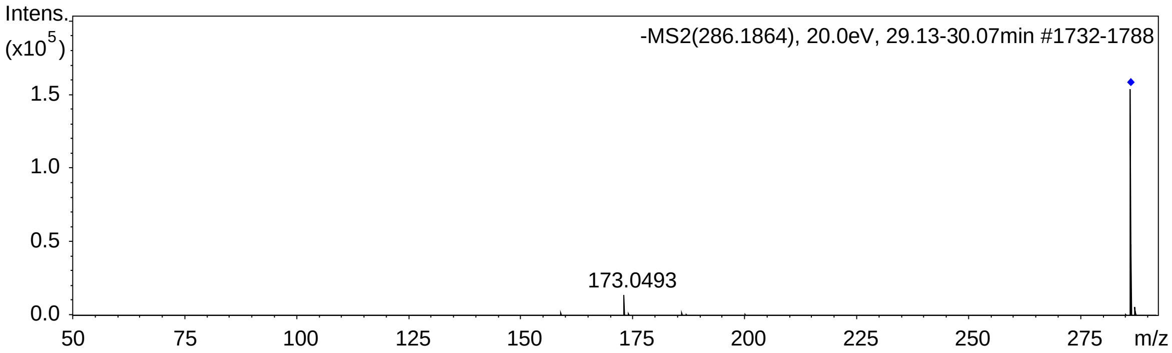


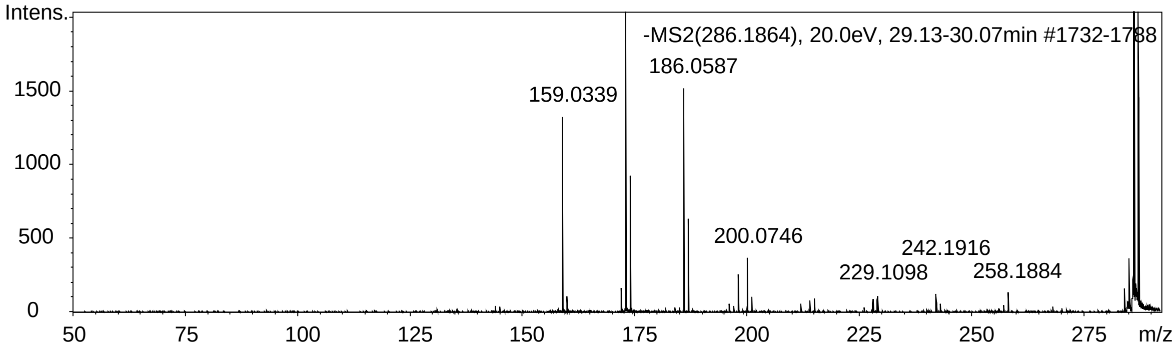


| Measured m/z | Possible MF | Possible structural assignment |
|---|---|---|
| 159.0339 | $C_9H_5NO_2^-$ | 3-hydroxy-4-quinolone |
| 173.0493 | $C_{10}H_7NO_2^-$ | $C_n$-PQS (9:0) – $C_8H_{17}$ |
| 186.0587 | $C_{11}H_8NO_2^-$ | $C_n$-PQS (9:0) – $C_7H_{16}$ |
| 198.0966 | $C_{13}H_{12}NO^-$ | $C_n$-PQS (9:0) – $C_5H_{12}$ |
| 200.0746 | $C_{12}H_{10}NO_2^-$ | $C_n$-PQS (9:0) – $C_6H_{14}$ |
| 229.1098 | $C_{14}H_{15}NO_2^-$ | $C_n$-PQS (9:0) – $C_4H_9$ |
| 242.1916 | $C_{17}H_{24}N^-$ | $C_n$-PQS (9:0) – CO – $H_2O$ + $H_2$ |
| 258.1884 | $C_{17}H_{24}NO^-$ | $C_n$-PQS (9:0) – CO |

*N*-oleoylethanolamine (NAE (18:1)) – [M – H]⁻ 324.2903 at 37.4 min (standard)

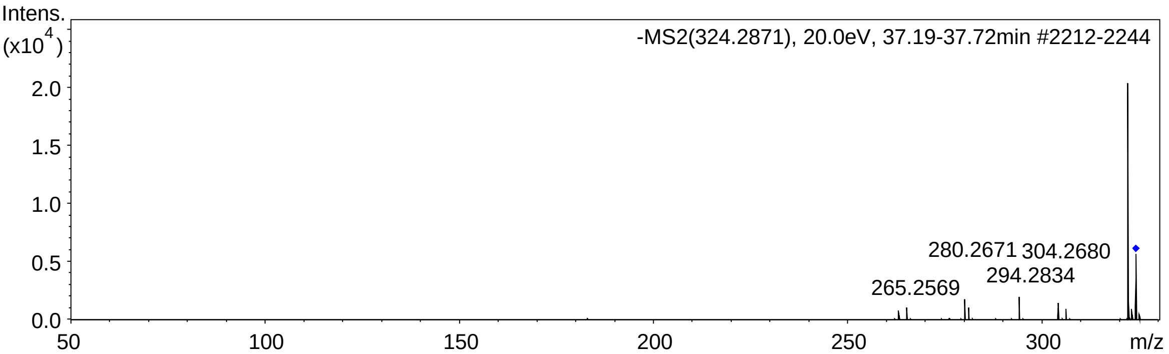


| Measured m/z | Possible MF | Possible structural assignment |
|---|---|---|
| 263.2441 | $C_{18}H_{31}O^{-}$ | *N*-acylethanolamine (18:1) – ethanolamine – $H_2$ |
| 265.2569 | $C_{18}H_{33}O^{-}$ | *N*-acylethanolamine (18:1) – ethanolamine |
| 280.2671 | $C_{18}H_{34}NO^{-}$ | *N*-acylethanolamine (18:1) – ethanol |
| 281.2522 | $C_{18}H_{33}O_2^{-}$ | FA (18:1) (displacement of ethanolamine with water) |
| 294.2834 | $C_{19}H_{36}NO^{-}$ | *N*-acylethanolamine (18:1) – methanol |
| 304.2680 | $C_{20}H_{34}NO^{-}$ | *N*-acylethanolamine (18:1) – $H_2O$ – $H_2$ |
| 306.2833 | $C_{20}H_{36}NO^{-}$ | *N*-acylethanolamine (18:1) – $H_2O$ |

Monorhamnolipid (8:0/10:0) – [M – H]$^{–}$ 475.2907 at 17.9 min (standard)

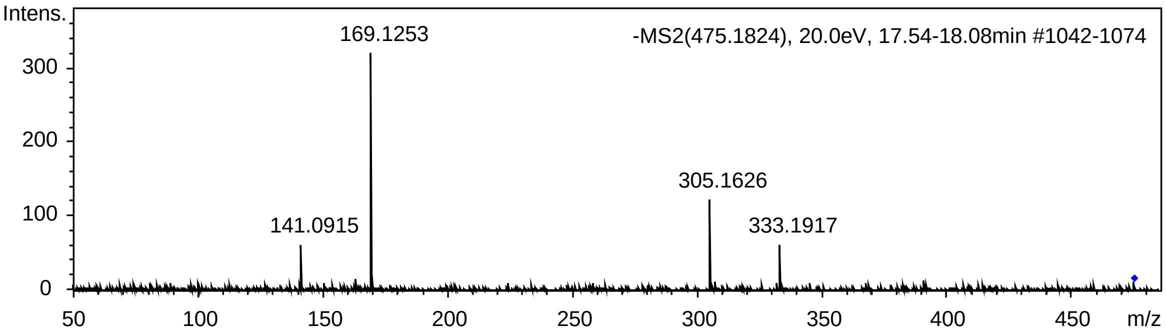


| Measured m/z | Possible MF | Possible structural assignment |
|---|---|---|
| 141.0915 | $C_8H_{13}O_2^{–}$ | FA (8:0) – $H_2O$ |
| 169.1253 | $C_{10}H_{17}O_2^{–}$ | FA (10:0) – $H_2O$ |
| 305.1626 | $C_{14}H_{25}O_7^{–}$ | monorhamnolipid (8:0/10:0) – FA (10:0) |
| 333.1917 | $C_{16}H_{29}O_7^{–}$ | monorhamnolipid (8:0/10:0) – FA (8:0) |

Monorhamnolipid (10:0/10:0) – $[M - H]^-$ 503.3220 at 22.3 min (standard)

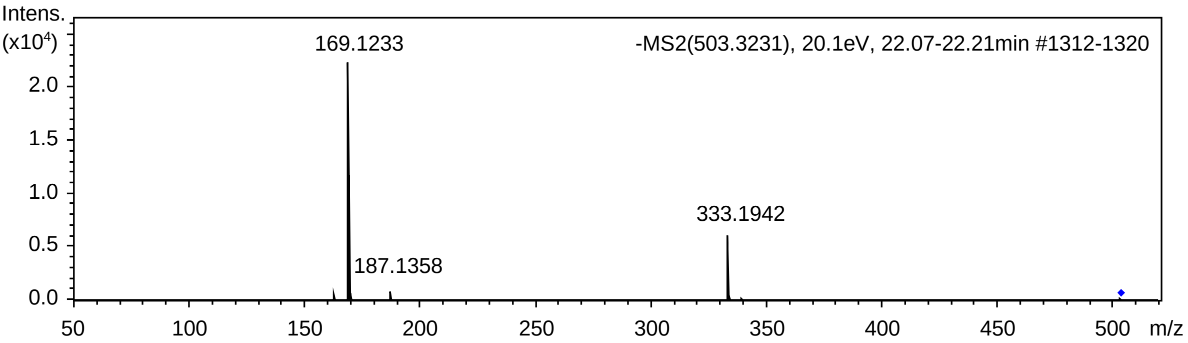


| Measured m/z | Possible MF | Possible structural assignment |
|---|---|---|
| 169.1233 | $C_{10}H_{17}O_2^-$ | FA (10:0) – $H_2O$ |
| 187.1358 | $C_{10}H_{19}O_3^-$ | FA (10:0) |
| 333.1942 | $C_{16}H_{29}O_7^-$ | monorhamnolipid (10:0/10:0) – FA (10:0) |

Monorhamnolipid (10:0/12:1) – $[M – H]^-$ 529.3377 at 23.5 min (standard)

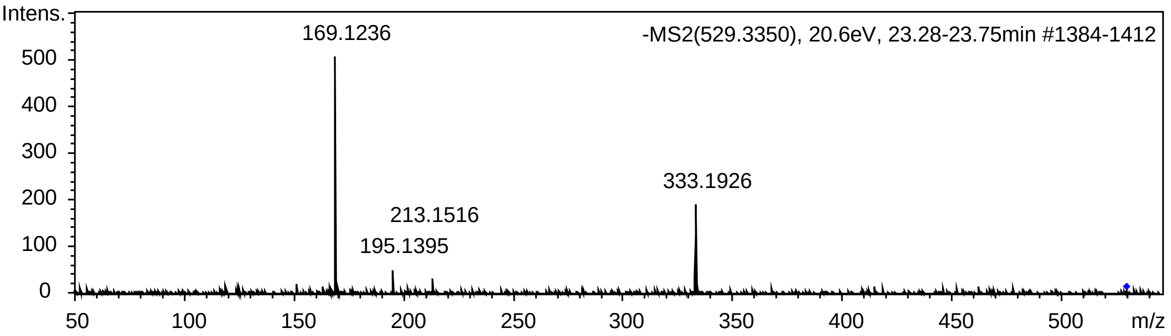


| Measured m/z | Possible MF | Possible structural assignment |
|---|---|---|
| 169.1236 | $C_{10}H_{17}O_2^-$ | FA (10:0) – $H_2O$ |
| 195.1395 | $C_{12}H_{19}O_2^-$ | FA (12:1) – $H_2O$ |
| 213.1516 | $C_{12}H_{21}O_3^-$ | FA (12:1) |
| 333.1926 | $C_{16}H_{29}O_7^-$ | monorhamnolipid (10:0/12:1) – FA (12:1) |

Monorhamnolipid (10:0/12:0) – $[M – H]^-$ 531.3533 at 22.3 min (standard)

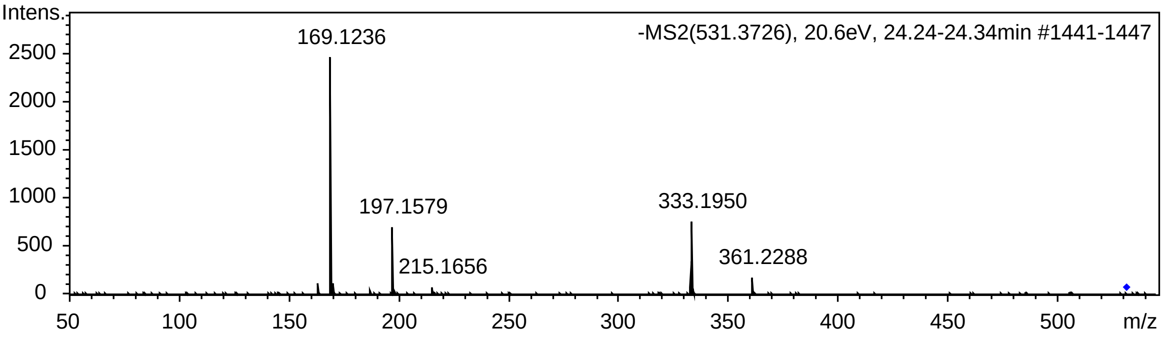


| Measured m/z | Possible MF | Possible structural assignment |
|---|---|---|
| 169.1236 | $C_{10}H_{17}O_2^-$ | FA (10:0) – $H_2O$ |
| 197.1579 | $C_{12}H_{21}O_2^-$ | FA (12:0) – $H_2O$ |
| 215.1656 | $C_{12}H_{23}O_3^-$ | FA (12:0) |
| 333.1950 | $C_{16}H_{29}O_7^-$ | monorhamnolipid (10:0/12:0) – FA (12:0) |
| 361.2288 | $C_{18}H_{33}O_7^-$ | monorhamnolipid (10:0/12:0) – FA (10:0) |

Dirhamnolipid (8:0/10:0) – $[M – H]^-$ 621.3486 at 17.7 min (standard)

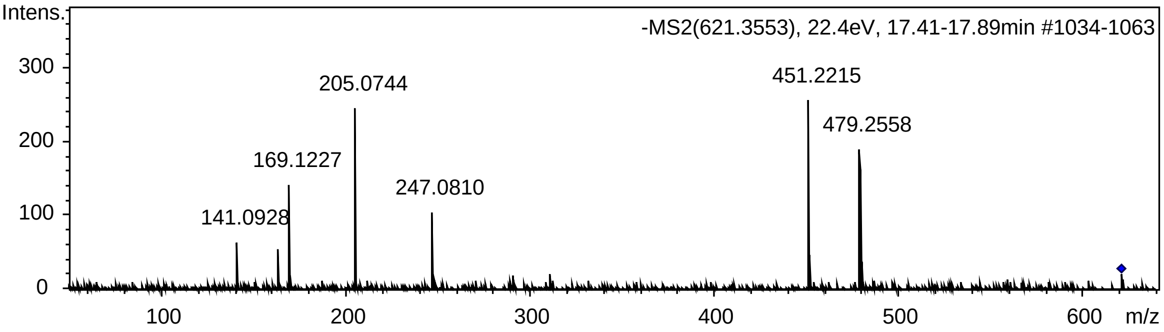


| Measured m/z | Possible MF | Possible structural assignment |
|---|---|---|
| 141.0928 | $C_8H_{13}O_2^-$ | FA (8:0) – $H_2O$ |
| 163.0619 | $C_6H_{11}O_5^-$ | rhamnose |
| 169.1227 | $C_{10}H_{17}O_2^-$ | FA (10:0) – $H_2O$ |
| 205.0744 | $C_8H_{13}O_6^-$ | rhamnose + $C_2H_2O$ |
| 247.0810 | $C_{10}H_{15}O_7^-$ | rhamnose + $C_4H_4O_2$ |
| 451.2215 | $C_{20}H_{35}O_{11}^-$ | dirhamnolipid (8:0/10:0) – FA (10:0) |
| 479.2558 | $C_{22}H_{39}O_{11}^-$ | dirhamnolipid (8:0/10:0) – FA (8:0) |

Dirhamnolipid (10:0/10:0) – $[M – H]^{–}$ 649.3799 at 22.1 min (standard)

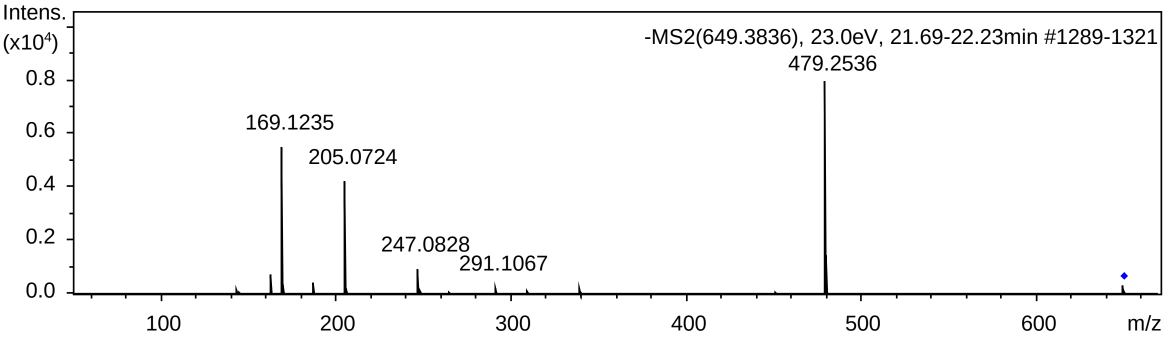


| Measured m/z | Possible MF | Possible structural assignment |
|---|---|---|
| 163.0662 | $C_6H_{11}O_5^–$ | rhamnose |
| 169.1276 | $C_{10}H_{17}O_2^–$ | FA (10:0) – $H_2O$ |
| 187.1363 | $C_{10}H_{19}O_3^–$ | FA (10:0) |
| 205.0743 | $C_8H_{13}O_6^–$ | rhamnose + $C_2H_2O$ |
| 247.0849 | $C_{10}H_{15}O_7^–$ | rhamnose + $C_4H_4O_2$ |
| 291.1089 | $C_{12}H_{19}O_8^–$ | dirhamnose – $H_2O$ |
| 309.1223 | $C_{12}H_{21}O_9^–$ | dirhamnose |
| 479.2571 | $C_{22}H_{39}O_{11}^–$ | dirhamnolipid (10:0/10:0) – FA (10:0) |

Dirhamnolipid (10:0/12:1) – $[M – H]^-$ 675.3956 at 23.6 min (standard)

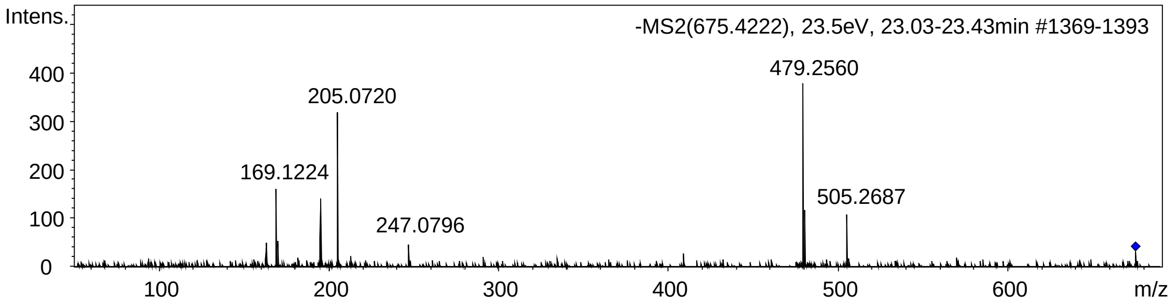


| Measured m/z | Possible MF | Possible structural assignment |
|---|---|---|
| 163.0610 | $C_6H_{11}O_5^-$ | rhamnose |
| 169.1224 | $C_{10}H_{17}O_2^-$ | FA (10:0) – $H_2O$ |
| 195.1416 | $C_{12}H_{19}O_2^-$ | FA (12:1) – $H_2O$ |
| 205.0720 | $C_8H_{13}O_6^-$ | rhamnose + $C_2H_2O$ |
| 247.0796 | $C_{10}H_{15}O_7^-$ | rhamnose + $C_4H_4O_2$ |
| 479.2560 | $C_{22}H_{39}O_{11}^-$ | dirhamnolipid (10:0/12:1) – FA (12:1) |
| 505.2687 | $C_{24}H_{41}O_{11}^-$ | dirhamnolipid (10:0/12:1) – FA (10:0) |

Dirhamnolipid (10:0/12:0) – $[M – H]^-$ 677.4112 at 24.0 min (standard)

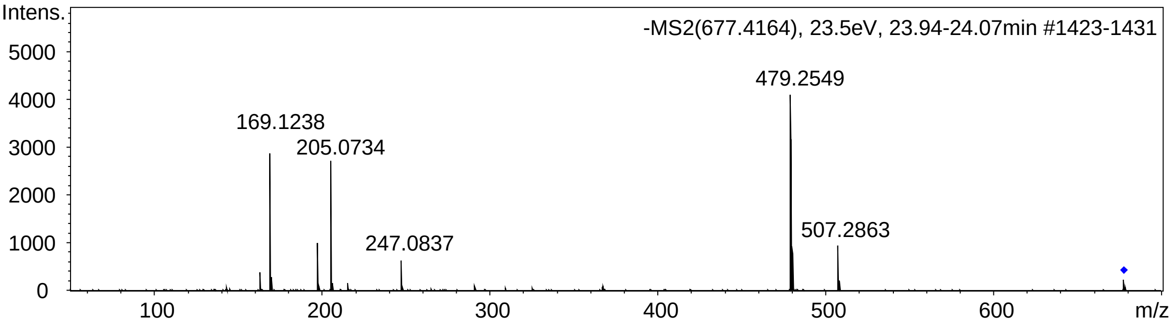


| Measured m/z | Possible MF | Possible structural assignment |
|---|---|---|
| 163.0618 | $C_6H_{11}O_5^-$ | rhamnose |
| 169.1238 | $C_{10}H_{17}O_2^-$ | FA (10:0) – $H_2O$ |
| 197.1563 | $C_{12}H_{21}O_2^-$ | FA (12:0) – $H_2O$ |
| 205.0734 | $C_8H_{13}O_6^-$ | rhamnose + $C_2H_2O$ |
| 247.0837 | $C_{10}H_{15}O_7^-$ | rhamnose + $C_4H_4O_2$ |
| 479.2549 | $C_{22}H_{39}O_{11}^-$ | dirhamnolipid (10:0/12:0) – FA (12:0) |
| 507.2863 | $C_{24}H_{43}O_{11}^-$ | dirhamnolipid (10:0/12:0) – FA (10:0) |

Dirhamnolipid (12:0/12:0) – $[M - H]^-$ 705.4431 at 26.1 min (standard)

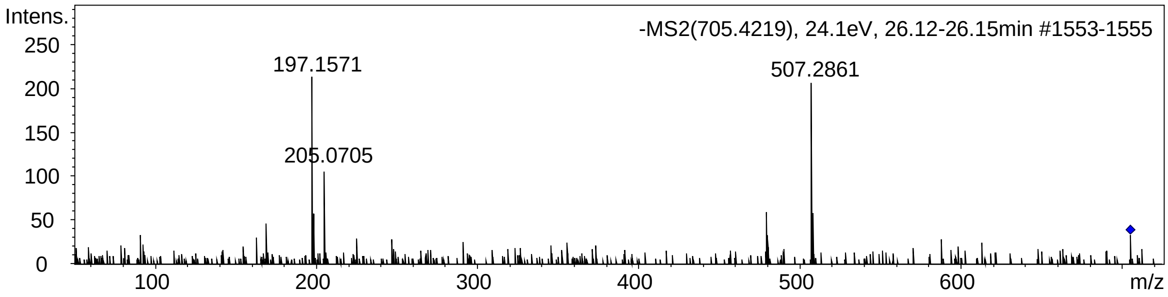


| Measured m/z | Possible MF | Possible structural assignment |
|---|---|---|
| 197.1571 | $C_{12}H_{21}O_2^-$ | FA (12:0) – $H_2O$ |
| 205.0705 | $C_8H_{13}O_6^-$ | rhamnose + $C_2H_2O$ |
| 507.2861 | $C_{24}H_{43}O_{11}^-$ | dirhamnolipid (12:0/12:0) – FA (12:0) |

**Positive-mode ions that show elevated levels in 100-Hz-stimulated intracellular samples**

m/z 270.1867 at 27.1 min – $[M + H]^+$ of 2-alkyl-4-hydroxyquinoline (AHQ (9:1))

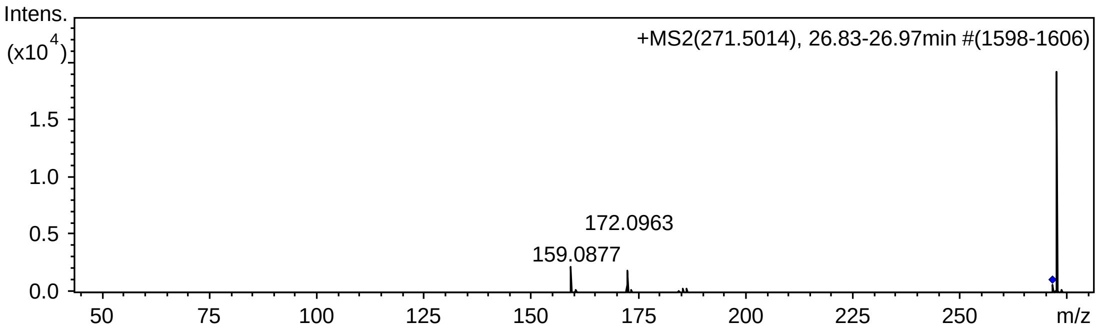


| Measured m/z | Possible MF | Possible structural assignment |
|---|---|---|
| 159.0877 | $C_{10}H_9NO^+$ | AHQ (9:1) – $C_8H_{15}$ |
| 172.0963 | $C_{11}H_{10}NO^+$ | AHQ (9:1) – $C_7H_{14}$ |
| 185.0993 | $C_{12}H_{11}NO^+$ | AHQ (9:1) – $C_6H_{13}$ |

m/z 272.2028 at 27.2 min – $[M + H]^+$ of 2-alkyl-4-hydroxyquinoline (AHQ (9:0); HNQ)

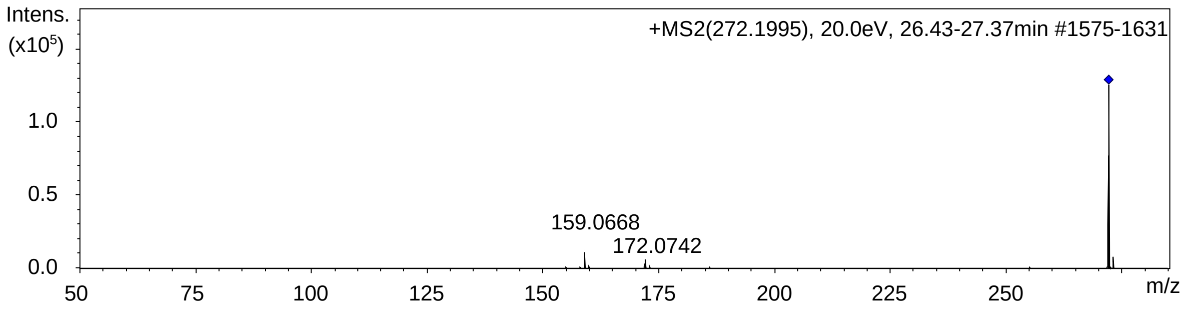


| Measured m/z | Possible MF | Possible structural assignment |
|---|---|---|
| 159.0668 | $C_{10}H_9NO^+$ | HNQ – $C_8H_{17}$ |
| 172.0742 | $C_{11}H_{10}NO^+$ | HNQ – $C_7H_{16}$ |
| 186.0881 | $C_{12}H_{12}NO^+$ | HNQ – $C_6H_{14}$ |

**Negative-mode ions that show elevated levels in 100-Hz-stimulated intracellular samples**

m/z 258.1484 at 13.7 min – $[M – H]^–$ of 2-alkyl-4-hydroxyquinoline *N*-oxide (AQNO (7:0); HQNO)

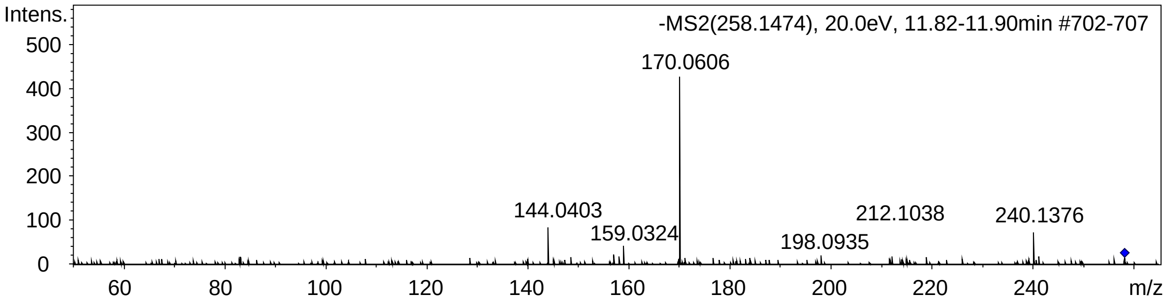


| Measured m/z | Possible MF | Possible structural assignment |
|---|---|---|
| 144.0403 | $C_9H_6NO^–$ | 4-hydroxyquinoline |
| 159.0324 | $C_9H_5NO_2^–$ | 4-hydroxyquinoline *N*-oxide |
| 170.0606 | $C_{11}H_8NO^–$ | HQNO – $H_2O$ – $C_5H_{10}$ |
| 198.0935 | $C_{13}H_{12}NO^–$ | HQNO – $H_2O$ – $C_3H_6$ |
| 212.1038 | $C_{14}H_{14}NO^–$ | HQNO – $H_2O$ – $C_2H_4$ |
| 240.1376 | $C_{16}H_{18}NO^–$ | HQNO – $H_2O$ |

m/z 268.1687 at 27.5 min – $[M-H]^-$ of 2-alkyl-4-hydroxyquinoline (AHQ (9:1))

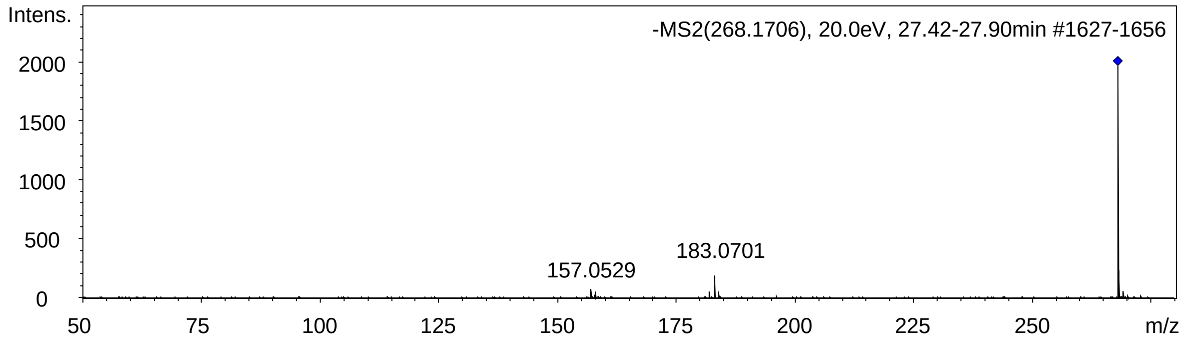


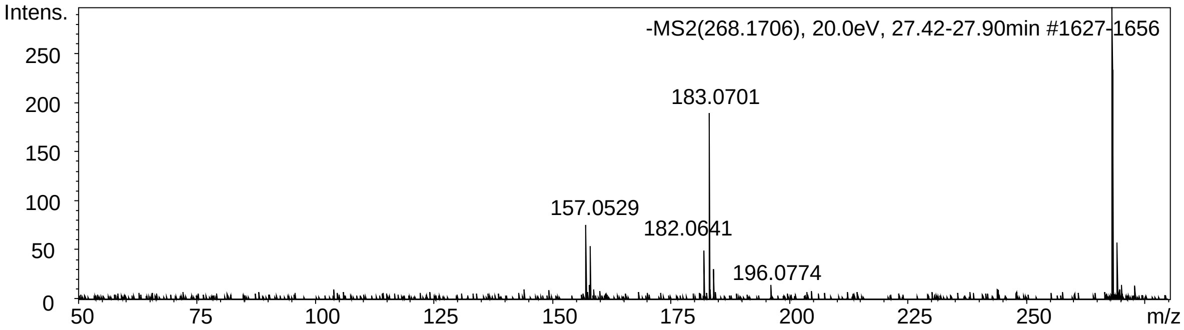


| Measured m/z | Possible MF | Possible structural assignment |
|---|---|---|
| 157.0529 | $C_{10}H_7NO^-$ | AHQ (9:1) – $C_8H_{15}$ |
| 182.0637 | $C_{12}H_8NO^-$ | AHQ (9:1) – $C_6H_{14}$ |
| 183.0700 | $C_{12}H_9NO^-$ | AHQ – $C_6H_{13}$ |
| 196.0774 | $C_{13}H_{10}NO^-$ | AHQ – $C_5H_{12}$ |

m/z 269.2107 at 17.0 min – [M – H]$^{-}$ of hydroxy FA (16:1)

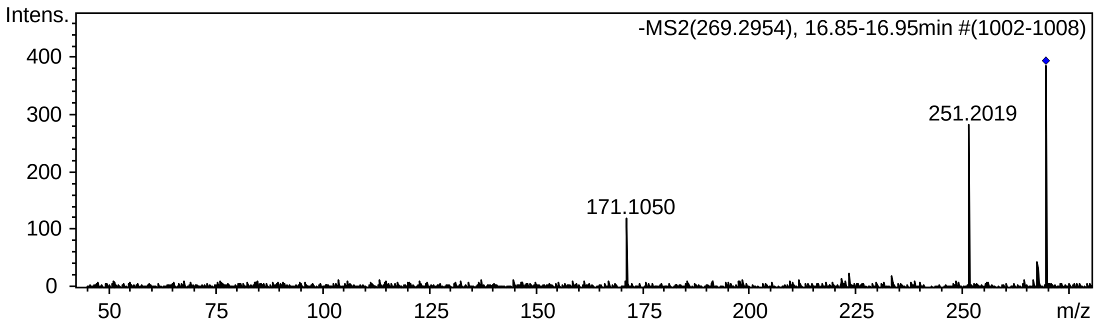


| Measured m/z | Possible MF | Possible structural assignment |
|---|---|---|
| 171.1050 | $C_9H_{15}O_3^-$ | hydroxy FA (16:1) – alkyl fragment (with the breakage at the double bond between $C_9$ and $C_{10}$) |
| 251.2019 | $C_{16}H_{27}O_2^-$ | hydroxy FA (16:1) – $H_2O$ |

m/z 286.1785 at 29.9 min – $[M - H]^-$ of 2-nonyl-3-hydroxy-4-quinolone (9:0) ($C_n$-PQS (9:0))

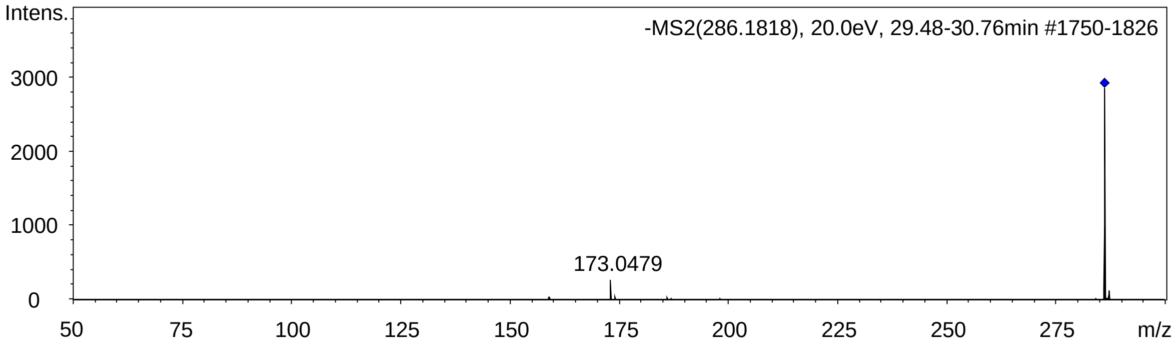


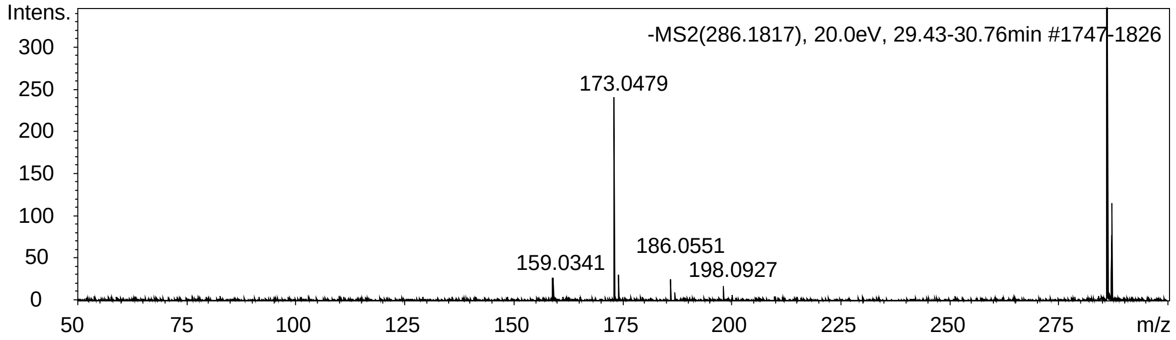


| Measured m/z | Possible MF | Possible structural assignment |
|---|---|---|
| 159.0341 | $C_9H_5NO_2^-$ | 3-hydroxy-4-quinolone |
| 173.0479 | $C_{10}H_7NO_2^-$ | $C_n$-PQS (9:0) – $C_8H_{17}$ |
| 186.0551 | $C_{11}H_8NO_2^-$ | $C_n$-PQS (9:0) – $C_7H_{16}$ |
| 198.0927 | $C_{13}H_{12}NO^-$ | $C_n$-PQS (9:0) – $C_5H_{12}$ |

m/z 297.2451 at 18.0 min – [M – H]$^-$ of hydroxy FA (18:1)

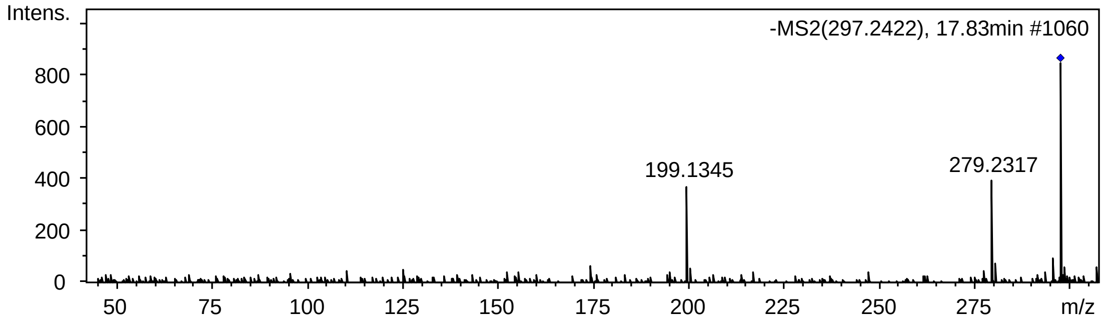


| Measured m/z | Possible MF | Possible structural assignment |
|---|---|---|
| 199.1345 | $C_{11}H_{19}O_3^-$ | hydroxy FA (18:1) – alkyl fragment (with the breakage at the double bond between $C_{11}$ and $C_{12}$) |
| 279.2317 | $C_{18}H_{31}O_2^-$ | hydroxy FA (18:1) – $H_2O$ |

m/z 312.1961 at 18.1 min – [M – H]$^{-}$ of 2-alkyl-4-hydroxyquinoline *N*-oxide (AQNO (11:1))

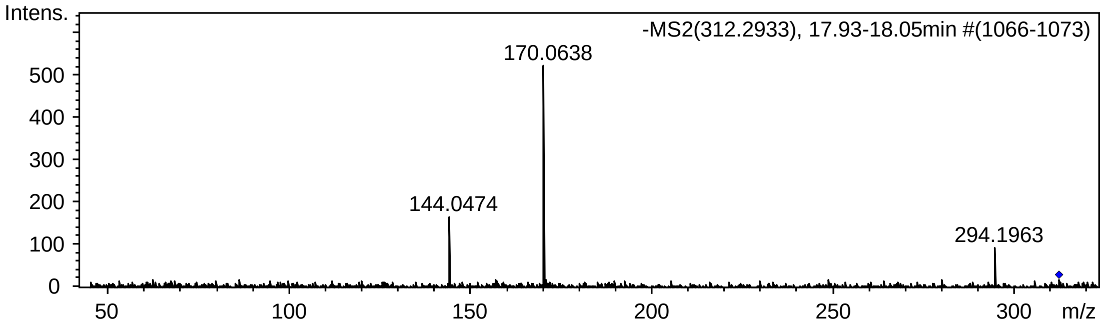


| Measured m/z | Possible MF | Possible structural assignment |
|---|---|---|
| 144.0474 | $C_9H_6NO^-$ | 4-hydroxyquinoline |
| 170.0638 | $C_{11}H_8NO^-$ | AQNO (11:1) – $H_2O$ – $C_9H_{16}$ |
| 294.1963 | $C_{20}H_{24}NO^-$ | AQNO (11:1) – $H_2O$ |

m/z 319.2243 at 18.0 min – $[M – H]^–$ of hydroxy FA (20:4)

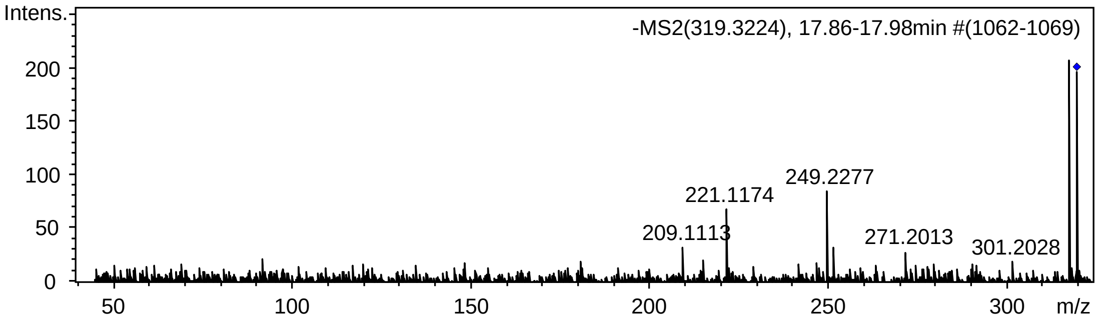


| Measured m/z | Possible MF | Possible structural assignment |
|---|---|---|
| 209.1113 | $C_{12}H_{17}O_3^–$ | hydroxy FA (20:4) – alkyl fragment (with the breakage at the double bond between $C_{12}$ and $C_{13}$) |
| 221.1174 | $C_{13}H_{17}O_3^–$ | hydroxy FA (20:4) – alkyl fragment (with the breakage at the double bond between $C_{13}$ and $C_{14}$) |
| 249.2277 | $C_{17}H_{29}O^–$ | ? |
| 271.2013 | $C_{19}H_{27}O^–$ | ? |
| 301.2028 | $C_{20}H_{29}O_2^–$ | hydroxy FA (20:4) – $H_2O$ |

m/z 324.2870 at 37.6 min – [M – H]$^{-}$ of *N*-acylethanolamine (18:1)

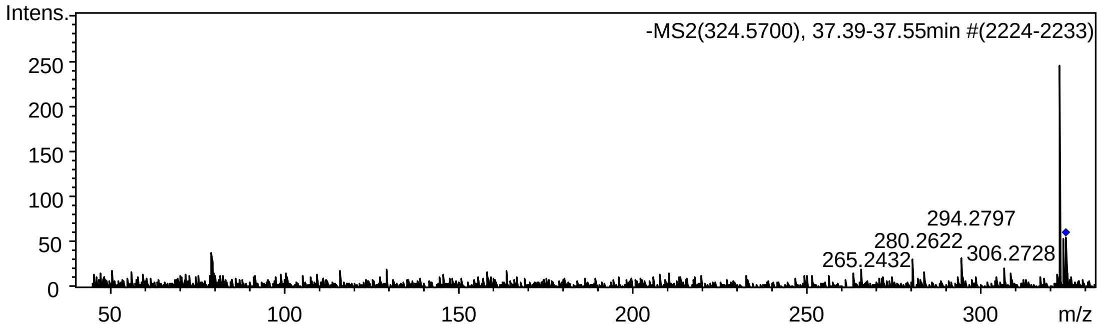


| Measured m/z | Possible MF | Possible structural assignment |
|---|---|---|
| 265.2432 | $C_{18}H_{33}O^{-}$ | *N*-acylethanolamine (18:1) – ethanolamine |
| 280.2622 | $C_{18}H_{34}NO^{-}$ | *N*-acylethanolamine (18:1) – ethanol |
| 294.2797 | $C_{19}H_{36}NO^{-}$ | *N*-acylethanolamine (18:1) – methanol |
| 306.2728 | $C_{20}H_{36}NO^{-}$ | *N*-acylethanolamine (18:1) – $H_2O$ |

m/z 338.3017 at 38.1 min – $[M - H]^-$ of *N*-acylethanolamine (21:1)

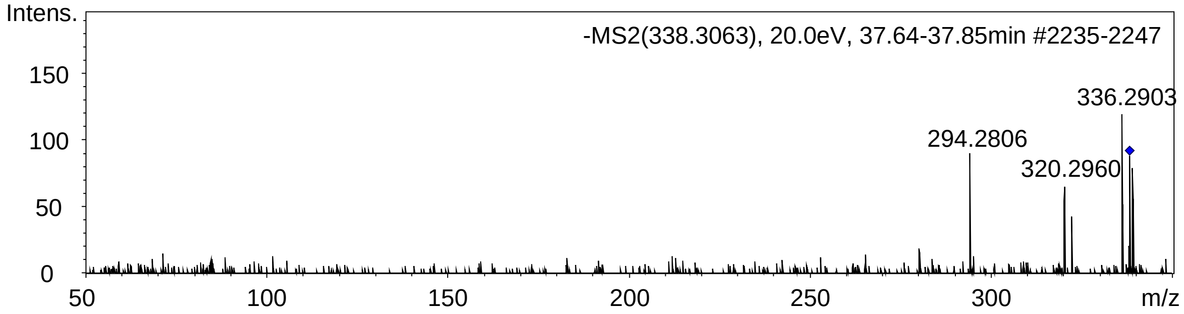


| Measured m/z | Possible MF | Possible structural assignment |
|---|---|---|
| 294.2797 | $C_{19}H_{36}NO^-$ | *N*-acylethanolamine (19:1) – ethanol |
| 320.2960 | $C_{21}H_{38}NO^-$ | *N*-acylethanolamine (19:1) – $H_2O$ |

m/z 354.2983 at 27.7 min – [M – H]$^-$ of *N*-acyl valine (16:0)

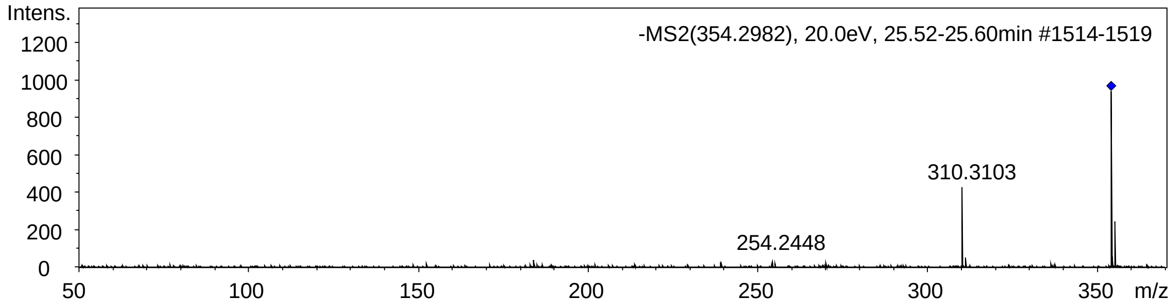


| Measured m/z | Possible MF | Possible structural assignment |
| --- | --- | --- |
| 254.2448 | $C_{16}H_{32}NO^-$ | 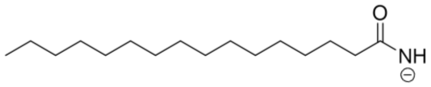 |
| 310.3103 | $C_{20}H_{40}NO^-$ | *N*-acyl valine (16:0) – $CO_2$ |

m/z 380.3133 at 28.3 min – [M – H]$^{–}$ of *N*-acyl valine (18:1)

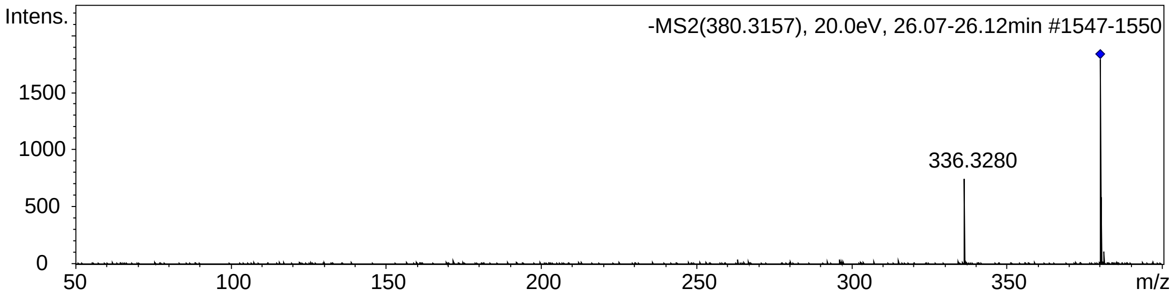


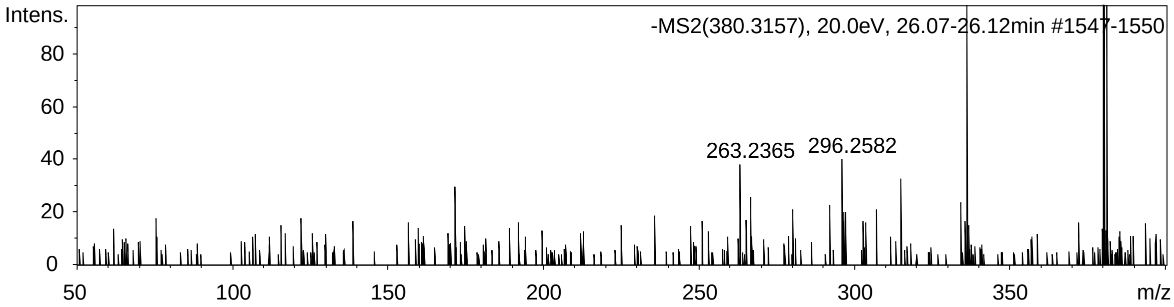


| Measured m/z | Possible MF | Possible structural assignment |
|---|---|---|
| 263.2365 | $C_{18}H_{31}O^{–}$ | fatty amide (18:1) – $NH_3$ |
| 296.2582 | $C_{18}H_{34}NO_2^{–}$ | *N*-hydroxy fatty amide (18:1) (?) |
| 336.3280 | $C_{22}H_{42}NO^{–}$ | *N*-acyl valine (18:1) – $CO_2$ |

m/z 380.3147 at 20.6 min – $[M – H]^-$ of *N*-acyl leucine (17:1) (?)

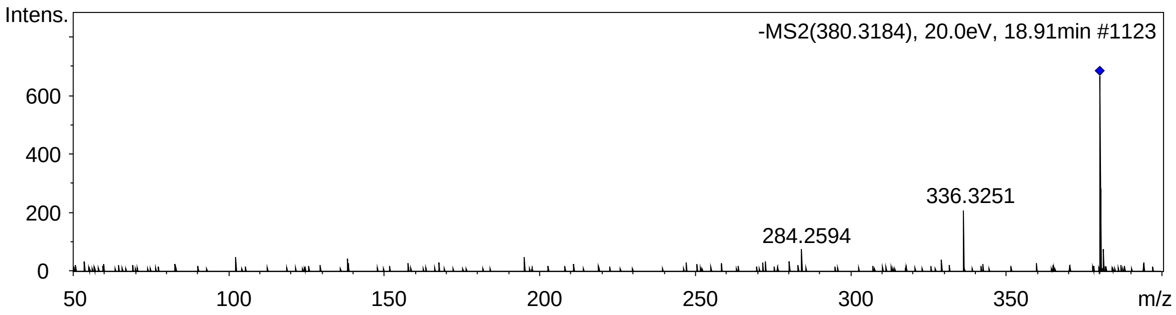


| Measured m/z | Possible MF | Possible structural assignment |
|---|---|---|
| 284.2594 | $C_{17}H_{34}NO_2^-$ | *N*-hydroxy fatty amide (17:1) + $H_2$ (?) |
| 336.3264 | $C_{22}H_{42}NO^-$ | *N*-acyl leucine (17:1) – $CO_2$ |

**Positive-mode ions that show decreased levels in 100-Hz-stimulated extracellular samples**

m/z 499.2879 at 29.0 min – $[M + Na]^+$ of monorhamnolipid (8:0/10:0)

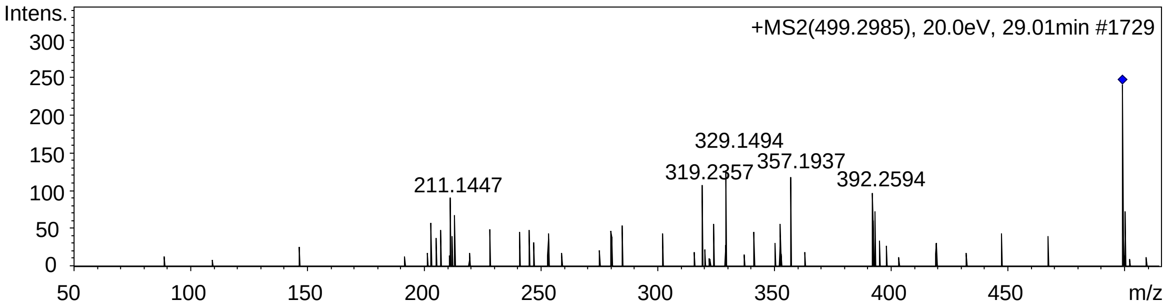


| Measured m/z | Possible MF | Possible structural assignment |
|---|---|---|
| 211.1447 | $C_{10}H_{20}NaO_3^+$ | hydroxy FA (10:0) (sodium adduct) |
| 329.1494 | $C_{14}H_{26}NaO_7^+$ | monorhamnolipid (8:0/10:0) – FA(10:0) (sodium adduct) |
| 357.1937 | $C_{16}H_{30}NaO_7^+$ | monorhamnolipid (8:0/10:0) – FA(8:0) (sodium adduct) |

m/z 527.3188 at 31.3 min – $[M + Na]^+$ of monorhamnolipid (10:0/10:0)

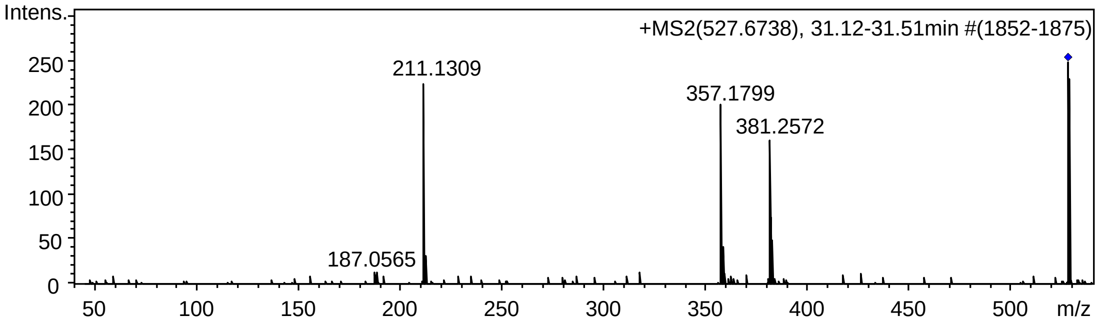


| Measured m/z | Possible MF | Possible structural assignment |
|---|---|---|
| 187.0565 | $C_6H_{12}NaO_5^+$ | rhamnose (sodium adduct) |
| 211.1309 | $C_{10}H_{20}NaO_3^+$ | hydroxy FA (10:0) (sodium adduct) |
| 357.1799 | $C_{16}H_{30}NaO_7^+$ | monorhamnolipid (10:0/10:0) – FA(10:0) (sodium adduct) |
| 381.2572 | $C_{20}H_{38}NaO_5^+$ | HAA (10:0/10:0) (sodium adduct) |

m/z 548.3781 at 32.8 min – $[M + NH_4]^+$ of monorhamnolipid (10:0/12:1)

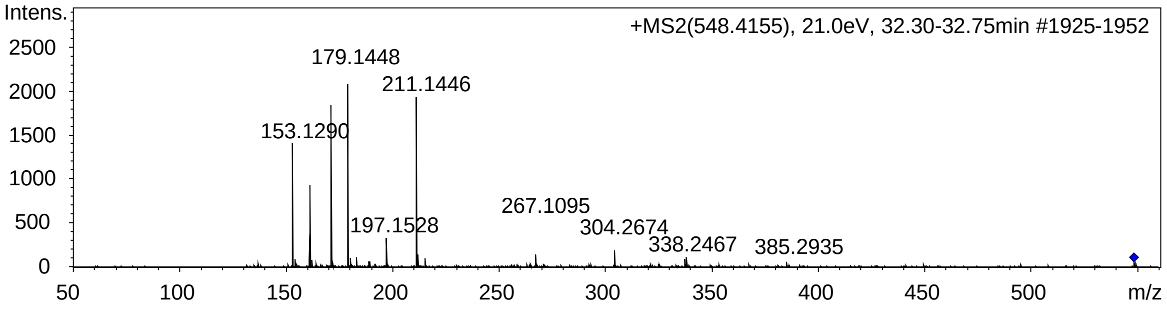


| Measured m/z | Possible MF | Possible structural assignment |
|---|---|---|
| 153.1290 | $C_{10}H_{17}O^+$ | acylium ion of hydroxy FA (10:0) – $H_2O$ |
| 161.1348 | $C_{12}H_{17}^+$ | acylium ion of hydroxy FA (12:1) – $2H_2O$ |
| 171.1397 | $C_{10}H_{19}O_2^+$ | acylium ion of hydroxy FA (10:0) |
| 179.1448 | $C_{12}H_{19}O^+$ | acylium ion of hydroxy FA (12:1) – $H_2O$ |
| 197.1528 | $C_{12}H_{21}O_2^+$ | acylium ion of hydroxy FA (12:1) |
| 385.2935 | $C_{22}H_{41}O_5^+$ | HAA (10:0/12:1) |

m/z 550.3937 at 33.2 min – $[M + NH_4]^+$ of monorhamnolipid (10:0/12:0)

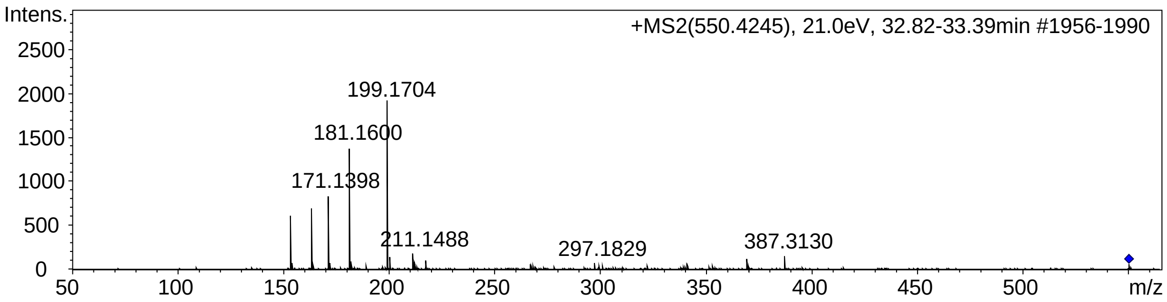


| Measured m/z | Possible MF | Possible structural assignment |
|---|---|---|
| 153.1284 | $C_{10}H_{17}O^+$ | acylium ion of hydroxy FA (10:0) – $H_2O$ |
| 163.1504 | $C_{12}H_{19}^+$ | acylium ion of hydroxy FA (12:0) – $2H_2O$ |
| 171.1398 | $C_{10}H_{19}O_2^+$ | acylium ion of hydroxy FA (10:0) |
| 181.1600 | $C_{12}H_{21}O^+$ | acylium ion of hydroxy FA (12:0) – $H_2O$ |
| 199.1704 | $C_{12}H_{23}O_2^+$ | acylium ion of hydroxy FA (12:0) |
| 369.3016 | $C_{22}H_{41}O_4^+$ | HAA (10:0/12:0) – $H_2O$ |
| 387.3130 | $C_{22}H_{43}O_5^+$ | HAA (10:0/12:0) |

m/z 553.3331 at 32.8 min – $[M + Na]^+$ of monorhamnolipid (10:0/12:1)

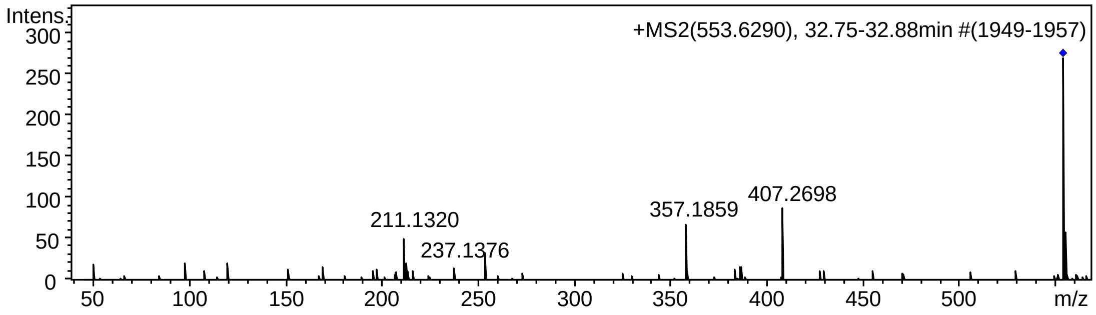


| Measured m/z | Possible MF | Possible structural assignment |
|---|---|---|
| 211.1320 | $C_{10}H_{20}NaO_3^+$ | hydroxy FA (10:0) (sodium adduct) |
| 237.1376 | $C_{12}H_{22}NaO_3^+$ | hydroxy FA (12:1) (sodium adduct) |
| 357.1859 | $C_{16}H_{30}NaO_7^+$ | monorhamnolipid (10:0/12:1) – FA(12:1) |
| 407.2698 | $C_{22}H_{40}NaO_5^+$ | HAA (10:0/12:1) (sodium adduct) |

m/z 555.3493 at 33.2 min – $[M + Na]^+$ of monorhamnolipid (10:0/12:0)

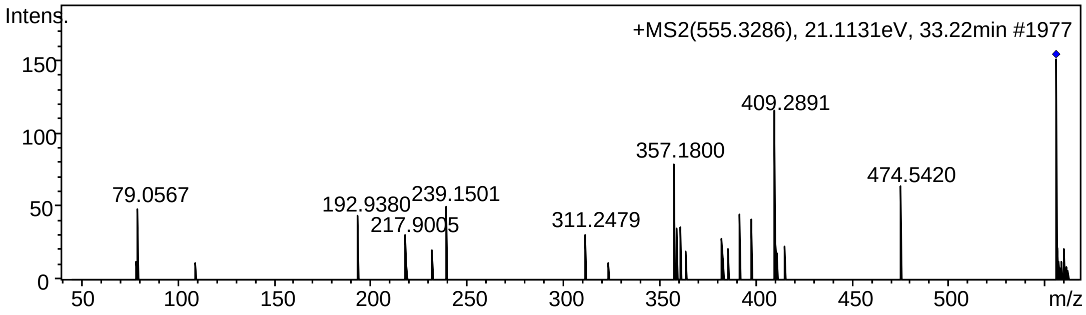


| Measured m/z | Possible MF | Possible structural assignment |
|---|---|---|
| 79.0567 | $C_4H_8Na^+$ | alkyl fragment |
| 239.1501 | $C_{12}H_{24}NaO_3^+$ | hydroxy FA (12:0) (sodium adduct) |
| 357.1800 | $C_{16}H_{30}NaO_7^+$ | monorhamnolipid (10:0/12:0) – FA(12:0) |
| 409.2891 | $C_{22}H_{42}NaO_5^+$ | HAA (10:0/12:0) (sodium adduct) |

m/z 668.4206 at 30.4 min – $[M + NH_4]^+$ of dirhamnolipid (10:0/10:0)

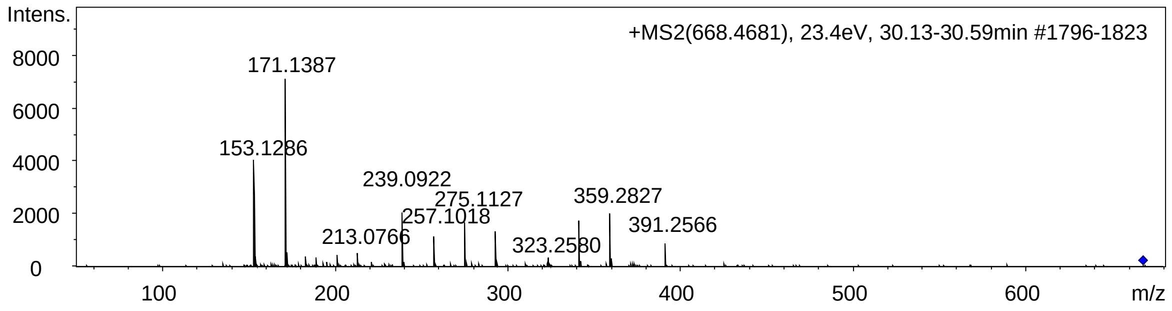


| Measured m/z | Possible MF | Possible structural assignment |
|---|---|---|
| 153.1286 | $C_{10}H_{17}O^+$ | acylium ion of hydroxy FA (10:0) – $H_2O$ |
| 171.1387 | $C_{10}H_{19}O_2^+$ | acylium ion of hydroxy FA (10:0) |
| 189.1499 | $C_{10}H_{21}O_3^+$ | hydroxy FA (10:0) |
| 201.0784 | $C_7H_{14}NaO_5^+$ | rhamnose + $CH_2$ (sodium adduct) |
| 213.0766 | $C_8H_{14}NaO_5^+$ | rhamnose + $C_2H_2$ (sodium adduct) |
| 239.0922 | $C_{10}H_{16}NaO_5^+$ | rhamnose + $C_4H_4$ (sodium adduct) |
| 257.1018 | $C_{10}H_{18}NaO_6^+$ | rhamnose + $C_4H_6O$ (sodium adduct) |
| 275.1127 | $C_{10}H_{20}NaO_7^+$ | rhamnose + $C_4H_8O_2$ (sodium adduct) |
| 293.1223 | $C_{10}H_{22}NaO_8^+$ | rhamnose + $C_4H_{10}O_3$ (sodium adduct) |
| 323.2580 | $C_{20}H_{35}O_3^+$ | HAA (10:0/10:0) – $2H_2O$ |
| 341.2688 | $C_{20}H_{37}O_4^+$ | HAA (10:0/10:0) – $H_2O$ |
| 359.2827 | $C_{20}H_{39}O_5^+$ | HAA (10:0/10:0) |

m/z 696.4523 at 32.4 min – $[M + NH_4]^+$ of dirhamnolipid (10:0/12:0)

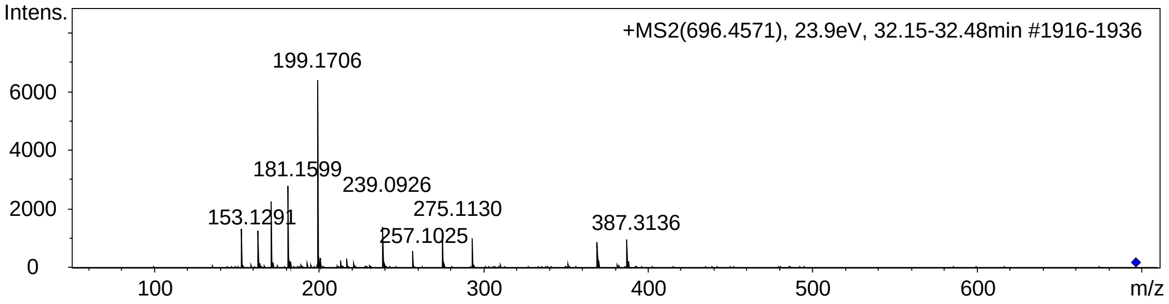


| Measured m/z | Possible MF | Possible structural assignment |
|---|---|---|
| 153.1291 | $C_{10}H_{17}O^+$ | acylium ion of hydroxy FA (10:0) – $H_2O$ |
| 163.1498 | $C_{12}H_{19}^+$ | hydroxy FA (12:0) – $2H_2O$ |
| 171.1386 | $C_{10}H_{19}O_2^+$ | acylium ion of hydroxy FA (10:0) |
| 181.1599 | $C_{12}H_{21}O^+$ | acylium ion of hydroxy FA (12:0) – $H_2O$ |
| 199.1706 | $C_{12}H_{23}O_2^+$ | acylium ion of hydroxy FA (12:0) |
| 213.0775 | $C_8H_{14}NaO_5^+$ | rhamnose + $C_2H_2$ (sodium adduct) |
| 239.0926 | $C_{10}H_{16}NaO_5^+$ | rhamnose + $C_4H_4$ (sodium adduct) |
| 257.1025 | $C_{10}H_{18}NaO_6^+$ | rhamnose + $C_4H_6O$ (sodium adduct) |
| 275.1130 | $C_{10}H_{20}NaO_7^+$ | rhamnose + $C_4H_8O_2$ (sodium adduct) |
| 293.1255 | $C_{10}H_{22}NaO_8^+$ | rhamnose + $C_4H_{10}O_3$ (sodium adduct) |
| 369.3023 | $C_{22}H_{41}O_4^+$ | HAA (10:0/12:0) – $H_2O$ |
| 387.3136 | $C_{22}H_{43}O_5^+$ | HAA (10:0/12:0) |

**Negative-mode ions that show decreased levels in 100-Hz-stimulated extracellular samples**

m/z 503.3304 at 23.9 min – $[M – H]^{–}$ of monorhamnolipid (10:0/10:0)

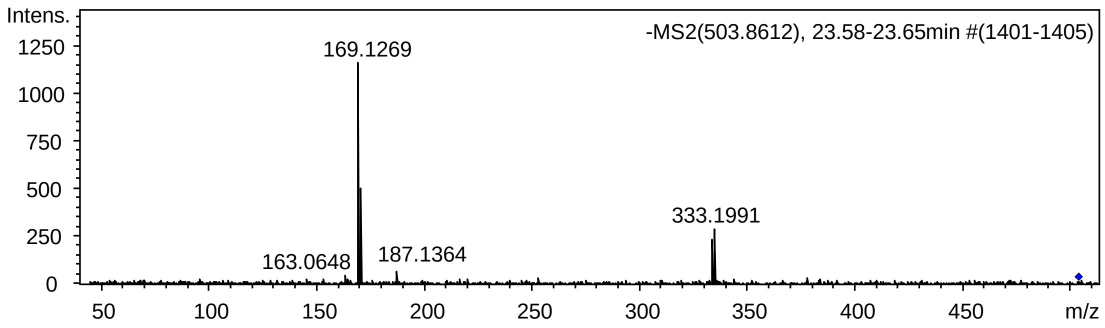


| Measured m/z | Possible MF | Possible structural assignment |
|---|---|---|
| 163.0648 | $C_6H_{11}O_5^{–}$ | rhamnose |
| 169.1269 | $C_{10}H_{17}O_2^{–}$ | FA (10:0) – $H_2O$ |
| 187.1364 | $C_{10}H_{19}O_3^{–}$ | FA (10:0) |
| 333.1991 | $C_{16}H_{29}O_7^{–}$ | monorhamnolipid (10:0/10:0) – FA (10:0) |

m/z 529.5742 at 25.3 min – $[M – H]^{–}$ of monorhamnolipid (10:0/12:1)

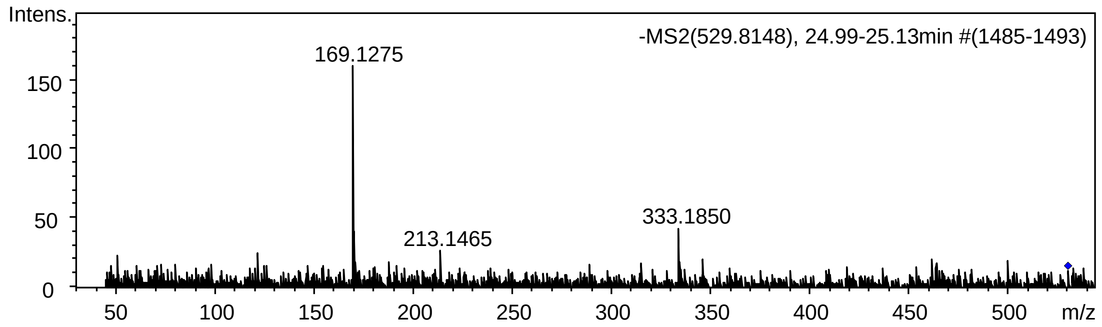


| Measured m/z | Possible MF | Possible structural assignment |
|---|---|---|
| 169.1275 | $C_{10}H_{17}O_2^{–}$ | FA (10:0) – $H_2O$ |
| 213.1465 | $C_{12}H_{21}O_3^{–}$ | FA (12:1) |
| 333.1850 | $C_{16}H_{29}O_7^{–}$ | monorhamnolipid (10:0/12:1) – FA (12:1) |

m/z 531.5864 at 26.2 min – [M – H]$^{–}$ of monorhamnolipid (10:0/12:0)

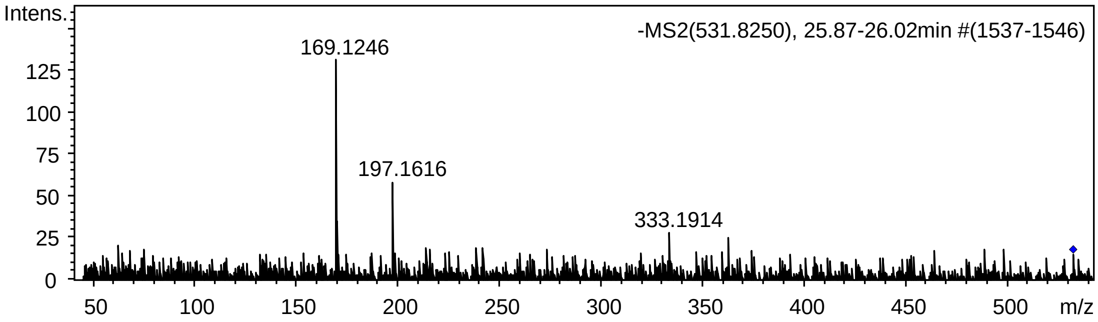


| Measured m/z | Possible MF | Possible structural assignment |
|---|---|---|
| 169.1246 | $C_{10}H_{17}O_2^{–}$ | FA (10:0) – $H_2O$ |
| 197.1616 | $C_{12}H_{21}O_2^{–}$ | FA (12:0) – $H_2O$ |
| 333.1914 | $C_{16}H_{29}O_7^{–}$ | monorhamnolipid (10:0/12:0) – FA (12:0) |

m/z 649.3862 at 23.7 min – $[M – H]^–$ of dirhamnolipid (10:0/10:0)

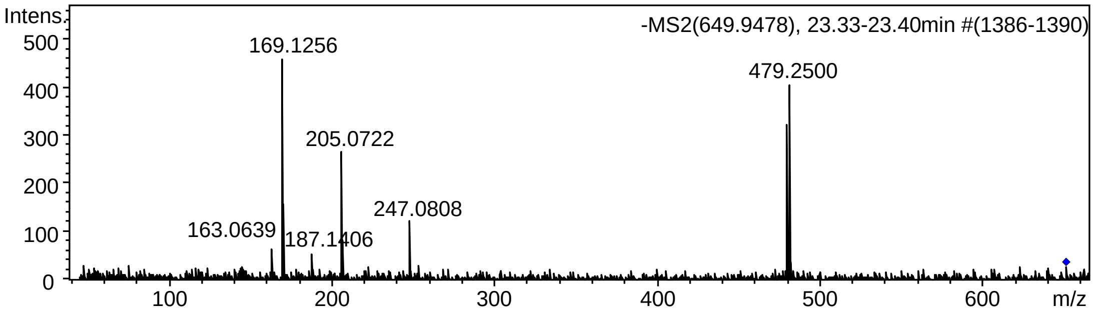


| Measured m/z | Possible MF | Possible structural assignment |
|---|---|---|
| 163.0639 | $C_6H_{11}O_5^–$ | rhamnose |
| 169.1256 | $C_{10}H_{17}O_2^–$ | FA (10:0) – $H_2O$ |
| 187.1406 | $C_{10}H_{19}O_3^–$ | FA (10:0) |
| 205.0722 | $C_8H_{13}O_6^–$ | rhamnose + $C_2H_2O$ |
| 247.0808 | $C_{10}H_{15}O_7^–$ | rhamnose + $C_4H_4O_2$ |
| 479.2500 | $C_{22}H_{39}O_{11}^–$ | dirhamnolipid (10:0/10:0) – FA (10:0) |

m/z 675.4021 at 25.0 min – $[M – H]^–$ of dirhamnolipid (10:0/12:1)

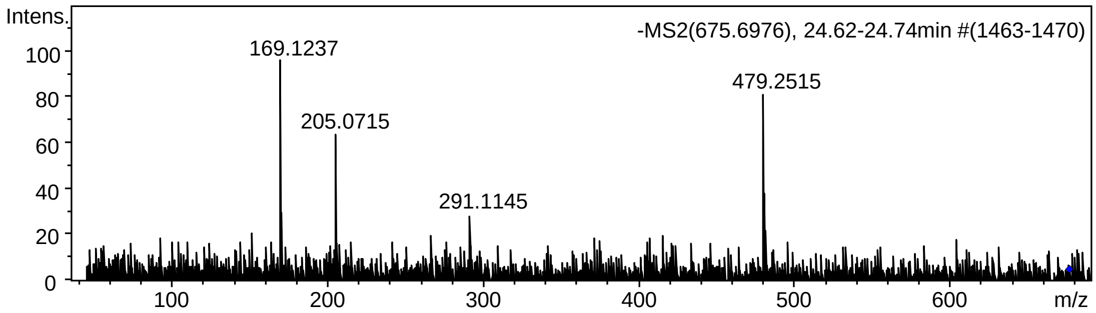


| Measured m/z | Possible MF | Possible structural assignment |
|---|---|---|
| 169.1237 | $C_{10}H_{17}O_2^–$ | FA (10:0) – $H_2O$ |
| 205.0715 | $C_8H_{13}O_6^–$ | rhamnose + $C_2H_2O$ |
| 291.1145 | $C_{12}H_{19}O_8^–$ | dirhamnose – $H_2O$ |
| 479.2515 | $C_{22}H_{39}O_{11}^–$ | dirhamnolipid (10:0/12:1) – FA (12:1) |

m/z 677.4191 at 25.9 min – [M – H]$^{-}$ of dirhamnolipid (10:0/12:0)

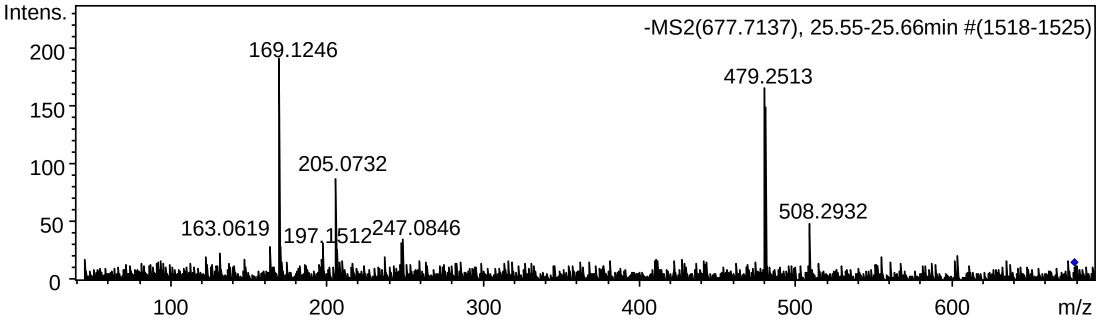


| Measured m/z | Possible MF | Possible structural assignment |
|---|---|---|
| 163.0619 | $C_6H_{11}O_5^-$ | rhamnose |
| 169.1246 | $C_{10}H_{17}O_2^-$ | FA (10:0) – $H_2O$ |
| 197.1512 | $C_{12}H_{21}O_2^-$ | FA (12:0) – $H_2O$ |
| 205.0732 | $C_8H_{13}O_6^-$ | rhamnose + $C_2H_2O$ |
| 247.0846 | $C_{10}H_{15}O_7^-$ | rhamnose + $C_4H_4O_2$ |
| 479.2513 | $C_{22}H_{39}O_{11}^-$ | dirhamnolipid (10:0/12:0) – FA (12:0) |

m/z 705.4486 at 28.2 min – $[M – H]^–$ of dirhamnolipid (12:0/12:0)

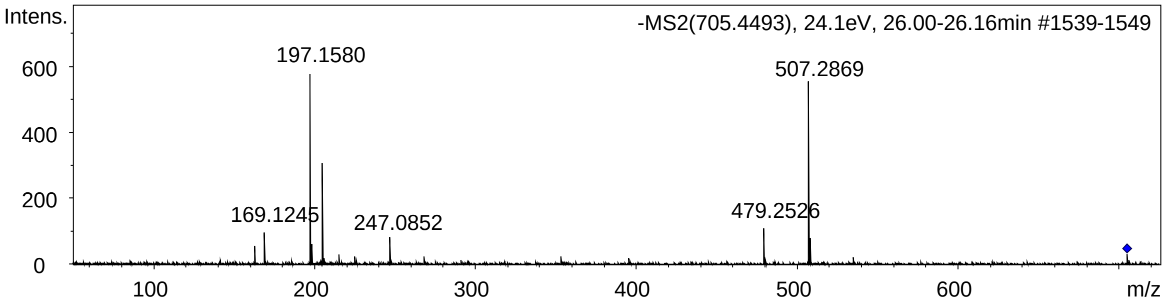


| Measured m/z | Possible MF | Possible structural assignment |
|---|---|---|
| 163.0625 | $C_6H_{11}O_5^–$ | rhamnose |
| 197.1580 | $C_{12}H_{21}O_2^–$ | FA (12:0) – $H_2O$ |
| 205.0744 | $C_8H_{13}O_6^–$ | rhamnose + $C_2H_2O$ |
| 247.2526 | $C_{10}H_{15}O_7^–$ | rhamnose + $C_4H_4O_2$ |
| 507.2869 | $C_{24}H_{43}O_{11}^–$ | dirhamnolipid (12:0/12:0) – FA (12:0) |

**S10 Table.** Oligonucleotides used as forward and reverse primers for amplification of target genes in the quantitative real-time polymerase chain reaction with the controls being *rpoD* and *proC* genes.

| Gene | Primer sequence (5’ to 3’) | Amplicon size (bp) |
|---|---|---|
| *fabB* (PA1609) | CTGGCTCCAGTGTTCCTCTTC<br>CAACTACTCGATCTCCTCCGC | 127 |
| *fabF* (PA2965) | CCTTGACCGAACCACCGAAG<br>CGTCATTACTGGCATGGGCA | 149 |
| *fabG* (PA2967) | CCGCATTCACGGTAATGGCA<br>CGCATCATCAACATCGGTTCC | 142 |
| *fabH3* (PA3286) | CTCGGGAAGGTACGGAACCA<br>CGTTCATCGAGAAGGCGTCC | 95 |
| *fabI* (PA1806) | TCACAGGGGAAGCACAGCTC<br>CCAGAACGACAAGCTCAGGG | 81 |
| *fabV* (PA2950) | TGACCAGGACCTTCTTCGGT<br>GTGGCTTCATCTGCGTCACTA | 117 |
| *fabY* (PA5174) | CGAACGCATCTTCGCCTCTA<br>GAGGACCTTGTGCCAGTGGA | 85 |
| *fabZ* (PA3645) | CTCGGGGAAATGTCCGTTGA<br>ACCTGCCTCATCGCTATCCT | 128 |
| *rhlA* (PA3479) | ACGGTCTCGTTGAGCAGATG<br>CATTCGCCCCTGGACTGAAC | 109 |
| *rhlC* (PA1130) | AGGAGATCAGGAACGAGGTGC<br>CATCCATCTCGACGGACTGACG | 83 |
| *rhlI* (PA3476) | ATGTAGCGGGTTTGCGGATG<br>GGTCTTCATCGAGAAGCTGGG | 96 |
| *rhlR* (PA3477) | GCCCCGTAGTTCTGCATCTG<br>CACACGATTCCCTTCACCCG | 92 |
| *rhlY* (PA0745) | GGTCACTACCAGGGCGTAGATA<br>GATCACCATCAACCACCCGC | 115 |
| *rmlB* (PA5161) | CTGGGCGAGACCTACAATGTC<br>GGACGATCCTTGACGAAGGTG | 146 |
| *fadB1* (PA1737) | AAAGACCAGCGACGAAACCC<br>GGTGAAGGTGCCGAATACCC | 121 |
| *fadB4* (PA4786) | TTCATCGAGACGCAGATGACC<br>GCCCTGGCTCATGGAGTTCA | 81 |
| *fadB5* (PA3014) | CACTGAGGGTGAGACGGTTG<br>GCCATCACGGTTAAGCCTCT | 97 |
| *fadD1* (PA3299) | GCTGCCAGTAGCCCTTCATC<br>ACCCTGTGCAAGGTGATTGG | 103 |
| *fadD2* (PA3300) | CAGCCTTCGGCATCGAGAAT<br>TGTGTGAAAGGTCCGCAAGT | 80 |

| Gene | Primer sequence (5’ to 3’) | Amplicon size (bp) |
|---|---|---|
| *fadD4* (PA1617) | GAAAGCCTGGACACCTTCGT<br>CACCCCCATCTGGAACTTGG | 96 |
| *fadE* (PA0506) | GCGTGATATCCGTTTCGTTCG<br>GCAGAACTTCGCACCTTCCT | 127 |
| *lasI* (PA1432) | CTCTGGACAGAAAGGCTCGC<br>CAGCGTCTGGATGTCGTTCT | 97 |
| *lasR* (PA1430) | TCAGCGTGGAAGCGGAAAAC<br>TGCAGTGCGTAGTCCTTGAG | 91 |
| *pqsA* (PA0996) | GAGGCGGTTCTGGTTCCTAC<br>TGTGCGAGGGAATCTGTTCG | 145 |
| *pqsH* (PA2587) | GGTGAAAAGACGCTGGTGGA<br>CTTGGTCAGTGGGAATCGCC | 102 |
| *pqsR* (PA1003) | GGTTCGTAGAGTTCGCTGAGG<br>AAAACTTCGACGACATGCTGC | 124 |
| *rpoS* (PA3622) | GATCGTCGGTGAGCGTATCC<br>CCTGAACGAACGGGTGACTT | 83 |
| *proC* (PA0393) (control) | GGAGACGATCAGTTGCTCCG<br>GTCGTGGTCCTGTCGGTCAA | 90 |
| *rpoD* (PA0576) (control) | GGTGTGGTCGGTGTTCATGT<br>GAGAGCCTCAAGGAATCCACC | 105 |
